# SAAS-CNV: A Joint Segmentation Approach on Aggregated and Allele Specific Signals for the Identification of Somatic Copy Number Alterations with Next-Generation Sequencing Data

**DOI:** 10.1371/journal.pcbi.1004618

**Published:** 2015-11-19

**Authors:** Zhongyang Zhang, Ke Hao

**Affiliations:** 1 Department of Genetics and Genomic Sciences, Icahn School of Medicine at Mount Sinai, New York, New York, United States of America; 2 Icahn Institute for Genomics and Multiscale Biology, Icahn School of Medicine at Mount Sinai, New York, New York, United States of America; 3 Department of Respiratory Medicine, Shanghai Tenth People’s Hospital, Tongji University, Shanghai, China; National Research Council of Canada, CANADA

## Abstract

Cancer genomes exhibit profound somatic copy number alterations (SCNAs). Studying tumor SCNAs using massively parallel sequencing provides unprecedented resolution and meanwhile gives rise to new challenges in data analysis, complicated by tumor aneuploidy and heterogeneity as well as normal cell contamination. While the majority of read depth based methods utilize total sequencing depth alone for SCNA inference, the allele specific signals are undervalued. We proposed a joint segmentation and inference approach using both signals to meet some of the challenges. Our method consists of four major steps: 1) extracting read depth supporting reference and alternative alleles at each SNP/Indel locus and comparing the total read depth and alternative allele proportion between tumor and matched normal sample; 2) performing joint segmentation on the two signal dimensions; 3) correcting the copy number baseline from which the SCNA state is determined; 4) calling SCNA state for each segment based on both signal dimensions. The method is applicable to whole exome/genome sequencing (WES/WGS) as well as SNP array data in a tumor-control study. We applied the method to a dataset containing no SCNAs to test the specificity, created by pairing sequencing replicates of a single HapMap sample as normal/tumor pairs, as well as a large-scale WGS dataset consisting of 88 liver tumors along with adjacent normal tissues. Compared with representative methods, our method demonstrated improved accuracy, scalability to large cancer studies, capability in handling both sequencing and SNP array data, and the potential to improve the estimation of tumor ploidy and purity.

## Author Summary

Somatic copy number alterations (SCNAs) are essential in oncogensis and progression of a variety of cancers. Accurate identification and quatification of SCNAs are fundamental in the effort of cataloging different variants in cancer genome. This task has its own challenges due to complex nature of tumor SCNA profile and is further complicated by the heterogeneity of the cells collected from a tumor tissue and the contamination from adjacent normal cells, making it difficult for the methods well tailored for the detection of germline copy number variation (CNV) to fit in tumor SCNA detection. Next generation sequencing provides an opportunity to comprehensively characterize SCNA at unprecedent resolution. While total read depth information is commonly used in SCNA detection methods, the allele-specific read depth is less often considered, leading to sub-optimal solution. By incorparating both pieces of information, we developed a segmentation-based pipeline to address aforementioned issues in SCNA detection. This tool is applicable on both deep sequencing data as well as SNP array data and enables accurate and efficient characterization of genome-wide SCNA profile to facilitate large-scale cancer studies.

## Introduction

Profound somatic copy number alternations (SCNAs) are present in many types of tumors [1–3], where they affect a larger fraction of the genome than other types of somatic variations [3,4]. The roles of SCNAs in promoting oncogenesis and tumor progression are under intensive study. In contrast to germline copy number variations (CNVs), which are sparsely distributed along the genome and of small to moderate size, tumor SCNAs are large in size and have a much wider range of magnitudes in copy number. Accurate detection and characterization of genome-wide SCNA profile are further complicated by aneuploidy and heterogeneity of tumor cells and contamination of normal cells [5].

Array comparative genomic hybridization (array-CGH) [6] and single nucleotide polymorphism (SNP) array [7] were widely used in surveying genome-wide SCNAs in the past decade. More recently, next-generation sequencing (NGS) technology provides unprecedented resolution to comprehensively characterize SCNAs at rapidly decreasing cost [8,9]. A long list of tools has been developed and successfully applied in analyzing CNV data harvested from NGS; see [2,10,11] for reviews. The available methods mainly fall into four categories: 1) read depth (RD), 2) pair-end mapping (PEM), 3) split read (SR) and 4) Assembling (AS). While they take advantage of complementary information, featuring different perspectives of CNVs, each of them encounters important limitations, due to the complications from the high dynamics of cancer genome. With whole genome sequencing (WGS) data, the latter three methods are good at detecting CNVs of small to moderate sizes, locating CNV break points at higher resolution, and discovering copy neutral rearrangements, but they are less capable in characterizing large-size and wide-range copy number changes at the genome-wide scale. Moreover, their applicability in whole exome sequencing (WES) data is limited by the fact that short reads from WES are concentrated in interspersed genomic regions. Therefore, the RD-based methods are used more widely in the study of tumor CNVs with both WGS and WES data [10]. Basically, the majority of RD-based methods, such as CNV-seq [12], SegSeq [9], ExomeCNV [13] and PatternCNV [14], follows a “bottom-up” procedure: short-reads mapping, normalization of read depth, copy number estimation in a local region (usually in a window of certain size or in an exonic region) and segmentation to merge regions with the same copy number status [10]. This strategy is sensitive in the detection of germline CNVs, but for tumor CNVs (i.e. SCNAs), it is difficult for the local inference to correctly decide the baseline of ploidy and accurately discern weak signals of copy number change in the presence of aneuploidy and normal cell contamination, so that the genome-wide inference drawn from the later segmentation step is inclined to accumulate false positive findings from the earlier local inference step. Further, these methods only consider the aggregated depth of sequencing reads carrying paternal and maternal alleles, aimed at estimating the total copy number, but largely ignore allele specific read depth, while the latter contains critical information of copy number change, copy-neutral loss of heterozygosity (CN-LOH), and, importantly, genome ploidy.

In analogy to Illumina SNP array [7], the allele specific signal from NGS can be quantified at heterozygote sites (e.g. SNP loci) and converted to the so-called B allele frequency (BAF), which is defined as the proportion of the reads carrying “B” allele (i.e. non-reference allele) among all the mappable reads at that site. At an SCNA-free locus, the expectation of BAF is close to 1/2, indicating equal amount of paternal and maternal alleles. With an SCNA event, the expectation of BAF might deviate from 1/2, reflecting differential copy number changes in the two alleles.

The two data dimensions, total read depth and allele-specific signal (i.e., BAF), carry complementary and consensual information of SCNA events. Incorporating both quantities could theoretically improve power and accuracy in identifying SCNA segment boundaries and characterizing alteration types (i.e., gains, losses, and CN-LOHs). Control-FREEC [15] is a representative method that incorporates both types of information. However, it uses them separately rather than jointly in the segmentation and SCNA calling, nor does it make good use of data from match normal sample to generate reliable allele-specific signal. Herein, we propose a tumor SCNA analysis method, SAAS-CNV, based on a joint segmentation algorithm [16], to accommodate both total read depth and BAF. Using both data dimensions simultaneously, the method first takes a “top-down” strategy to partition the genome into segments with different alterations by joint segmentation, and subsequently, determines their alteration types. The method was designed for paired normal-tumor settings, in which the spatial variability from non-uniform distribution of short reads along the genome can be alleviated [13]. It is able to accommodate WGS and WES data as well as SNP array data.

In this paper, we first characterized the type-I-error properties of SAAS-CNV using a carefully designed “null” dataset containing no SCNAs (H_0_), created by pairing sequencing replicates of a single HapMap sample as normal-tumor pairs [17] (Materials and Methods, Dataset I). Further, we demonstrated its ability to analyze a real-world WGS study of 88 hepatocellular carcinoma (HCC) samples (H_a_), some of which exhibit highly complicated SCNA profiles [18,19] (Materials and Methods, Dataset II). In both settings (H_o_ and H_a_), we compared the performance of SAAS-CNV with other existing methods. We also explored its potential extension in the estimation of ploidy and purity of tumor cells.

## Methods and Materials

### Data

#### Dataset I

The DNA sample of HapMap NA18507 was sequenced multiple times with different configurations of runs, machines and lanes, using Illumina HiSeq 2000/2500 [17]. 100bp paired-end reads were generated in both WGS and WES runs. For WES, exonic DNA was captured with the SeqCap EZ Human Exome Library v3.0 (NimbleGen). We obtained 6 WES replicates and 3 WGS replicates. Further we retrieved an additional WGS experiment of NA18507 from European Nucleotide Archive (ENA, accession: ERP001231). To mimic the tumor-normal pair study design, one replicate was treated as tumor and another as paired normal. Exhausting all paring possibility, a total of 15 pairs were synthesized on WES data and 6 pairs on WGS data, respectively. More information about the technical replicates and synthesized normal-tumor pair was summarized in S1–S4 Tables. Using technical replicates of the same sample ensures no SCNA exists and thus the dataset can serve as “null” scenario. Dataset I was used to calibrate the specificity of SCNA detection methods.

#### Dataset II

A total of 88 pairs of hepatocellular carcinoma (HCC) and adjacent normal liver genomes were sequenced with 100bp paired-end reads [18,19]. The WGS data was downloaded from ENA (accession: ERP001196). The read depth information was summarized in S1 Fig. To simulate WES scenario, we extracted genetic variant loci in exonic regions specified in SeqCap EZ Human Exome Library v3.0 (NimbleGen). On the same set of 88 tumor-normal pairs, Illumina HumanHap 650Y SNP array data [20] were retrieved from GEO (accession: GSE28127).

### Analysis pipeline

**Fig 1:**
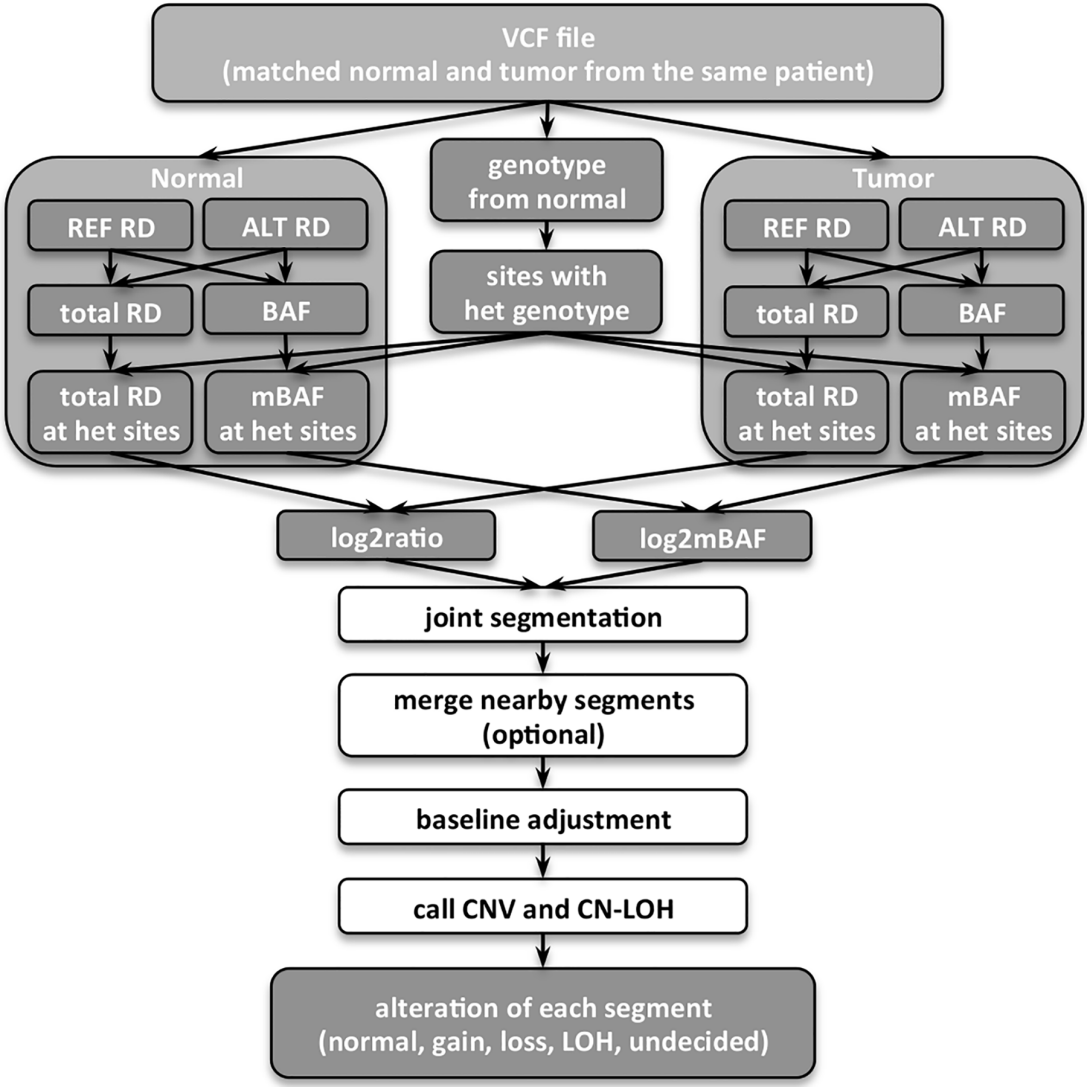
Flow chart of the SCNA analysis pipeline for matched normal and tumor sequencing data. REF: reference allele; ALT: alternative allele; RD: read depth; het: heterozygous.

We used GATK variant analysis pipeline [21–23] to process raw fastq files in this study [17] (more details in S1B Text). As a standard output of the pipeline, single nucleotide variants (SNVs) and small insertion and deletions (Indels), called from mapped high-quality reads, were saved in the variant call format (VCF) files [24]. At each variant locus, we extracted the genotype and the depth of reads carrying reference allele and alternative allele, all informative for SCNA inference. Fig 1 overviews the workflow of our algorithm in the paired normal-tumor setting.

#### Data transformation

For each tumor-normal pair, we only consider biallelic heterozygous loci observed in the normal tissue, and we refer the reference allele as “A” allele and the alternative (or non-reference) allele as “B” allele. To capture the SCNA signals derived from both the total read depth and BAF at each site, we define two quantities, termed log2ratio and log2mBAF, as follows:

$$\text{log}2\text{ratio}=\log_{2}\left(\frac{\text{total RD of tumor}}{\text{total RD of normal}}\right)=\log_{2}\left(\frac{A_{T}+B_{T}}{A_{N}+B_{N}}\right)$$ (1)

$$\text{log}2\text{mBAF}=\log_{2}\left(\frac{\text{mBAF of tumor}}{\text{mBAF of normal}}\right)=\log_{2}\left(\frac{f_{m}(B_{T}/(A_{T}+B_{T}))}{f_{m}(B_{N}/(A_{N}+B_{N}))}\right)$$ (2)

where *A*_*N*_ and *B*_*N*_ (*A*_*T*_ and *B*_*T*_) are the normalized read depths carrying A allele and B allele in normal (tumor) at each site by dividing the read depth by the overall depth of coverage of the normal (tumor) sample; mirrored BAF (mBAF) is obtained by the transformation: mBAF = *f*_*m*_(BAF) = |BAF − 0.5|+0.5, originally proposed for the CNV analysis of Illumina SNP array data [25]. The mBAF dissolves the arbitrary definition of B allele with respect to haplotype, so that a segmentation-based approach can be readily applicable to this signal. By taking log2 of the ratio of mBAF between tumor and normal instead of using tumor mBAF alone, the log2mBAF signal is more adhere to normal distribution (S2 Fig). This is a desired feature, because the joint segmentation algorithm we employed (see Joint segmentation section) assumes normality of the noise.

#### Joint segmentation

The genome-wide pattern of the two quantities (log2ratio and log2mBAF) is assumed to follow a piece-wise constant structure–they can be partitioned into consecutive segments with different means, reflecting different copy number status. Importantly, the change points of the two quantities are expected to be concordant, since they are derived from the same genomic alteration profile. To accommodate the two-dimensional SCNA readouts (log2ratio and log2mBAF) simultaneously, we employ an available joint segmentation algorithm [16]. This algorithm is a natural generalization of the circular binary segmentation (CBS) algorithm [26], extending CBS’s capability of segmenting one-dimensional signal to jointly segmenting multiple-dimensional signals. In our pipeline, it screens the two sequences of data points simultaneouly by a chi-square type of scan statistic in a recursive manner like CBS and results in a set of concordant change points in the two dimensions. The algorithm can approximate the significance level of the scan statistic in an analytic form, so that it has good computational efficiency. Afterwards, median values of each segment in the two dimensions are taken for the downstream SCNA inference.

#### Merging segments (optional)

If an excessive number of change points are reported by the segmentation step, a portion of them may be caused by noise and other spatial variability, but not related to actual copy number change. Thus, it is beneficial to reduce the number of artificial change points by merging adjacent segments, for which the median values in either or both dimensions are not substantially different. This may result in reduced false positives and better interpretability. For this purpose, we construct a *χ* ^2^-type statistic with two degrees of freedom:

$$\delta^{2}={\left(\frac{{\bar{x}}_{1}-{\bar{x}}_{2}}{{\hat{\sigma }}_{x}\sqrt{1/n_{1}+1/n_{2}}}\right)}^{2}+{\left(\frac{{\bar{y}}_{1}-{\bar{y}}_{2}}{{\hat{\sigma }}_{y}\sqrt{1/n_{1}+1/n_{2}}}\right)}^{2}$$ (3)

where *n*_1_ and *n*_2_ are the numbers of loci spanning the two adjacent segments; ${\bar{x}}_{1}$ and ${\bar{x}}_{2}$ the median values of log2ratio, and ${\bar{y}}_{1}$ and ${\bar{y}}_{2}$ the median values of log2mBAF; ${\hat{\sigma }}_{x}$ and ${\hat{\sigma }}_{y}$ are robust estimates of the noise level of the two signals (S1A Text). Instead of assuming a *χ* ^2^-distribution to test the significance of *δ* ^2^ away from 0, we employ a resampling strategy. The loci within “normal” segments, which are identified by the same procedure as described in the baseline adjustment section, are pooled together by concatenating them to establish the “null” data. From the “null” data, a set of two adjacent segments, with matched sizes as the two observed segments, is randomly drawn and the *δ* ^2^ statistic is calculated for each random sample respectively to establish the null distribution. The nominal p-value is computed as the proportion of the randomly drawn samples with larger *δ* ^2^ statistic than the observed one. Two adjacent segments with p-value greater than a pre-specified cut-off (e.g. 0.05) are merged and this procedure is done recursively until no segments within a chromosome can be merged. In this study, we used 1000 random samples to establish the null distribution. Please refer to S1C Text for more details.

#### Baseline adjustment

**Fig 2:**
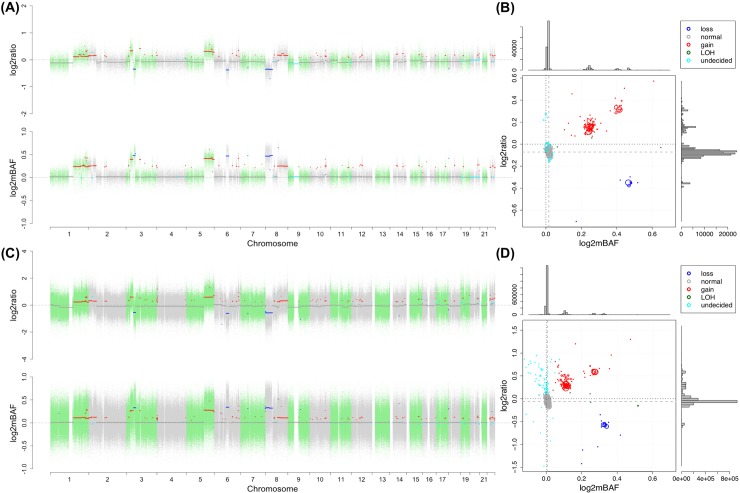
SCNA profile for the sample PT017 from Dataset II. (A) and (B) display SNP array data and (C) and (D) WGS data. In (A) and (C), on the top panel, the log2ratio signal is plotted against chromosomal position and on the bottom panel, the log2mBAF signal. The dots, each representing a locus, are colored alternately to distinguish chromosomes. The segments, each representing a DNA segment resulting from the joint segmentation, are colored based on inferred SCNA status. In (B) and (D), on the main log2mBAF-log2ratio panel, each circle corresponds to a segment in (A) and (C), with the size reflecting the length of the segment; the color code is specified in legend; the dashed gray lines indicate the adjusted baselines. The side panels, corresponding to log2ratio and log2mBAF dimension respectively, show the distribution of median values of each segment.

The accurate quantification of SCNA is complicated by normal cell contamination and tumor cell aneuploidy and heterogeneity, which could distort the baseline of both log2ratio and log2mBAF signals, corresponding to unaltered genomic segments. Therefore, it is critical to corretly identify the baseline, based on which the SCNA state is assigned to each segment. For this purpose, we used a heuristic searching strategy sequentially in log2mBAF and log2ratio dimensions (Fig 2B and 2D). In log2mBAF dimension, we initialize a search of “normal” segments within a restricted window surrounding 0, [−*w*,*w*], in favor of segments with balanced alleles, where *w* is empirically chosen as $\rho {\hat{\sigma }}_{y}$ (*ρ* = 0.5 in this study). Then we take as the log2mBAF baseline the mode of segment medians within the window, weighted by the size of segment, i.e., the median of a segment with n loci is counted n times. The segments within the *d* radius of the cluster centered at the identified log2mBAF baseline are assigned “normal” status, where *d* can be chosen as 2*η* or 3*η*. Here, *η* can be the estimated standard deviation from segments falling in the window or simply $r{\hat{\sigma }}_{y}$ (*r* = 0.3 in this study), which is more stable based on our experiences in analyzing several real datasets. If no segments can be found in the initial window, we just enlarge the window size *w* as $2\rho {\hat{\sigma }}_{y}$, $3\rho {\hat{\sigma }}_{y}$ and so on until we can find segments falling in the window. In log2ratio dimension, the mode of segment medians (weighted by segment size) belonging to the cluster of “normal” segments, identified in the log2mBAF dimension, is regarded as the log2raio baseline. This searching strategy works well in the case that a considerable portion of the genome presents balanced alleles (e.g. diploidy or tetraploidy). In the more complicated case where almost the whole genome is altered in an unbalance way, the baseline may need to be adjusted manually. For the fairness in method comparison, we used automatically identified baseline in this study. We provide a feature in the saasCNV package (see discussion section) that facilitates the users to manually adjust the baseline according to diagnosis plots like Fig 2 when necessary.

#### SCNA calling

After baseline adjustment, our method assigns the alteration state to each segment. The possible states include gain, loss, CN-LOH and normal. The gain and loss refer to the total copy number change with respect to the adjusted baseline, while they may or may not present allelic imbalance at the same time. For example, if the baseline copy number (CN) is 2 (Fig 2), CN 0 and 1 are called as loss and CN>2 as gain; if the genome experienced a whole-genome doubling event and exhibits tetraploidy (see results section), the baseline CN becomes 4 and CN<4 is called as loss and CN>4 as gain. CN-LOH indicates loss of one allele, leading to allelic imbalance, while the total copy number remains unchanged from baseline.

In SCNA calling, we again adopt the resampling strategy to establish null distribution based on signals from those “normal” segments that are identified in baseline adjustment step. More precisely, we propose three statistics to quantify the alterations in both log2ratio and log2mBAF dimensions from different perspectives:

$$\delta_{xy}^{2}={\left(\frac{\bar{x}}{{\hat{\sigma }}_{x}/\sqrt{n}}\right)}^{2}+{\left(\frac{\bar{y}}{{\hat{\sigma }}_{y}/\sqrt{n}}\right)}^{2},\;\delta_{x}^{2}={\left(\frac{\bar{x}}{{\hat{\sigma }}_{x}/\sqrt{n}}\right)}^{2},\;\delta_{y}^{2}={\left(\frac{\bar{y}}{{\hat{\sigma }}_{y}/\sqrt{n}}\right)}^{2}$$ (4)

The notations are interpreted in the similar way as in Eq (3). A set of segments with the same length as the segment under consideration is drawn from the pool of loci located in all those “normal” segments. The three statistics are calculated repeatedly for the randomly drawn samples and pooled to form the null distribution for each statistic respectively. The nominal p-values *p*_*xy*_, *p*_*x*_, and *p*_*y*_ are then computed as the proportion of randomly drawn values greater than observed values for each statistic. We used 1000 random samples to establish the null distribution in this study. The alteration status is decided with a set of criteria and a pre-specified significance cut-off *θ* (e.g. 0.05) as listed in Table 1.

**Table 1:** Criteria for alteration status calling.

Alteration	log2ratio	log2mBAF	Both dimension
normal^a^	*p*_*x*_ > *θ* ^c^	*p*_*y*_ > *θ*	*p*_*xy*_ > *θ*
gain (imbalanced)	*p*_*x*_ < *θ* and $\bar{x}>0$	*p*_*y*_ < *θ* and $\bar{y}>0$	*p*_*xy*_ < *θ*
gain (balanced)^b^	*p*_*x*_ < *θ* and $\bar{x}>u{\hat{\sigma }}_{x}$ ^d^	*p*_*y*_ > *θ* and $\bar{y}>0$	*p*_*xy*_ < *θ*
loss (imbalanced)	*p*_*x*_ < *θ* and $\bar{x}<0$	*p*_*y*_ < *θ* and $\bar{y}>0$	*p*_*xy*_ < *θ*
loss (balanced) ^b^	*p*_*x*_ < *θ* and $\bar{x}<-u{\hat{\sigma }}_{x}$ ^d^	*p*_*y*_ > *θ* and $\bar{y}>0$	*p*_*xy*_ < *θ*
CN-LOH	*p*_*x*_ > *θ*	*p*_*y*_ < *θ* and $\bar{y}>0$	*p*_*xy*_ < *θ*
undecided	otherwise		

^a^ The segments initially assigned of normal status in the baseline adjustment step also undergo the SCNA calling step, which is intent to refine the inference from the initial heuristic search.

^b^ Balanced gains and losses are much less common than imbalanced gains and losses. See S1D Text for more details.

^c^ *θ* = 0.05 in this study unless specifically noted otherwise.

^d^ *u* = 1.5 in this study.

We acknowledge that the definition may miss some rare cases due to the complexity of cancer genome. A further goal beyond calling SCNA status qualitatively is to provide an estimate of absolute copy number for each segment with normal cell contamination and tumor heterogeneity taken into account. In the Results section, we show an example where incorporating BAF substantially improved the copy number inference from total read depth alone. We will approach this goal in future works.

#### Application to SNP array data

SNP array generates counterparts of the two dimensions of information we derived from NGS, therefore SAAS-CNV can be readily applied to SNP array data. For example, Illumina SNP array generates two quantities, log R ratio (LRR) and B allele frequency (BAF)[7]. LRR reflects total copy number and can be directly taken as log2ratio, while BAF is defined in the same way as for NGS data, except that the signals from the alleles are measured by fluorescent intensities with SNP array, instead of allele specific read depths with NGS. Following the same transformation in Eq (2), log2mBAF can be derived from tumor and normal BAF values. Shown in Results section, SAAS-CNV was applied to Illumina SNP array data of HCC tumor-normal tissue pairs (e.g., Fig 2A).

### Data analysis

For both datasets, a standard implementation of NGS analysis pipeline following the GATK best practices for variant detection [17,21,22] was applied to the raw FASTQ files to generate recalibrated and deduplicated high-quality BAM files [27], as well as VCF files containing detected SNVs and Indels. Information retrieved from biallelic heterozygous sites were processed by our SCNA analysis pipeline (Fig 1). Illumina SNP array data was processed to generate log2ratio and log2mBAF signals in a similar way for downstream analysis. Detailed data analysis steps are described in S1B Text.

#### Dataset I

We applied SAAS-CNV to analyze both WES and WGS data in Dataset I. On WES data, we compared our method with ExomeCNV [13] and PatternCNV [14]. ExomeCNV was designed specifically for matched tumor-normal WES data, taking total read depth from BAM files for copy number inference and BAF information from VCF files for CN-LOH inference. Instead of using matched normal, PatternCNV [14] was recently developed to take a group of normal samples as reference to acount for both spatial average and variance patterns of read depth. For PatternCNV, every NA18507 replicate was treated as tumor in turn, and constrasted with the rest five replicates as normal.

On WGS data, we compared our method with CNAnorm [5] and Control-FREEC [15]. CNAnorm was developed for the tumor-normal setting and capable of correcting for normal cell contamination and accounting for tumor aneuploidy. It retrieves total read depth information from BAM files as input. Control-FREEC can utilize both total read depth and BAF for CNV and CN-LOH inference. It extracted the two types of information from pileup format files [27].

#### Dataset II

We applied six different methods on three different data platforms in Dataset II: SAAS-CNV on WGS, synthesized WES and SNP array, CNAnorm, Control-FREEC and CREST [28] on WGS, ExomeCNV on synthesized WES, and GAP [29] on SNP array. We compared these methods from the following three perspectives.

#### 1) Accuracy in SCNA detection

**Fig 3:**
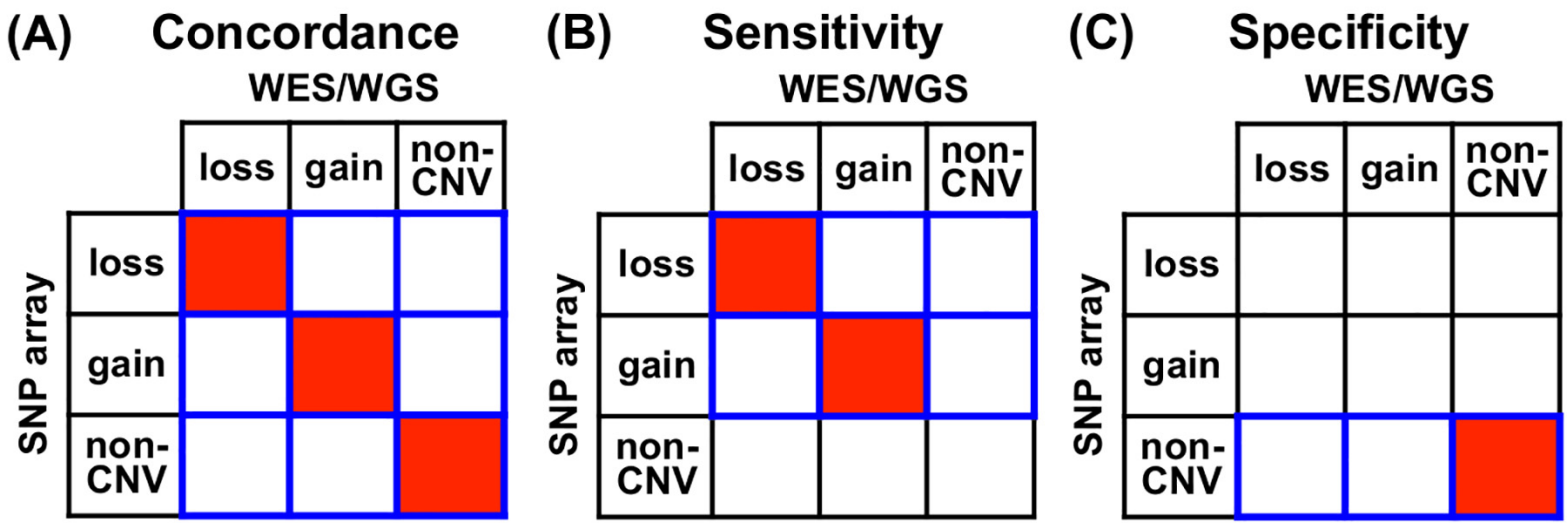
Definition of different accuracy indices. Each of the indices is defined as the ratio of the total count in red cells to the total count in blue-border cells. (A) Concordance. (B) Sensitivity. (C) Specificity.

For this comparison, we used the results from Genome Alteration Print (GAP) [29] on SNP array data to serve as benchmark. GAP uses both total copy number and BAF information for SCNA inference, and it is able to estimate tumor purity and automatically decide the baseline copy number in tumors with complex SCNA profile when normal cell contamination exists. It also provides intuitive visualization method that allows users to visually check the results and manually correct them when necessary. We manually reviewed and validated all the GAP results. We chose SNP array platform as benchmark for at least three reasons: 1) A majority of SCNAs is large and can be captured by high density SNP array (see Results section). A well-designed method for sequencing platfrom is expected to perform well on large SCNAs. 2) SNP array presents better signal-to-noise ratio than sequencing with moderate coverage (see Results section). 3) SNP array has been and is still widely used in large-scale cancer studies. Since CNAnorm only calls copy number loss and gain, all other SCNA states (e.g. normal, CN-LOH) were collapsed down to one category, named “non-CNV”. The unit for comparison is segment rather than individual locus. We define the overlap rate of two CNV segments, detected respectively from two different analyses for the same sample, as the ratio of the length of the intersection of the two segments to the length of their union. The two segments are called consensus if their overlap rate passes a pre-determined threshold (e.g. 50%). We picked all the consensus segments from the two analyses under comparison and counted the number of the segments falling into each category (cell) of the contingency tables in Fig 3. We define concordance, sensitivity and specificity as the ratio of the total count in red cells to the total count in blue-border cells, respectively. Receiver operating characteristic (ROC) curves were generated by varying p-value threshold (Table 1) for SAAS-CNV, by varying copy number threshold for CNAnorm and Control-FREEC, and by varying segment log2ratio threshold for ExomeCNV.

#### 2) Accuracy in identification of change points

For this comparison, we used the results from CREST [28] applied to WGS data as benchmark. CREST utilizes soft-clipped reads, a type of information orthogonal to sequencing depth, for structural variant (SV) calling. The change points can be detected up to single base pair resolution. We regarded those change points supported by at least five soft-clipped reads as real ones and used them as reference. We checked whether the change points, which were actually boundaries of neighoring segments produced by segmentation from different methods, were consistent with the reference. A change point found by CREST is considered as detected by a segmentation-based method if there exist two neighboring segments such that the CREST change point falls in the gap between the two segments (i.e., between the right boundary of the 5’ segment and the left boundary of the 3’ segment).

#### 3) Computational time

We ran all the tools on a Linux high performance cluster with AMD 2.3GHz CPUs. The computational time was unified into single thread CPU time.

## Results

### An illustration of the data and method

First, we present a visualization of the processed signals and the results from SAAS-CNV. Fig 2 demonstrates a typical example taken from the analysis of Dataset II. The 2-dimensional profiles (log2ratio and log2mBAF) from SNP array (Fig 2A and 2B) and WGS (Fig 2C and 2D) were processed for genome-wide SCNA detection. The signals in the two dimensions are altered by copy number gains and losses, showing consistent patterns of changes, for both platforms. The alteration status assigned to each segment is highly consistent between the two platforms (Fig 2A and 2C). We projected the medians of log2ratio and log2mBAF of each segment onto a 2-D space for SNP array and WGS respectively, confirming the consistency of the inference drawn from the two platforms (Fig 2B and 2D). Segments with the same alteration status are clustered together. Segments with less difference in log2ratio dimension may be distinguished in log2mBAF dimension, suggesting the valuable information added from BAF for SCNA inference. It is noticed that the original baselines in both dimensions deviate substantially from zero (Fig 2B and 2D), and simply using zero as baseline for SCNA inference would result in many false positive calls, underscoring the necessity of baseline adjustment.

### Analysis of Dataset I (where H_o_ is true)

#### Analysis of NA18507 WES data

Processed with the GATK pipeline [17], the NA18507 WES data shows high depth of coverage, ranging from 75.5x to 110.1x (S1 Table). The average read depths at heterozygous loci are about 25% smaller, ranging from 57.6x to 79.9x (S1 Table), because only mappable reads passing certain quality control criteria were used for SNV and Indel calling.

**Fig 4:**
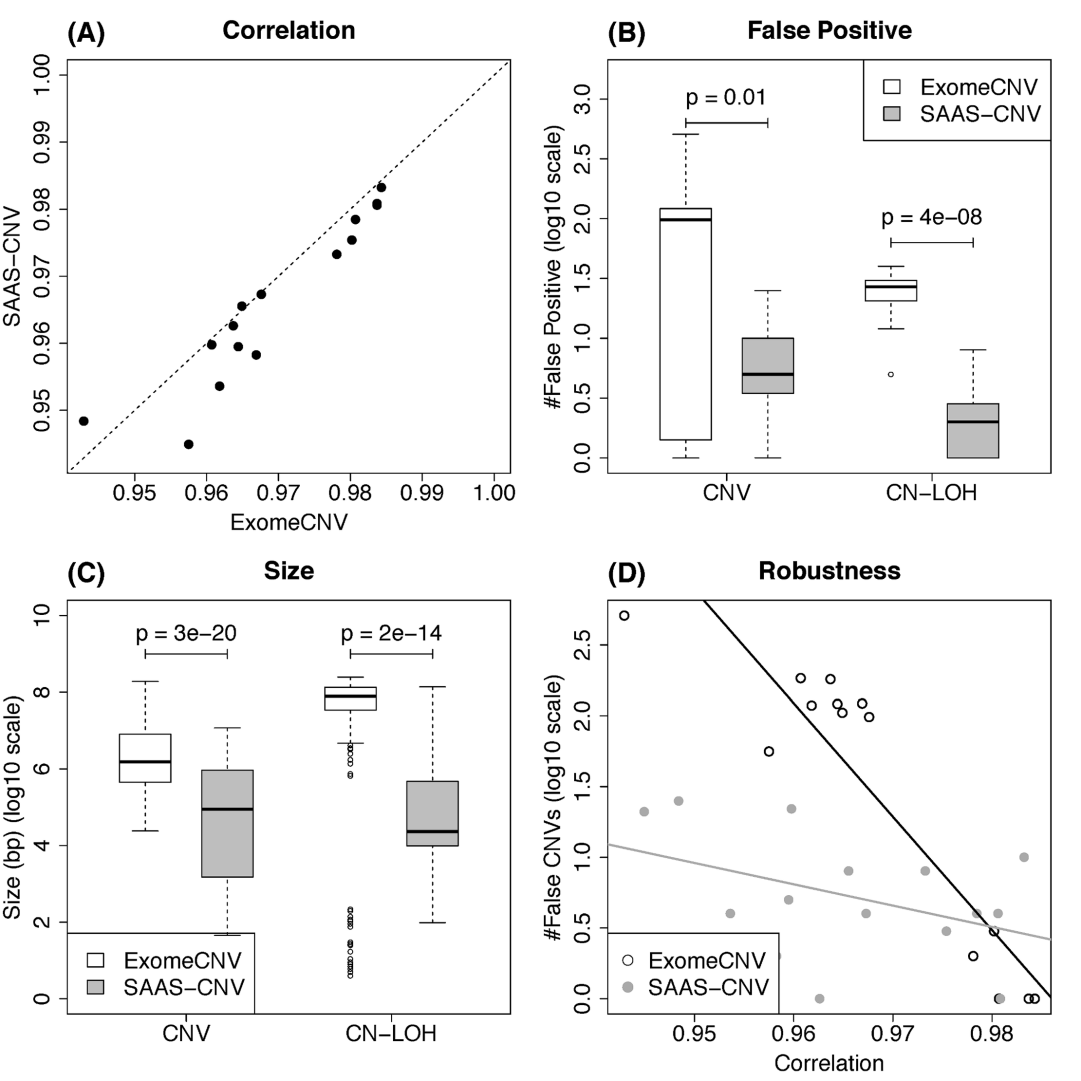
Comparison of results from ExomeCNV and SAAS-CNV on NA18507 WES data. (A) Correlation of total read depths between each synthesized tumor-normal pair for ExomeCNV versus SAAS-CNV. (B) The number of false positives for CNV and CN-LOH. (C) The size of false CNV and CN-LOH calls. (D) The number of false CNV calls versus the correlation. In (A) and (D), each dot represents a synthesized pair. In (A), the dashed line indicates the line with slope 1. In (D), the black line indicates the fitted linear regression line for ExomeCNV and gray line for SAAS-CNV. In (B) and (C), p-values are based on paired t-test.

To check the data quality, we investigated the correlation of total read depths between normal and tumor data of each synthesized pair across heterozygous sites in the analysis with SAAS-CNV and across exonic regions (exome) in the analysis with ExomeCNV (S1J Text). In NGS data, the non-uniform variability in total read depth is commonly observed. It is unrelated to copy number change but due to non-uniform distribution of GC-content, differential sequencing efficiency in different genomic regions, and other artifacts. This variability is more pronounced in WES, since the exonic DNA sequences are not captured equivalently. This issue could affect SCNA detection and result in false positives. Fortunately, in the normal-tumor pair study design, the undesired variability can be neutralized by contrasting read depth from tumor and paired normal samples [13]. A large correlation coefficient implies that the artificial variability is spatially correlated across the genome between the two samples and thus is likely to be remedied (also see S1L Text). For tumor-normal pairs synthesized by sequencing replicates (Materials and Methods, Dataset I), the correlation is very large (Fig 4A and S3 Table). We also noticed that the strength of correlations in the two analyses are comparable, although the read depth was summarized in different ways (Fig 4A).

Next, we compared the CNVs and CN-LOHs called by the two methods. They are false positives in the context of our experimental design. ExomeCNV detected significantly more false positives than SAAS-CNV (Fig 4B), although the parameters controlling specificity was set more stringent than default values (S1E Text). The sizes of detected CNVs and CN-LOHs by ExomeCNV are also orders of magnitude larger than those by SAAS-CNV (Fig 4C). With a closer inspection on the intermediate results from the bottom-up merging step of ExomeCNV (S1E Text), some large segments were called CNVs/CN-LOHs merely because they spanned several exons that were called as CNVs/CN-LOHs at single exon level. This is an intrinsic drawback of the bottom-up strategy. For SAAS-CNV, we noticed that 36% falsely detected CNVs/CN-LOHs span 10 heterozygous loci, which is the pre-defined lower bound of segment size (S3A Fig). We also found that the average read depths in the detected 10-locus regions are smaller than those of randomly picked 10-locus regions (S3B Fig). Therefore, a large portion of SAAS-CNV false positives is attribute to low coverage in some exonic regions, a limitation of WES platform.

Further, we investigated the robustness of the methods with respect to data variability. For both methods, we observed that the number of false positives increases as the correlation of total read depth between the paired sequencing replicates decreases (Fig 4D). Such impact is more pronounced in ExomeCNV performance (note the y-axis in Fig 4D is on log10 scale).

Moreover, to check whether incorporating log2mBAF helps enhance the specificity and robustness, we compared the analysis using log2ratio alone to the method jointly using both log2ratio and log2mBAF. The joint method produced less false positives and was more robust to the signal variability (S4 Fig).

Finally, we compared SAAS-CNV with PatternCNV [14]. PatternCNV calls CNVs from a tumor sample by contrasting it with a collection of multiple normal samples as reference instead of one matched normal sample. PatternCNV is different from other read depth based methods in that the exon-wise read depth was calculated by weighted average of read depths of multiple bins within the exon, where the weight are inversely proportional to the variance of bin-wise read depth estimated from the multiple normal samples. Though the noise level is expected to be further reduced by taking advantage of more reference samples, we still observed more false postives incurred from PatternCNV than SAAS-CNV (see S1F Text, S5 Fig and S6 Fig for more details). PatternCNV provides CNV calls at exon level but does not aggregate exon-wise CNV calls to segment level, although it provides an option of segmenting log2ratio signal by CBS [26] merely for visualization purpose. Therefore, it is difficult to interpret the PatternCNV results in real data analysis. Since the comparison of all other methods based on Dataset II was at segment level, we did not apply PatternCNV to Dataset II (S1F Text).

In summary, SAAS-CNV benefits from the top-down segmentation strategy and allele specific information in terms of less false positives and better robustness to noisy data as compared with ExomeCNV and PatternCNV, which utilized total read depth information alone. We factor two main reasons into its good performance: 1) With long-range large segments rather than local small segments, especially with the aid of more stable log2mBAF signal, SAAS-CNV can anchor the global baseline more easily, so that incorrect SCNA calls for large segment can be avoided (S5 Fig); 2) in SCNA calling step, it prevented a majority of outliers in log2ratio from being called as SCNAs with the evidence in log2mBAF (S5C Fig).

#### Analysis of NA18507 WGS data

On WGS data, we investigated similar metrics as in WES study. SAAS-CNV was compared to CNAnorm [5] and Control-FREEC [15]. CNAnorm and Control-FREEC retrieved mappable reads from BAM files and took 1kb non-overlapping window as basic unit (locus) within which the average total read depth was calculated. The processed WGS data show moderate depth, ranging from 33.8x to 61.9x (S2 Table). The average read depth across heterozygous sites for SAAS-CNV was comparable with the average read depth across all 1kb windows for CNAnorm and Control-FREEC (S4 Table).

**Fig 5:**
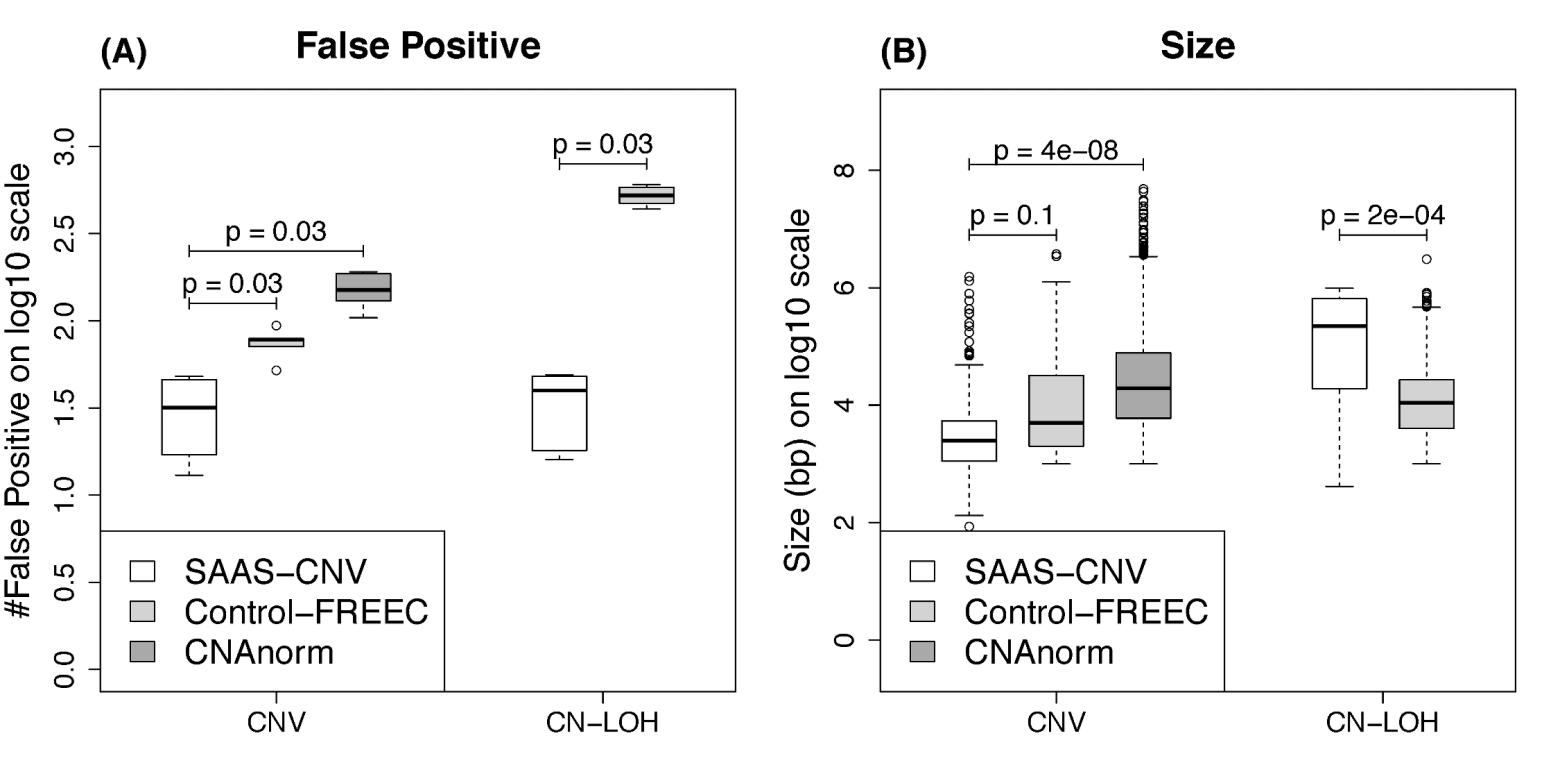
NA18507 WGS analysis results from SAAS-CNV, Control-FREEC and CNAnorm. The number (A) and the size (B) of falsely called alterations are compared between the three methods stratified by CNVs and CN-LOHs. CNAnorm only detects copy number gain and loss but not CN-LOHs. In (A), the p-values are based on paired Wilcoxon signed-rank test and have the same value due to the nature of rank-based test. In (B), the p-value is from two-sample t-test on the sets of falsely called CNVs (or CN-LOHs) pooled across the 6 pairs for each method respectively.

The Pearson correlation coefficients of read depths between pairs of replicated samples were calculated across heterozygous sites for SAAS-CNV and across 1kb windows for CNAnorm and Control-FREEC (S1J Text). The former is substantially smaller than the latter, although windows with extremely large read depth were excluded from calculation of correlation (S1J Text and S4 Table). This is not beyond expectation because more short reads were involved within 1kb intervals than at single heterozygous sites, leading to more consistent read depth profile between replicates. Nevertheless, SAAS-CNV still called less false positive CNVs than CNAnorm and Control-FREEC (Fig 5A), and the false CNVs are smaller in size than CNAnorm or Control-FREEC (Fig 5B). Control-FREEC produced hundreds more of smaller false CN-LOH calls than SAAS-CNV (Fig 5A and 5B). The main reason could be that Control-FREEC tried to exclude homozygous loci by applying a binomial distribution model with fixed probability of sequencing error on allelic specific read depth in tumor [15] rather than taking advantage of the actual genotype inferred from matched normal, resulting in many small blocks of putative heterozygous loci, which are in fact homozygous loci.

Similar to the WES study, a large portion of the false positive SCNAs (70.1%) detected by SAAS-CNV span 10 heterozygous loci, which is again the smallest possible size set by our threshold (S7A Fig). This can also be explained by the low depth of coverage in these 10-locus regions (S7B Fig). From S8 Fig, we can clearly see the advantage of the joint analysis over the analysis based on log2ratio alone for WGS data.

### Analysis of Dataset II (where H_a_ is true)

We processed the HKU HCC WGS data, consisting of 88 pairs of tumor and adjacent normal tissues, with the standard implementation of GATK pipeline [17]. The average read depths of 86 pairs are moderate, ranging from 24.8x to 71.5x, with two pairs sequenced at higher depth (>120x). The average effective read depths measured at heterozygous sites range from 18.2x to 99.8x, composing 68%~90% of available read depth (S1 Fig). We applied SAAS-CNV, CNVnorm and Control-FREEC on the 88 pairs of normal-HCC samples. Further, we synthesized WES data by retrieving reads located in exonic regions from WGS data. The synthesized WES data was used to test the performance of SAAS-CNV and ExomeCNV. We also applied SAAS-CNV and GAP on the SNP array data from the same samples. As stated above, the GAP results on SNP array data were used as “truth” in benchmarking other SCNA methods.

**Fig 6:**
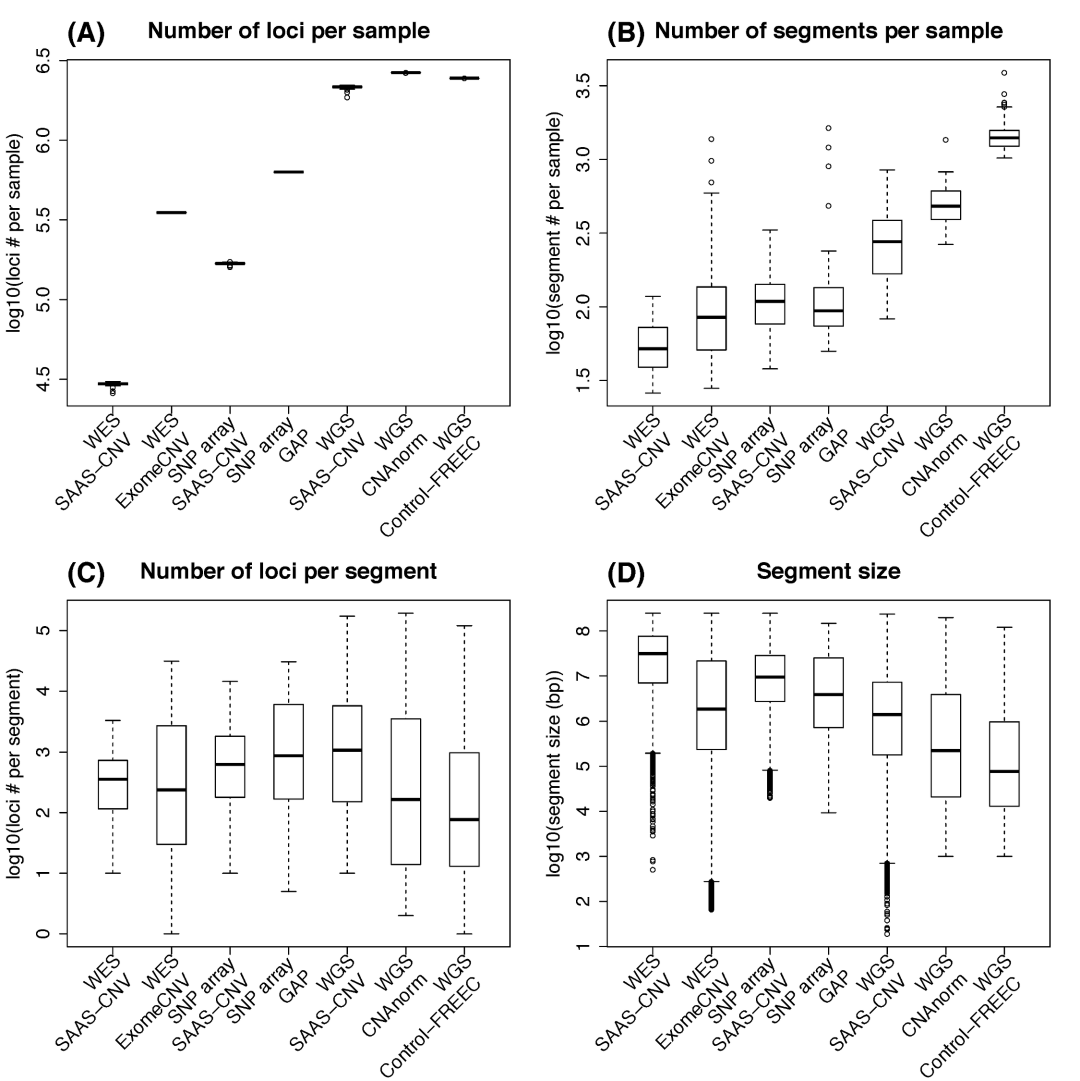
Summary of SCNA results for Dataset II. SAAS-CNV and ExomeCNV were applied on synthesized WES data, SAAS-CNV and GAP on SNP array data, and SAAS-CNV, CNAnorm and Control-FREEC on WGS data. (A) Number of loci on autosome per sample. (B) Number of segments per sample. (C) Number of loci involved in each segment. (D) The size (in bp) of each segment. In each sub-plot, y-axis is displayed on log10 scale.

Some basic metrics from these analyses are summarized in Fig 6. WGS provides millions of data points (loci) per sample while SNP array and WES provides hundreds of thousands of data points (loci) (Fig 6A). Since SAAS-CNV takes heterozygous loci as input, it utilizes less number of loci than competing methods. For example, only tens of thousands of loci were used by SAAS-SNV in WES data. On SNP array data, SAAS-CNV and GAP produced comparable number of segments per sample (Fig 6B), as well as comparable segment size in terms of locus number per segment (Fig 6C) and physical length (Fig 6D). On WES and WGS data, the competing methods tended to chop the genome into smaller segments (Fig 6B) than SAAS-CNV (Fig 6C and 6D).

#### Accuracy in SCNA detection

We used GAP results on SNP array data as “truth” in comparison. To make sure GAP correctly set the baseline and detected different types of alterations, we visually checked all the results from GAP for each sample with its visualization tools. GAP did not perform satisfactorily on four samples due to large noise of the data. Further, manual correction is not possible for these four sample, so we exclueded them from downstream analysis. We manually adjusted the GAP results of six out of the remaining 84 samples, where either the baseline or the tumor purity was not correctly estimated.

**Fig 7:**
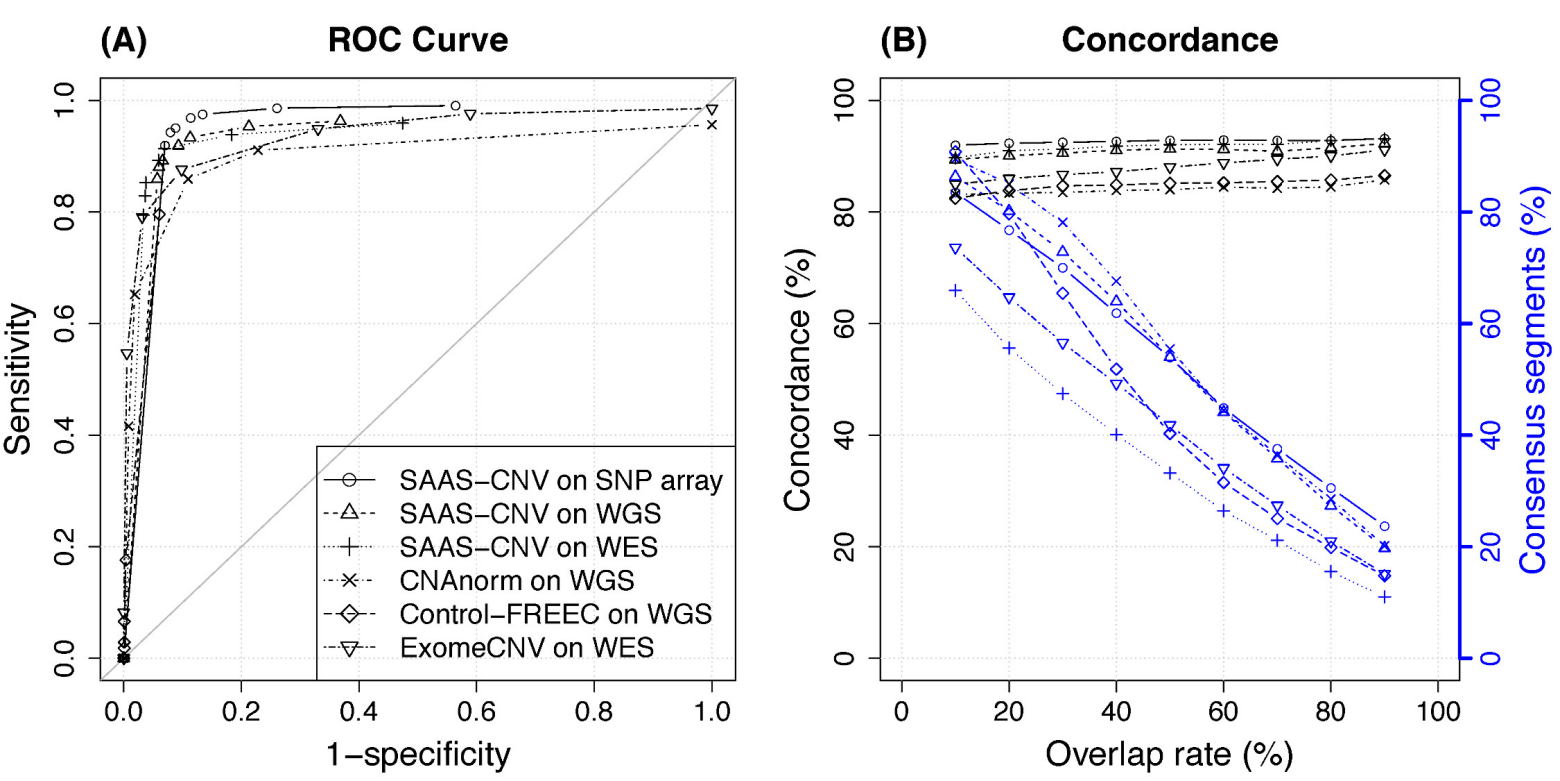
Comparison of results from six analyses on Dataset II. The results from GAP analysis on SNP array data are treated as benchmark. (A) ROC curves for different analyses coded in different lines. The overlap rate threshold is 50%. (B) The concordence of SCNA calls (black) and the percentage of consensus segments (blue) for the six analyses (coded the same way as in (A)), as the overlap rate threshold varies.

At the overlap rate threshold of 50%, we measured the ROC curves for different analyses (Fig 7A). We noted that the log2mBAF criterion (Table 1) prevented SAAS-CNV from calling some segments as gain/loss even if the p-value threshold was set to 1, so the SAAS-CNV results never reached 0 specificity in ROC curve. Control-FREEC provided integer copy number estimation for each segment and its ROC curve can not reach 0 specificity either, because the baseline copy number could never be called as gain or loss even though the threshold was reduced to zero copy. It is not surprising that SAAS-CNV results on SNP array data were the best among all the analyses, given the results were derived from the same platform as the “truth” (GAP results on SNP array data). The SAAS-CNV analysis on WGS and WES data resulted in favorable performance as compared to competing methods for different platforms respectively. As the overlap rate varies from 10% to 90% (S9 Fig), the relative performance of these methods remains similar to what appears in Fig 7A. Interestingly, WES only covers about 1% of the loci of WGS (Fig 6A) in SAAS-CNV analysis, but the result from WES is almost comparable with that of WGS. However, we should be cautious with the performance of SAAS-CNV and ExomeCNV on WES in this case, because real WES data may have larger noise than the synthesized WES data due to the target capture procedure.

In Fig 7B, as the overlap rate threshold increases, the percentage of segments passing consensus criterion (see methods section) decreases for all the analyses (blue lines), with the SAAS-CNV analysis on WES being the lowest mainly due to much lower locus density (Fig 6A). With more stringent overlap criterion (e.g., >40%), the percentage of consensus segments produced by SAAS-CNV on WGS and SNP array and CNAnorm are roughly the same and higher than Control-FREEC and ExomeCNV. Control-FREEC on WGS provides as many consenus segments as ExomeCNV on WES, suggesting its poor performance in segmentation, provided that WGS has much higher resolution than WES. As another accuracy measurement, the concordance of segment SCNA status remains stable as the overlap threshold varies (black lines). SAAS-CNV also performs better than competing methods. In summary, we demonstrated with a large empirical dataset that SAAS-CNV, by efficiently using both total and allelic specific read depth information in segmentation and SCNA calling, outperformed competing methods on each platform, for which these methods were specifically developed.

Analyzing empirical data (Dataset II) also provides guidance on the choice of p-value threshold (Table 1) for SAAS-CNV. Based on the ROC curves (Fig 7A), when the p-value threshold was at 0.001, 0.005, 0.01, and 0.05 (corresponding the second through the fifth points counting from bottom left corner along the curves), the sensitivity and specificity were rather stable (both around 90%). More stringent p-value threshold led to higher specificity and lower sensitivity and vice versa. In practice, p-value threshold is recommended in the range from 0.001 to 0.05, depending on the users’ preference for sensitivity and/or specificity.

#### Accuracy in SCNA change point detection

A total of 1518 genomic structure variant (SV) change points in 58 tumors was identified by CREST, where only the change points with evidence from at least 5 soft-clipped reads were considered as real ones. We investigated the SCNA detection methods’ abilities in accurately detecting the SV change point (Table 2). For WGS, SAAS-CNV correctly identified more change points than CNAnorm and Control-FREEC at comparable resolution. Considering CNAnorm and Control-FREEC partitioned the genome into much more segments than SAAS-CNV, the accuray of SAAS-CNV was considerably better than competitors. On SNP array data, SAAS-CNV again correctly identified more change points than GAP. On WES data, SAAS-CNV did not perform as well as ExomeCNV in change point detection mainly due to the limited number of heterozygous loci within exonic regions.

**Table 2:** Accuracy in change point detection.

Method	Platform	CREST change point detected^a^	#segments per sample^b^	Distance (kb)^c^	Locus density (#loci/Mb)^d^
GAP	SNP array	16.9%	97 (44)	3.7 (3.2)	219
SAAS-CNV	SNP array	23.9%	110 (46)	17.0 (19.6)	58
SAAS-CNV	WES	9.4%	57 (25)	240.7 (290.5)	10
ExomeCNV	WES	19.6%	93 (65)	29.5 (40.2)	122
SAAS-CNV	WGS	28.3%	289 (163)	1.2 (1.4)	751
CNAnorm	WGS	24.9%	499 (153)	0.5 (-)^e^	921
Control-FREEC	WGS	18.7%	1392 (290)	0.5 (-)^e^	851

^a^ A change point found by CREST at single base pair resolution is considered as detected by another method if there exist two neighboring segments produced by the method such that the change point falls in the gap between the two segments (i.e., between the right boundary of the 5’ segment and the left boundary of the 3’ segment). Display is the percentage of CREST change points detected by the method under consideration.

^b^ The median of per-sample segment number across the samples with CREST-identified change points. Median absolute deviation (MAD) is included in the parentheses.

^c^ The median half size of the gap (measured in kilo base pair, kb) between the segment boundaries harboring a CREST change point. Median absolute deviation (MAD) is included in the parentheses.

^d^ Locus density is defined as the number of loci distributed per million base pair (Mb) on average.

^e^ Since both CNAnorm and Control-FREEC were applied at the resolution of 1kb window, the half size of the gap harboring a CREST change point is exactly 0.5kb.

#### Computational time

Table 3 lists computational time for different methods in the analysis of 88 tumor-normal pairs in Dataset II. On SNP array data, our method took slightly longer time than GAP, although both methods were quite fast. On WES and WGS data, our method showed clear advantage over competing methods, making it scalable to large cancer sequencing study.

**Table 3:** Computational time for different methods applied to Dataset II.

Method	Platform	Input file	Coding language	Data processing time (min) ^e^	Analysis time (min) ^e^	Total time (min) ^e^
GAP	SNP array	LRR and BAF data^a^	R	0.8 (0.03)	4.2 (0.6)	5.0 (0.6)
SAAS-CNV	SNP array	LRR and BAF data^a^	R	0.6 (0.1)	9.8 (2.3)	10.4 (2.3)
SAAS-CNV	WES	VCF	R	0.1 (0.01)	1.3 (0.3)	1.5 (0.3)
ExomeCNV	WES	BAM (and VCF) ^b^	R	92.4 (26.6)	997.9 (206.7)	1090.3 (209.0)
SAAS-CNV	WGS	VCF	R	17.5 (4.1)	68.3 (26.7)	85.8 (27.2)
CNAnorm	WGS	BAM^c^	R	1593.8 (580.3)	281.7 (80.7)	1875.4 (562.2)
Control-FREEC	WGS	BAM^d^	C++	1900.9 (445.4)	295.3 (42.6)	2196.2 (472.1)

^a^ The LRR and BAF data can be extracted from the final report generated by Illumina GenomeStudio.

^b^ BAM file is required by ExomeCNV for calling CNV, while VCF file is optional and used for calling CN-LOH. BAM file is processed by GATK.

^c^ BAM file is processed by a perl script distributed along with CNAnorm.

^d^ BAM file is processed by SAMtools.

^e^ Computational time is unified to single thread CPU time in minute. The list values are average time across samples with standard deviation included in parentheses.

#### WGS versus SNP array SCNA signal

**Fig 8:**
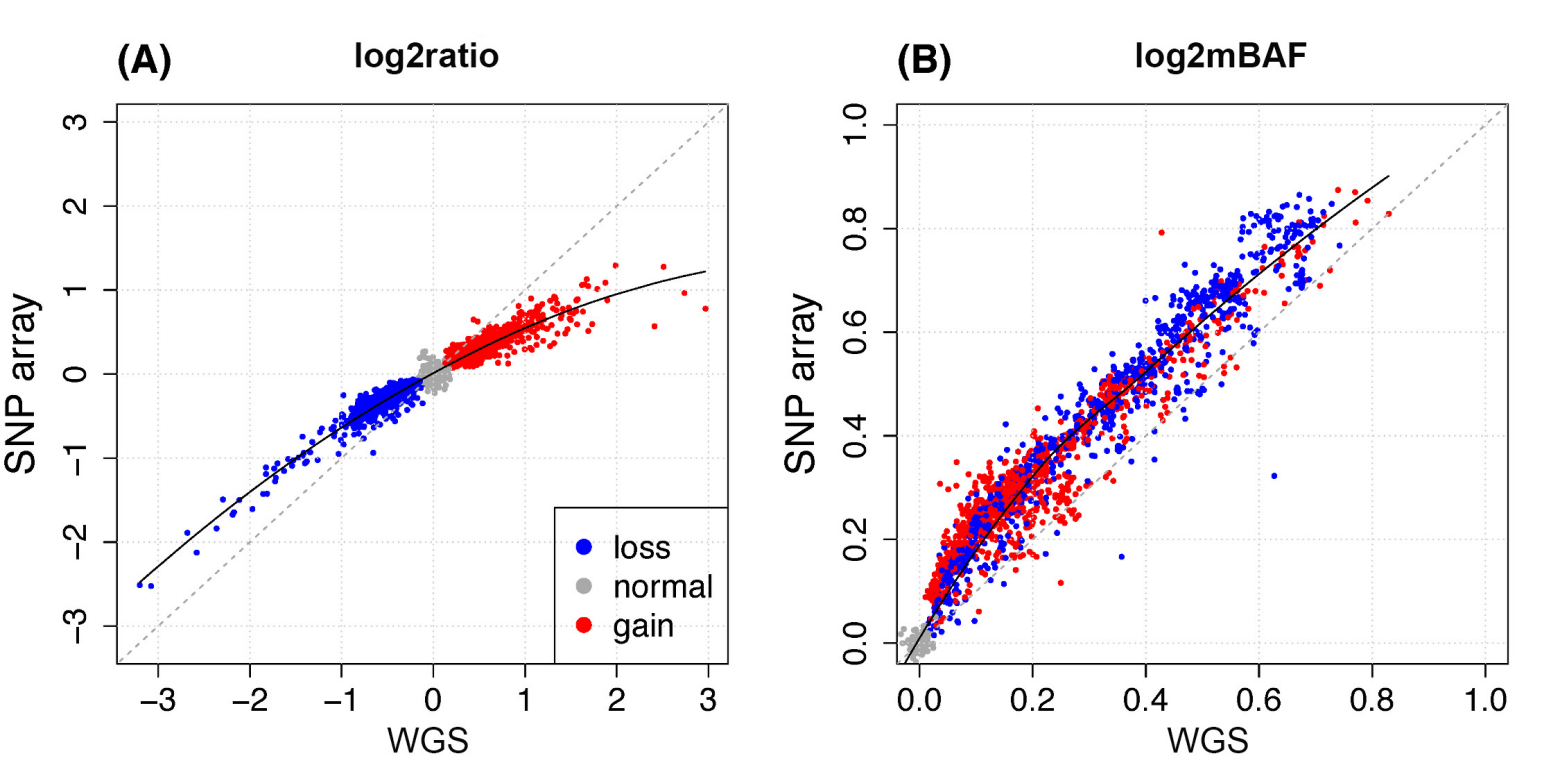
Comparison of SCNA signals between SNP array and WGS from Dataset II. (A) log2ratio; (B) log2mBAF. Each dot indicates the median signal value of a genomic segment with concordant SCNA status and >50% overlap between the results from the two platforms using SAAS-CNV. The dashed line indicates the line with slope 1. Solid line is local polynomial regression (LOESS) fit to data points.

Provided the large sample size, it is interesting to compare the quality of SCNA signals generated by WGS and SNP array using SAAS-CNV. The two dimensions (log2ratio and log2mBAF) were largely consistent between the two platforms (Fig 8). In log2ratio dimension (Fig 8A), SNP array exhibited saturation effect, where larger copy numbers were underestimated. This limitation was reported previously [8,9]. In log2mBAF dimension (Fig 8B), SNP array showed slightly larger values than WGS, probably due to the bias in intensity measurement induced from the two alleles (S10 Fig) [30].

In addition to SCNA signals, we further looked into the signal-to-noise ratio (SNR) derived from WGS vs. SNP array. The SNR is defined as the ratio of the median signal to the estimated noise level (S1A Text) for each segment. Of the samples with moderate sequencing depth (n = 86), the SNR of log2ratio is roughly comparable between the two platforms (S1A Fig), but the SNR of log2mBAF from SNP array was about twice that from WGS (S11B Fig). For the two samples of high coverage (~120x), the SNR of log2raio derived from WGS was better than SNP array, and the SNR of log2mBAF is roughly comparable (S11C and S11D Fig). In contrast, when read depth was summarized per 1kb window, the strategy CNAnorm used to pool the information from neighboring reads, WGS produced improved SNR in log2ratio dimension (S12B Fig). In summary, at moderate depth of coverage (30x~60x), which is mostly seen in contemporary cancer studies, WGS performs better in quantifying the total copy number, especially the amplification with high copy numbers, but it has large variance in measuring allelic imbalance (BAF). One of the major reasons is that, in SNP array data, BAF signal is derived from the normalization of multiple samples [7]; but it is derived from each single sample in current WGS methods.

### A case study

**Fig 9:**
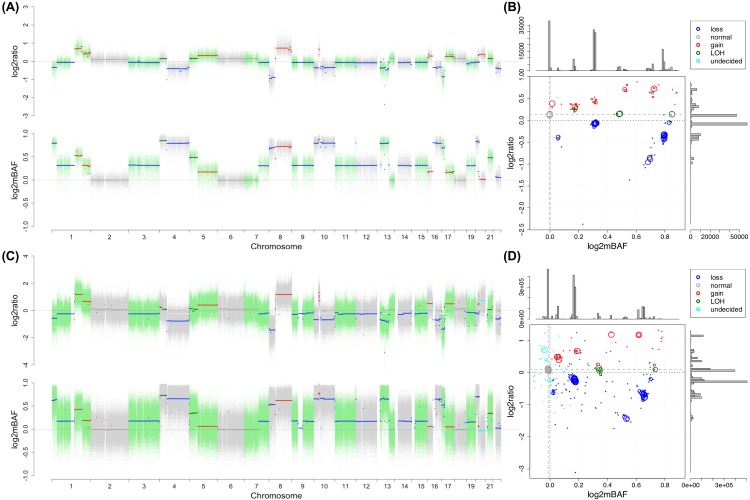
SCNA profile for the sample PT116 from Dataset II. (A) and (B) display SNP array data and (C) and (D) WGS data. In (A) and (C), on the top panel, the log2ratio signal is plotted against chromosomal position and on the bottom panel, the log2mBAF signal. The dots, each representing a locus, are colored alternately to distinguish chromosomes. The segments, each representing a DNA segment resulting from the joint segmentation, are colored based on inferred SCNA status. In (B) and (D), on the main log2mBAF-log2ratio panel, each circle corresponds to a segment in (A) and (C), with the size reflecting the length of the segment; the color code is specified in legend; dashed gray lines indicate adjusted baselines. The side panels, corresponding to log2ratio and log2mBAF dimension respectively, show the distribution of median values of each segment.

**Fig 10:**
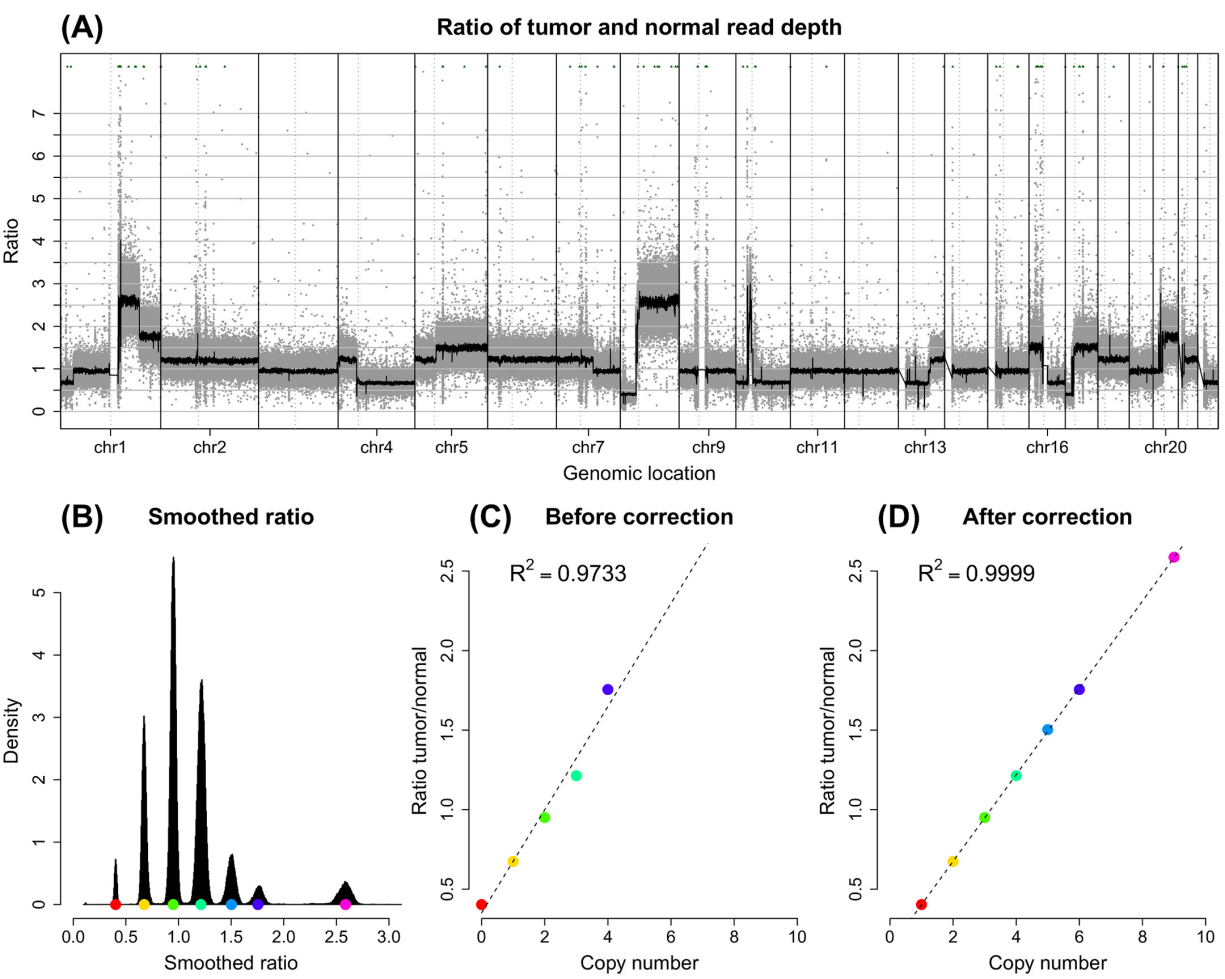
Estimation of absolute copy number and tumor purity for the sample PT116 from Dataset II. (A) The ratio of tumor versus normal read depth along the genome. The plot was generated by modifying the function plotGenome from R package CNAnorm. Grey dots represent the ratio signal calculated per 1kb window; solid thick black curve is the smoothed signal; green triangulars are points outside the graph; the vertical solid lines separate the chromosomes and the vertical dotted lines indicate the locations of centromeres. (B) Histogram of smoothed ratio signal shows seven major peaks corresponding to different copy numbers. Colored dots indicate the center of each peak. (C) Without accounting for the allelic imbalance pattern, CNAnorm only identified five out of the seven peaks and assigned them to plausible copy numbers, where the highest peak was assigned to copy number 2. (D) With the alleleic imblance pattern informed from the log2mBAF signal (Fig 9), the initial inference was manually corrected, and proper copy numbers were assigned to each of the seven peaks. Especially, the highest peak was assigned copy number 3 and the second heighest peak copy number 4. In (C) and (D), the colored dots correspond to the peak centers in (B). Dashed line is the fitted line. R-square value of fitted linear regression model was shown on the top-left corner.

We highlighted the potential usage of BAF information in improving the estimation of absolute copy number and tumor purity on the HKU HCC sample PT116 in Dataset II (Materials and Methods). This sample exhibits complicated SCNA profile (Fig 9). We applied SAAS-CNV and CNAnorm together to analyze this sample. In CNAnorm analysis, the ratio of tumor versus normal read depth was calculated per 1kb window along the genome (gray dots in Fig 10A). A smoothing approach was then employed by CNAnorm [5] to reduce the random error variability (fitted black curve in Fig 10A). The distribution of smoothed ratio signal clearly showed seven major peaks corresponding to different copy numbers (Fig 10B). CNAnorm attempted to fit a Gaussian mixture model along with Akaike’s information criterion (AIC) onto the distribution in order to identify the number of components (peaks) and their locations. However, CNAnorm was only able to identify five out of the seven peaks (Fig 10C). It then searched different configurations, where plausible copy numbers and the five identified peak centers were aligned in different ways, and chose the most likely configuration that resulted in the best correspondance. Here, the correspondance was measured by the goodness-of-fit (R^2^ value) of the linear regression model of peak centers on estimated copy numbers (Fig 10C). In this case, CNAnorm assigned the most common component (the highest peak in Fig 10A) to copy number 2 (i.e. tumor-normal ratio 1). Finally, CNAnorm estimated tumor purity (*ρ*) based on the relationship:

$$\frac{\mu_{i}}{\mu_{CN=2}}-1=(\frac{CN_{i}}{2}-1)\cdot \rho$$ (5)

where *μ*_*i*_ is the identified peak center associated with copy number *CN*_*i*_ and *μ*_*CN* = 2_ corresponds to peak center associated with copy number 2. With the estimated tumor purity, the absolute copy number of each segment was obtained (S13A Fig).

**Fig 11:**
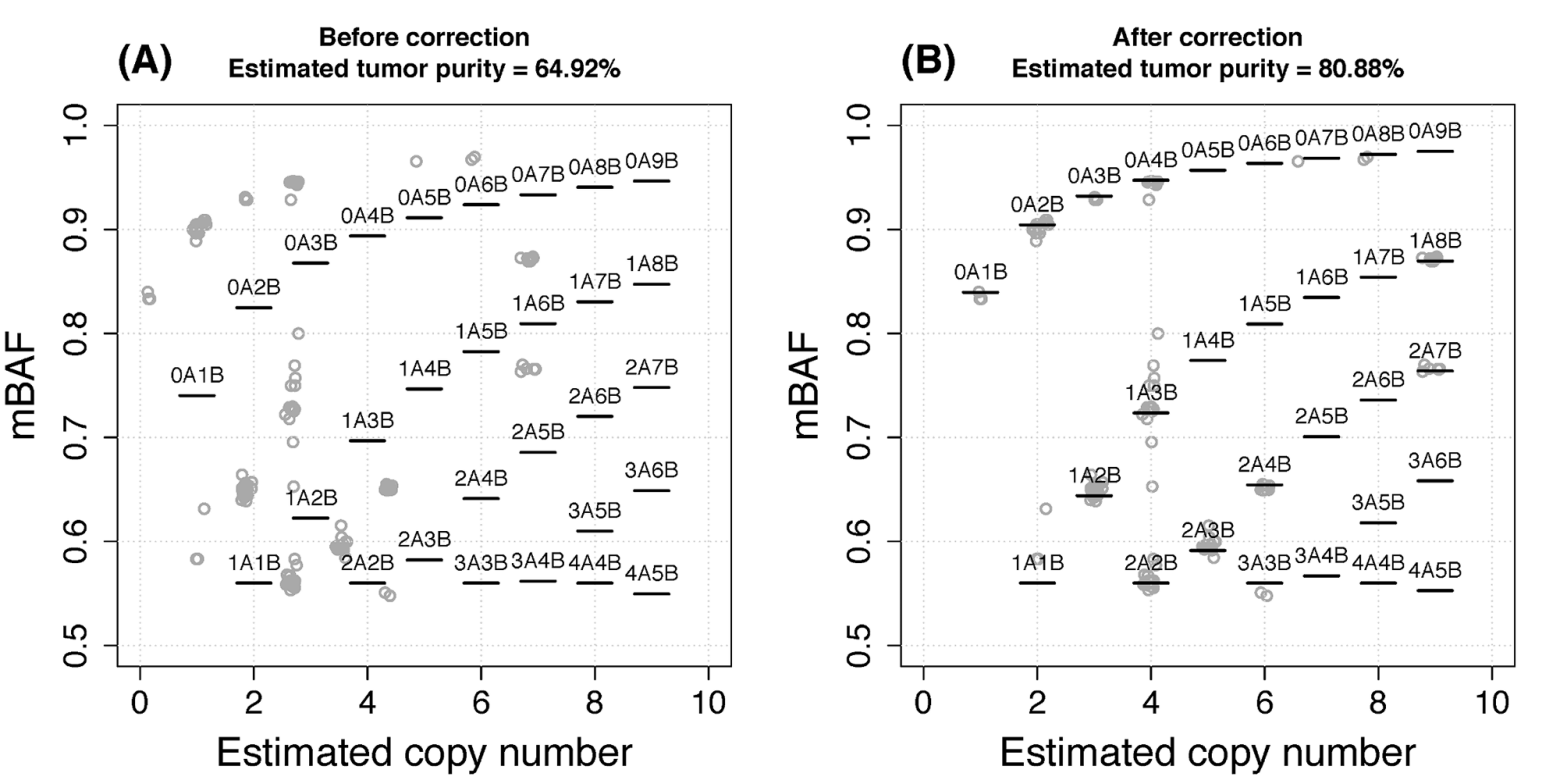
Estimated copy number and mBAF for the segments resulting from the PT116 WGS data. (A) Inference based on tumor-normal ratio signal alone. (B) Inference with BAF information incorporated for correction. In each panel, the dots represent segments with the size of at least 1Mb. The horizontal bars mark the theoretical mBAF values for different genotypes corresponding to different copy numbers (e.g., “2A3B” denotes the segment has five copies with two A allele copies and three B allele copies).

By a visual check of Fig 10B and 10C, it is obvious that CNAnorm failed to assign copy number correctly. Without accounting for the allelic imbalance pattern information (Fig 9), CNAnorm was not able to correctly infer absolute copy number in such a complicated cancer genome. For example, Chromosomes 3, 9, 11, 12, 14 and 15 were estimated to be diploid (S13A Fig), contradictory to the strong pattern of allelic imbalance manifested in log2mBAF dimension (Fig 9); Chromosomes 2, 6 and 18 were estimated to be approximately triploid, also contradictory to the log2mBAF pattern showing allelic balance (Fig 9). These observations motivated us to manually correct the inference from CNAnorm. We adjusted the correspondence between peak centers and copy numbers and as a result of the correction, the R^2^ was substantially improved (Fig 10D). The tumor purity was re-estimated with the corrected correspondence between *μ*_*i*_ and *CN*_*i*_ in Eq (5), and revised from 64.92% to 80.88%. The re-estimated copy number profile was improved in terms of better fit to integer copy numbers (horizontal black segments overlap more with the horizontal gray lines in S13B Fig). The correction also lead to strikingly better fit of the theoretical mBAF to the observed mBAF values (Fig 11). The theoretical mBAF was calculated based on the estimated copy number and tumor purity (detailed in S1K Text). Interestingly, this tumor genome likely underwent a whole-genome doubling event [31] and was estimated to be tetraploid, based on which the baseline of “normal” copy number was anchored. In summary, this case study illustrated the joint inference from BAF along with total copy number could result in more reliable estimation of tumor ploidy and purity.

## Discussion

We have developed a joint segmentation and inference approach for SCNA analysis using both total and allele-specific sequencing depth and investigated its performance with a “null” dataset and a real-world large-scale dataset. Compared with existing methods: ExomeCNV, PatternCNV, CNAnorm and Control-FREEC, our method demonstrated improved accuracy, scalability to large cancer sequencing studies, and the potential in improving the estimation of tumor ploidy and purity. Our approach exhibits flexibility and applicability in a wide range of platforms, including both deep sequencing and SNP array. These good properties can facilitate integrative cancer genomics study using multiple platforms. An R package called saasCNV, which implements our proposed appoach, is avaiable at https://zhangz05.u.hpc.mssm.edu/saasCNV/index.htm.

In contrast with germline CNV detection, characterization of SCNA in cancer genome gives rise to particular challenges, including ambiguous baseline due to aneuploidy and diluted signal pattern due to heterogeneity and normal cell contamination. For this regard, allelic specific information adds valuable input. Our method takes advantage of this information in both deep sequencing and SNP array data and achieves better capability in the correct identification of copy number baseline. We have also demonstrated that incorporating BAF could substantially improve the inference of tumor ploidy and purity. An important future work is the development of an integrated statistical model based on segment-level total sequencing depth and BAF to infer absolute copy number and tumor purity simultaneously.

SAAS-CNV features good efficiency and scalability by adopting the “top-down” strategy, which reduces the number of statistical inferences from millions to hundreds, and by taking advantage of condensed information from VCF file, which is about 1% of the size of BAM file. On Dataset II, we showed WES of moderate sequencing depth can still provide comparable specificity and sensitivity as WGS in large SCNA detection. At the same time, we acknowledge that quantifying SCNA at heterozygous loci has limitations in that not all information from mapped reads is fully utilized. While this limitation has less influence in WGS, where SNPs and indels are densely distributed on the genome, it affects the resolution in a greater degree on WES data. A possible improvement is to leverage BAMs to boost the SNR of log2ratio signal and the resolution by averaging over read depths within the windows surrounding or nearby heterozygous sites. However, it is quite time-comsumig to manipulate large BAM files. An alternative option is to utilize genomic VCF (GVCF) file, which is in the similar format as VCF, but record both variant sites and non-variant blocks. Inspired by the normalization procedure of Illumina SNP array data [7], the data processing step in our method can be further improved by incorparating multiple samples simultaneously, especially when manipulating GVCF makes it computationally feasible.

The matched tumor-normal design provides several desired features for the identification of SCNA: 1) it is biologically sensible to take matched normal genome as reference to define somatic alterations in tumor genome; 2) it is helpful to reduce the bias induced from the spatially non-uniform distribution of short reads across the genome, due to variability in GC content, exon capture efficiency, mappability of complex regions and so forth (also see S1L Text); 3) it is able to improve the normality of signals (S2 Fig), which is desirable for joint segmentation step; 4) it can help alleviate allelic signal bias commonly observed in SNP array data (S10 Fig) [30].

Lastly, our method can be used in accompany with paired-end mapping or split read methods, for example CREST [28], to refine the resolution of break points up to base pair level and verify other types of genomic rearrangements, such as intra-chromosomal and inter-chromosomal translocations, which are commonly associated with SCNAs [32].

## Supporting Information

S1 Text Supplementary methods and results. (A) Robust estimate of signal noise level. (B) Some details in analyses using SAAS-CNV. (C) Some remarks on segment merging step. (D) Some remarks on SCNA calling step. (E) ExomeCNV analysis of NA18507 WES data. (F) PatternCNV analysis of NA18507 WES data. (G) CNAnorm analysis of NA18507 WGS data. (H) Control-FREEC analysis of NA18507 WGS data. (I) Some details about the analysis of Dataset II using different methods. (J) Some comments on correlation calculation. (K) Calculation of theoretical mBAF. (L) Some remarks on GC content adjustment in data normalization.
(PDF)

S1 Fig Read depth (RD) of WGS data of 88 normal-tumor pairs in Dataset II. The x-axis is the average RD across the genome, and the y-axis is the average RD at heterozygous sites. Tumor (dark dots) and paired normal (gray dots) were sequenced at comparable depth of coverage. Gray dashed line indicates y = x. Dark dotted line is fitted on all data points. Most samples were sequenced at median of 37.6x, ranging from 24.8x to 139.4x. A large proportion of aligned reads were used for variant calling at heterozygous sites, with median proportion being 78.5% and ranging from 67.9% to 90.0%.
(PDF)

S2 Fig Normal Q-Q plots for different signals from the sample PT116 in Dataset II. (A) SNP array data. (B) WGS data. Red dots indicate heterozygous sites predicted to be involved in gain, blue dots loss, and gray dots neither gain nor loss. Dashed line connects the first and third quartiles. For SNP array data, tumor LRR is treated as log2ratio.
(PDF)

S3 Fig Size distribution and read depth (RD) of falsely detected SCNAs in NA18507 WES data. (A) The distribution of SCNA size measured as the number of loci (on log10 scale). (B) The average RD within each falsely detected SCNA region (white), spanning 10 loci, was calculated for each synthesized normal-tumor pair. As a comparison, the same number of regions (noted in the parentheses), spanning 10 loci, was randomly drawn for each pair (gray), and for each region, the average RD was also calculated. The pairs with <3 10-locus SCNAs are not included in the boxplot. The star above the short horizontal bar indicates the significance level of two-sample t-test: * p-value<0.05; ** p-value<0.01; *** p-value<0.001.
(PDF)

S4 Fig Comparison of NA18507 WES analysis results between the method using only log2ratio signal and the joint method using both log2ratio and log2mBAF signals. (A) The number of falsely called CNVs. (B) The size of falsely called CNVs. The number of false CNV calls is plotted against the correlation for each pair in the analysis with log2ratio only (C) and the joint method (D). In (A), (C) and (D), each dot represents a synthesized pair; in (A), the dashed line has the slope of 1; in (C) and (D), the dotted line indicates the fitted linear regression line.
(PDF)

S5 Fig Comparison of SCNA profiles of a sample from NA18507 WES data. (A) ExomeCNV, (B) PatternCNV and (C) SAAS-CNV. The signals for ExomeCNV and SAAS-CNV were generated by contrasting replicate 4 versus replicate 5 (S3 Table), whereas the signals for PatternCNV were generated by contrasting replicate 4 versus all the other 5 replicates. The log2ratio signal in (A), (B) and on the top panel of (C) and the log2mBAF signal on the bottom panel of (C) are plotted against chromosomal position. The dots, each representing a locus, are colored alternately to distinguish chromosomes. The dotted horizontal line is y = 0. The segments in (A) and (C) are colored based on inferred copy number status: loss–blue, normal–gray, gain–red, LOH–darkgreen, undecided–cyan. In (B), blue and red dots represent loss and gain inferred at exon level by PatternCNV. The black segments were obtained by CBS [26] for visualization purpose only.
(TIF)

S6 Fig Comparison of results from PatternCNV and SAAS-CNV on NA18507 WES data. The number of CNVs (false positives) called from PatternCNV at exon level (S1F Text) and from SAAS-CNV at segment level were compared. P-values are based on two-sample Wilcoxon signed-rank test.
(PDF)

S7 Fig Size distribution and read depth (RD) of falsely detected SCNAs in NA18507 WGS data. (A) The distribution of SCNA size measured as the number of loci (on log10 scale). (B) The average RD within each falsely detected SCNA region (white), spanning 10 loci, was calculated for each synthesized normal-tumor pair. As a comparison, the same number of regions (noted in the parentheses), spanning 10 loci, was randomly drawn for each pair (gray), and for each region, the average RD was also calculated. The star above the short horizontal bar indicates the significance level of two-sample t-test: * p-value<0.05; ** p-value<0.01; *** p-value<0.001.
(PDF)

S8 Fig Comparison of NA18507 WGS analysis results between the method using only log2ratio signal and the joint method using both log2ratio and log2mBAF signals. (A) The number of falsely called CNVs. (B) The size of falsely called CNVs. The number of false CNV calls is plotted against the correlation for each pair in the analysis with log2ratio only (C) and the joint method (D). In (A), (C) and (D), each dot represents a synthesized pair; in (A), the dashed line has the slope of 1; in (C) and (D), the dotted line indicates the fitted linear regression line.
(PDF)

S9 Fig Comparison of results from six analyses on Dataset II at different overlap rate threshold. The results from GAP analysis on SNP array data are treated as benchmark. Overlap rate threshold is (A) 10%, (B) 30%, (C) 70% and (D) 90%.
(PDF)

S10 Fig Histogram of BAF signals at heterozygous sites. Data is taken from the HKU HCC patient PT116. (A) Normal BAF. (B) Tumor BAF. (C) log2mBAF. Black solid line indicates 0.5 in (A) and (B), and 0 in (C). Red dashed line indicates median values in (A) and (B), and the baseline estimated from the analysis pipeline in (C). Gray dotted lines in (A) and (B) are used as reference to mark the relative location of each peak with respect to the black central line, about which peaks on the left and right are expected to be symmetric. Both tumor and normal BAF signals bias toward B allele.
(PDF)

S11 Fig Comparison of SCNA signal-to-noise ratio (SNR) generated from SAAS-CNV between SNP array and WGS in Dataset II. Each dot indicates a genomic segment with concordant copy number status, coded by different colors, and >50% overlap between the results from the two platforms using SAAS-CNV. SNR of log2ratio and log2mBAF is shown in samples with medium RD (n = 86) in (A) and (B), and high RD (n = 2) in (C) and (D), respectively.
(PDF)

S12 Fig Comparison of SCNA signal and signal-to-noise ratio (SNR) generated from CNAnorm between SNP array and WGS in Dataset II. Each dot indicates a genomic segment with concordant copy number status, coded by different colors, and >50% overlap from the results of the two data using CNAnorm. (A) Signal of log2ratio. (B) SNR of log2ratio.
(PDF)

S13 Fig Genome-wide copy number profile estimated from CNAnorm for PT116 in Dataset II. Also see Fig 10 in the main text. The plot was generated by modifying the function plotGenome from R package CNAnorm. (A) Results based on tumor-normal ratio signal alone before manual correction. (B) Results after correction with BAF information accounted for. In each panel, grey dots represent normalized and tumor-purity-corrected tumor-normal ratio per 1kb window; solid thick black lines are the segmented DNAcopy output; green triangulars are points outside the graph; the vertical solid lines separate the chromosomes and the vertical dotted lines indicate the locations of centromeres.
(PDF)

S1 Table Summary metrics for NA18507 WES data. (PDF)

S2 Table Summary metrics for NA18507 WGS data. (PDF)

S3 Table Correlation of read depths across loci between pairs of replicates for NA18507 WES data. (PDF)

S4 Table Correlation of read depths across loci between pairs of replicates for NA18507 WGS data. (XLSX)
