# Supplementary material for: SAAS-CNV: A Joint Segmentation Approach on Aggregated and Allele Specific Signals for the Identification of Somatic Copy Number Alterations with Next-Generation Sequencing Data
Source: PLoS Comput Biol. 2015 Nov 19;11(11):e1004618. doi: 10.1371/journal.pcbi.1004618 (PMC4652904; doi:10.1371/journal.pcbi.1004618)
Supplement: S1 Text — (A) Robust estimate of signal noise level. (B) Some details in analyses using SAAS-CNV. (C) Some remarks on segment merging step. (D) Some remarks on SCNA calling step. (E) ExomeCNV analysis of NA18507 WES data. (F) PatternCNV analysis of NA18507 WES data. (G) CNAnorm analysis of NA18507 WGS data. (H) Control-FREEC analysis of NA18507 WGS data. (I) Some details about the analysis of Dataset II using different methods. (J) Some comments on correlation calculation. (K) Calculation of theoretical mBAF. (L) Some remarks on GC content adjustment in data normalization. (PDF) [file pcbi.1004618.s001.pdf]

Supplementary Methods and Results

(A) Robust estimate of signal noise level

Assume $(x_1,\ldots,x_{2n})$ to be a series of signals ordered along chromosomal locations, following the piece-wise constant structure, i.e.,

$$x_i = \mu_k + \varepsilon_i \text{, for } i \in [i_{k-1}, i_k),\ k = 1,\ldots,K,$$

where i_k's are change points and $K << 2n$; ε_i's are independently and identically distributed with mean 0 and variance σ^2. Define $\Delta_i = x_{2i-1} - x_{2i}$ for $i = 1,\ldots,n$. Then Δ_i's are mutually independent with variance $2\sigma^2$, and mean 0, except for those sitting at change points. We can estimate the standard deviation of Δ_i by a robust estimator (e.g., median absolute deviation (MAD)), and denote it as $\hat{\sigma}_\Delta$. Then a robust estimate of the standard deviation, here referred to as signal noise level, is

$$\hat{\sigma} = \hat{\sigma}_\Delta / \sqrt{2}.$$

The method is generally valid, for in a common genome-wide CNV data the number of change points is far smaller than the number of probes.

(B) Some details in analyses using SAAS-CNV

We applied SAAS-CNV to NA18507 WES and WGS data, and Hong Kong University (HKU) hepatocellular carcinoma (HCC) WGS (Kan et al., 2013; Sung et al., 2012), synthesized WES, and SNP array data (Lamb et al., 2011). For WES/WGS data, the input VCF files were generated with an NGS data analysis pipeline implemented according to recommended GATK best practice [1]. The version of GATK [2-4] was 2.5.3, BWA [5] 0.6.1 and SAMtools [6] 0.1.19. The genotype of SNPs was called by UnifiedGenotyper. For SNP array data, the LRR and BAF could be found in the final report files of GenomeStudio (Illumina). Herein, we downloaded genotype and LRR data from GEO (GSE28127, http://www.ncbi.nlm.nih.gov/geo/) and BAF via personal communication with the authors of the paper [7].

Our analyses followed the steps described in the Methods section of the main text. In the joint segmentation step, we set the minimum segment to span at least 10 loci. In the

merging segments step and SCNA calling step, we set the significance p-value threshold as 0.05. For the analysis of HKU HCC WES data, we skipped the merging step to allow for better sensitivity.

For each analysis, the pipeline output a list of segments, each of which was called as gain, loss, CN-LOH, normal or undecided. For NA18507 WES and WGS data, the called gain, loss, and CN-LOH segments were counted towards false positives. We only considered results for autosome for easy comparison between platforms and analysis methods.

(C) Some remarks on segment merging step

In the segment-merging step, we need to ensure the size of the "null" data is large enough to produce a certain number of random samples (n=1000 in this study). In a majority of cases, the number of loci in the null data is much larger than 1000 plus the number of loci within the two neighboring segments of the longest total size, so that we can draw enough distinct random samples. If this is not the case in less common scenarios, we simply capped the number of loci within resampled segments at certain upper limit. For example, if the number of loci in the "null" data is 10000, to make sure enough random samples can be drawn, we can set the upper limit to, say, 3000, such that the total size of resampled neighboring segments is 6000. This upper limit is automatically adjusted in the implementation of the saasCNV package. This procedure is still valid even though the initially identified cluster of segments is one corresponding to SCNA rather than "normal", because our purpose is to estimate the variability in medians of neighboring segments with the same SCNA status and such segments are expected to clump together within the same cluster (see Fig. 2(B) and (D) in the main text).

(D) Some remarks on SCNA calling step

Balanced gains and losses are much less common than imbalanced gains and losses. We used GAP [8] on HKU HCC SNP array data (which is used as benchmark in method comparison; see results section in the main text) to investigate the prevalence of balanced gains and losses. Please note that double deletion (i.e., 0 copy in diploidy genome) is not

considered as balanced losses, because this alteration presents signals in log2mBAF due to larger variance of BAF in 0-copy regions than normal regions. The case of two copies with heterozygous genotype in tetraploidy samples is regarded as balanced losses. As a result, a total of 16 out of 84 samples (excluding 4 samples which have larger noise and on which GAP did not perform satisfactorily) presented balanced gains and on average, 10.7% of the autosomes were affected by balanced gains among the 16 samples. A total of 2 out of 16 tetraploidy samples (as inferred and visually checked with GAP) presented balanced losses and on average, 4.5% of the autosomes were affected by balanced losses.

(E) ExomeCNV analysis of NA18507 WES data

For each synthesized pair of replicated WES data of the HapMap sample NA18507, with one regarded as tumor and the other as normal, we performed CNV and CN-LOH analysis using `R` package `ExomeCNV` (version 1.4) [9] and following the guidance at: https://secure.genome.ucla.edu/index.php/ExomeCNV_User_Guide. We used the recommended GATK command to extract read depth information at each exon, which was taken as input in the workflow.

The CNV analysis consists of three major steps: 1) calculating log read depth ratio of tumor to normal (`calculate.logR`); 2) calling CNV for each individual exon (`classify.eCNV`); 3) combining exonic CNV into segments using Circular Binary Segmentation (CBS) (`multi.CNV.analyze`). The first step was performed as recommended. In the second step, we used the recommended parameters, except that we set read length (`read.len`) to be 100bp and the normal cell admixture (`admix`) to be 1, reflecting the fact that the "tumor" data is actually from normal cells and making the inference more conservative against false positives. In the third step, we still used the recommended parameters, except that we set read length (`read.len`) to be 100bp, the normal cell admixture (`admix`) to be 0.9 (for it would cause program running failure when setting it to be 1), and the significance level for CBS test to accept change-points (`alpha`) to be 1e-5, more conservative against false positive.

The CN-LOH analysis initiates with BAF information for all heterozygous sites in the exome, which is extracted from VCF files, and consists of two major steps: 1) calling CN-LOH at each heterozygous site (`LOH.analyze`); 2) combining CN-LOH at multiple sites into segments (`multi.LOH.analyze`). All parameters were set as recommended, except that, in the first step, we set type I error rate in the heterozygous-site-wise LOH test (`alpha`) to be 1e-4, and, in the second step, we set Type I error rate for the statistical test (`test.alpha`) to be 1e-4, for the inference to be more conservative against false positive.

We summarized results for autosome. The segments with copy number 1 or 3 (normal being 2) resulting from the CNV analysis and the segments called as CN-LOH resulting from the CN-LOH analysis were regarded as false positives.

(F) PatternCNV analysis of NA18507 WES data

To run PatternCNV [10] on WES data of the HapMap sample NA18507, one replicate was taken as tumor in turn and the rest five replicates as normal at a time. We followed the manual (http://bioinformaticstools.mayo.edu/research/patterncnv/) and used the default parameters.

Since PatternCNV calls CNVs at exon level but does not provide segment-level CNV calls – it uses circular binary segmentation (CBS) [11] (implemented by the R package "DNAcopy") merely for visualization purpose, we were only allowed to count false positives at exon level. However, it is still reasonable to compare exon-wise false CNV calls from PatternCNV with segment-wise false CNV calls from SAAS-CNV. Take the case shown in Fig. S5 for example: A total of 8949 exons out of 368146 (2.4%) was falsely called as CNV by PatternCNV (Fig. S5(B)), whereas one out of 111 (0.9%) segment was falsely called as CNV by SAAS-CNV (Fig. S5(C)). The proportions of false calls to the number of exons/segments are not strikingly different, but the difficulty in interpreting thousands of CNV calls from PatternCNV is evident. Fig. S5(B) shows that the gains and losses (red and blue dots) called by PatternCNV are distributed all over the genome. Even though being visualized via CBS (black segments), a large number of

small segments can be visually identified as false CNV calls. In practice, the number of CNV calls really matters for the interpretability and applicability of the results in downstream analysis. Therefore, such comparison in our study is meaningful.

Overall speaking, we observed more false CNV calls from PatternCNV than SAAS-CNV (Fig. S6).

Since it is difficult to interpret the exon-wise results from PatternCNV in real data analysis and the comparison of all other methods based on Dataset II is at segment level, we did not apply it to Dataset II.

(G) CNAnorm analysis of NA18507 WGS data

For each synthesized pair of replicated WGS data of the HapMap sample NA18507, with one regarded as tumor and the other as normal, we performed CNV analysis using `R` package `CNAnorm` (version 1.4.0) [12] and following the manual (version November 1, 2013).

We used the PERL script bam2windows.pl (http://bam2windows.googlecode.com/svn/trunk/bam2windows.pl) to produce the text file used as input for CNAnorm, which computed read depth for each 1kb non-overlapping window for tumor and normal, respectively, as well as the average GC-content for each window.

The workflow consists of 6 primary steps: 1) calculating the ratio of tumor to normal read depth for each window (`dataFrame2object`); 2) correcting for GC-content (`gcNorm`); 3) smoothing the ratio signal to reduce noise (`addSmooth`); 4) fitting a Gaussian mixture model to the distribution of smoothed ratio values and subsequently a linear regression model to describe the relationship between the identified modes from the mixture model and their corresponding copy number (`peakPloidy`); 5) segmentation of the ratio signal using CBS (`addDNAcopy`); 6) normalizing ratio signal (`discreteNorm`). We carried out steps 1-3 and 6 with default parameters. In step 4, we

set maximum ploidy (`ploidyToTest`) to 7 (which is the minimum possible value to avoid program running failure), in order to suppress over-fitting of mixture model for the "null" data, which is supposed to exhibit only one mode. The model fitting method (`method`) was set as "mixture", unless the program reported failure, in case of which we changed the method to "density". In step 5, we modified the original function (`addDNAcopy`) to allow more stringent criteria for CBS (`alpha=1e-5, min.width=5, undo.splits="sdundo", undo.SD=1`) to prevent DNAnorm from generating excessive false positives.

CNAnorm is able to estimate the copy number for each segment after normalization and correction for normal cell contamination. We defined segments with 0.5 copies below identified baseline as losses and 0.5 copies above baseline as gains, which were counted towards false positives. We summarized results for autosome.

(H) Control-FREEC analysis of NA18507 WGS data

For each synthesized pair of replicated WGS data of the HapMap sample NA18507, with one regarded as tumor and the other as normal, we applied Control-FREEC [13] according to the tutorial (http://bioinfo-out.curie.fr/projects/freec/tutorial.html).

We used "samtools mpileup" as instructed to produce the text file in pileup format [6] as input to Control-FREEC. We set the size of sliding window as 1kb while using the default settings as demonstrated in the example configuration file distributed along with the package. We counted those somatic CNVs and CN-LOHs called by Control-FREEC towards false positives. Results were summarized for autosome.

(I) Some details about the analysis of Dataset II using different methods

ExomeCNV. We applied the same workflow as described in Section (E) to analyze the synthesized WES data in Dataset II. We used the default parameters as demonstrated in https://secure.genome.ucla.edu/index.php/ExomeCNV_User_Guide, except that we set read length (read.len) to be 100bp and the significance level for CBS test to accept change-points (alpha) to be 1e-5, more stringent than the default one to produce a

reasonable number of segments. CN-LOH detection was not performed separately because in accuracy comparison, CN-LOH and normal status are collapsed down to non-CNV status (see methods section in the main text).

CNAnorm. We applied the same workflow as described in Section (G) to analyze Dataset II, except that in step 4, we set `ploidyToTest=12`, the default value of the package, to allow more flexibility for mixture model fitting.

Control-FREEC. We applied the same workflow as described in Section (H) to analyze Dataset II.

(J) Some comments on correlation calculation

We computed Pearson correlation coefficient of read depths in each synthesized pair of NA18507 WES/WGS data, across heterozygous sites for SAAS-CNV, across exons for ExomeCNV and across 1kb non-overlapping windows for CNAnorm and Control-FREEC. In our data analysis, the implemented GATK pipeline set a down-sampling cap of 250 on the total read depth of reference allele and alternative allele at heterozygous loci for computationally efficient variant calling, while the read depth derived from averaging over exons or 1kb windows did not undergo down-sampling with an upper bound. We observed that the correlation could be boosted by a few exons with large read depth in WES data (Fig. S14 and Table S3), and, more sharply, by a few windows with excessive read depth in WGS data (Fig. S15 and Table S4). Therefore, we excluded the sites with read depth greater than 250 from the computation of correlation, and named it as truncated correlation (see Tables S3-4). The correlation for ExomeCNV, CNAnorm and Control-FREEC refers to the truncated correlation in the main text.

(K) Calculation of theoretical mBAF

Assume that the tumor genotype involves n_A A alleles and n_B B alleles for a segment and the tumor purity is ρ. With the definition of mBAF, we only need to calculate its value for $n_A \le n_B$.

In case of allelic imbalance (i.e., $n_A < n_B$),

$$mBAF = \frac{n_B \cdot \rho + 1 \cdot (1-\rho)}{(n_A + n_B) \cdot \rho + 2 \cdot (1-\rho)}.$$

In case of allelic balance (i.e., $n_A = n_B$),

$$mBAF = \hat{\sigma} \cdot \Phi^{-1}((0.5+1)/2) + 0.5,$$

where $\hat{\sigma}$ is a robust estimate of noise level of BAF signal (see Section (A)) and $\Phi^{-1}(\cdot)$ is the inverse normal cumulative distribution function (CDF). The formula is derived from half-normal distribution.

(L) Some remarks on GC content adjustment in data normalization

We investigated on how GC content and other factors may have influence on the signal in both Dataset I (NA18507 WES and WGS) and Dataset II (HKU HCC WGS). We constructed GC content profile at the resolution of 1kb window and associated the GC content for each locus with that of the window closest to the locus. For the "null" data (NA18507 WES and WGS), we performed analysis of variance (ANOVA) for tumor read depth by taking factors of normal read depth and GC content into account. For the real data (HKU HCC WGS), we took the segment median of tumor read depth as the CNV associated read depth for the loci within corresponding segment, where the segments were produced by SAAS-CNV. Then we performed analysis of variance (ANOVA) for tumor read depth by considering factors of CNV associated read depth, normal read depth and GC content. As shown in Table S5 below, the proportion of the variance of tumor read depth explained by GC content was negligible as compared to matched normal read depth, CNV associated read depth and even random noise (residual) in all datasetsr. Therefore, in paired tumor-normal study with the matched two samples properly processed by the same procedure in parallel, the matched normal is expected to play a dominant role in normalization of tumor data as compared to GC content. Just in case, we added an option for GC content adjustment on log2ratio in an updated version of the saasCNV package.

Table S5: Summary for ANOVA of tumor read depth

Data	CNV RD (%)	Normal RD (%)	GC content (%)	Residual (%)
NA18507 WES	-	93.24 (2.39)	0.38 (0.55)	6.39 (2.00)
NA18507 WGS	-	45.28 (7.97)	0.95 (0.94)	53.77 (8.06)
HKU HCC WGS	30.65 (13.27)	30.00 (9.01)	0.59 (0.84)	38.75 (6.64)

RD, read depth. The values displayed are mean percentage across samples within each data respectively, with standard deviation included in the paired parentheses.

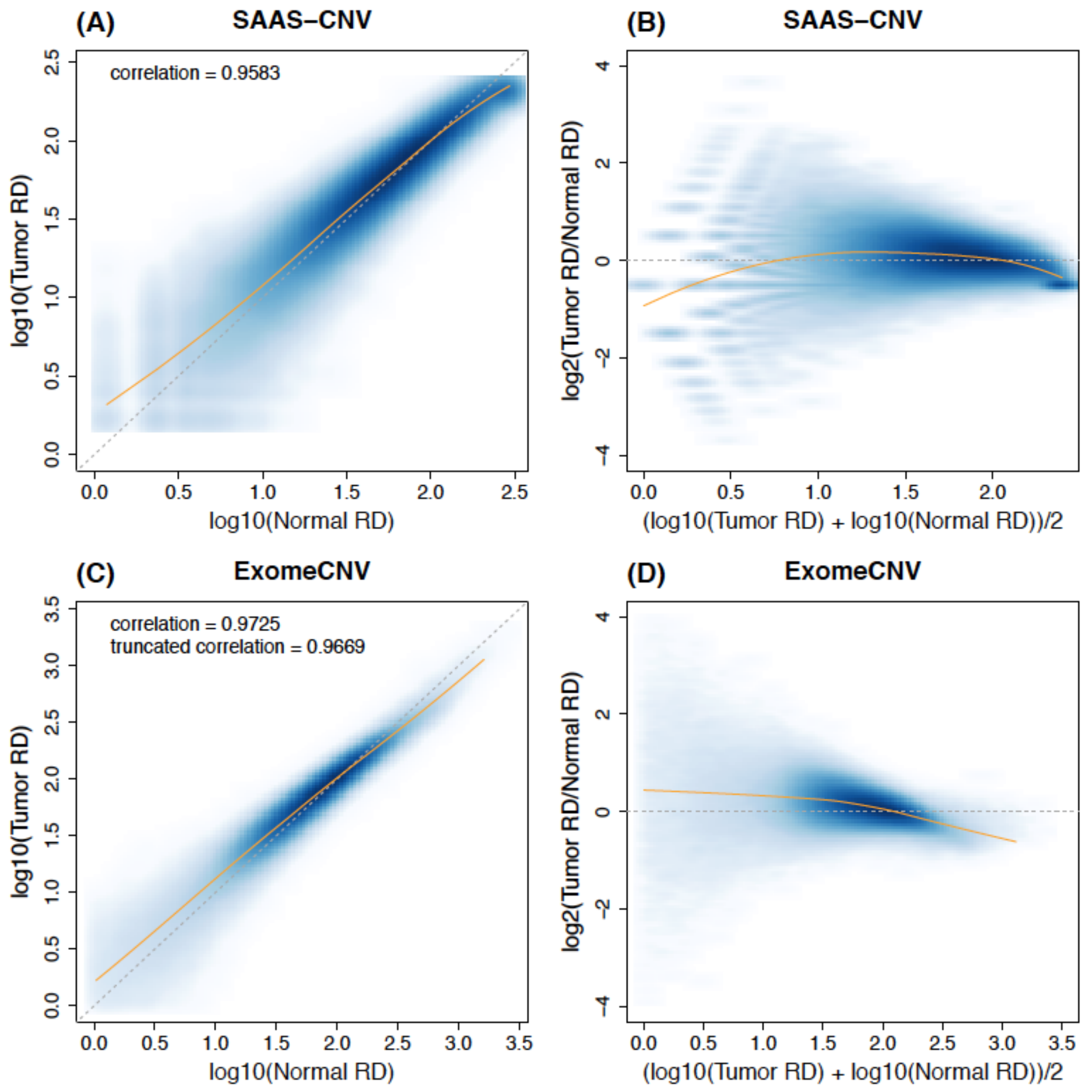

Figure S14: Read depth (RD) data of the synthesized pair Rep1_vs_Rep2 from NA18507 WES. The data points, displayed in a smoothed manner with the Bioconductor package *geneplotter*, are derived from RD information at heterozygous sites with SAAS-CNV (A-B), and at exons with ExomeCNV (C-D). RDs are normalized with the total read coverage of normal (Replicate 1) and tumor (Replicate 2) in each sample respectively. (A) and (C) are scatter plots with the x-axis being normal RD and the y-axis being tumor RD, both on log10 scale. Gray dashed line indicates y=x. (B) and (D) are mean-difference (MA) plot with the x-axis being the average RD of tumor and normal on log10 scale, and the y-axis being the log2 ratio of the RD of tumor versus normal. Gray dashed line indicates y=0. Orange line is fitted loess curve on a random sub-sample of 10,000 data points in (A)-(D). Correlations shown on top left corner of (A) and (C) are calculated on original scale (see Table S3).

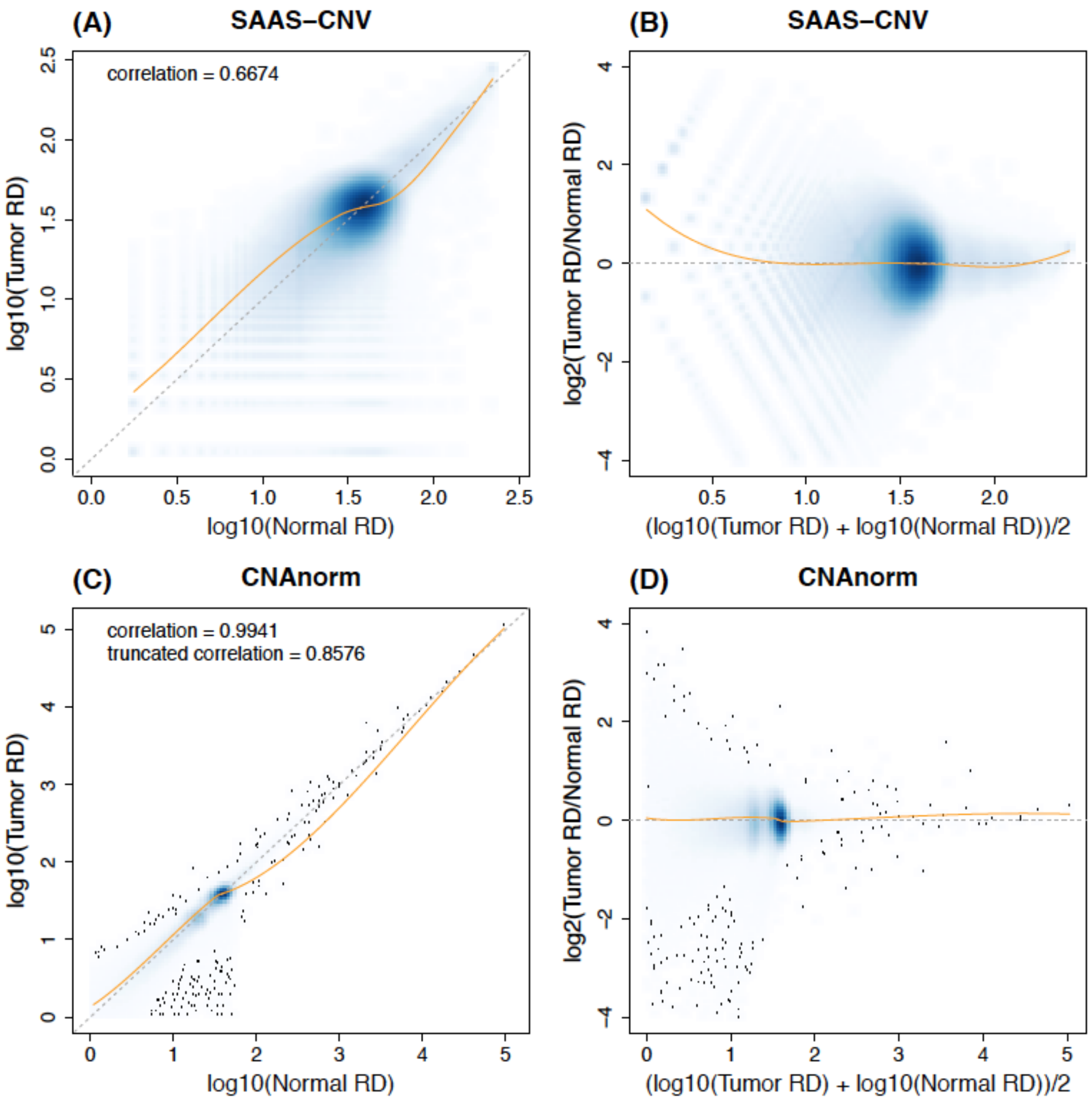

Figure S15: Read depth (RD) data of the synthesized pair Rep1_vs_Rep2 from NA18507 WGS. The data points, displayed in a smoothed manner with the Bioconductor package *geneplotter*, are derived from RD information at heterozygous sites with SAAS-CNV (A-B), and at 1kb non-overlapping windows with CNAnorm (C-D). Black dots indicate data points in local regions of low density. RDs are normalized with the total read coverage of normal (Replicate 1) and tumor (Replicate 2) in each sample respectively. (A) and (C) are scatter plots with the x-axis being normal RD and the y-axis being tumor RD, both on log10 scale. Gray dashed line indicates y=x. (B) and (D) are mean-difference (MA) plot with the x-axis being the average RD of tumor and normal on log10 scale, and the y-axis being the log2 ratio of the RD of tumor versus normal. Gray dashed line indicates y=0. Orange line is fitted loess curve on a random sub-sample of 50000 data points in (A)-(D). Correlations shown on top left corner of (A) and (C) are calculated on original scale (see Table S4).

References

1. Linderman MD, Brandt T, Edelmann L, Jabado O, Kasai Y, et al. (2014) Analytical validation of whole exome and whole genome sequencing for clinical applications. BMC Medical Genomics 7: 20.
2. DePristo MA, Banks E, Poplin R, Garimella KV, Maguire JR, et al. (2011) A framework for variation discovery and genotyping using next-generation DNA sequencing data. Nature Genetics 43: 491-498.
3. McKenna A, Hanna M, Banks E, Sivachenko A, Cibulskis K, et al. (2010) The Genome Analysis Toolkit: a MapReduce framework for analyzing next-generation DNA sequencing data. Genome Res 20: 1297-1303.
4. Van der Auwera GA, Carneiro MO, Hartl C, Poplin R, del Angel G, et al. (2011) From FastQ data to high-confidence variant calls: The Genome Analysis Toolkit best practices pipeline. Current Protocols in Bioinformatics 11: 1-33.
5. Li H, Durbin R (2009) Fast and accurate short read alignment with Burrows-Wheeler transform. Bioinformatics 25: 1754-1760.
6. Li H, Handsaker B, Wysoker A, Fennell T, Ruan J, et al. (2009) The Sequence Alignment/Map format and SAMtools. Bioinformatics 25: 2078-2079.
7. Lamb JR, Zhang C, Xie T, Wang K, Zhang B, et al. (2011) Predictive genes in adjacent normal tissue are preferentially altered by sCNV during tumorigenesis in liver cancer and may rate limiting. PLoS One 6: e20090.
8. Popova T, Manie E, Stoppa-Lyonnet D, Rigaill G, Barillot E, et al. (2009) Genome Alteration Print (GAP): a tool to visualize and mine complex cancer genomic profiles obtained by SNP arrays. Genome Biol 10: R128.
9. Sathirapongsasuti JF, Lee H, Horst BA, Brunner G, Cochran AJ, et al. (2011) Exome sequencing-based copy-number variation and loss of heterozygosity detection: ExomeCNV. Bioinformatics 27: 2648-2654.
10. Wang C, Evans JM, Bhagwate AV, Prodduturi N, Sarangi V, et al. (2014) PatternCNV: a versatile tool for detecting copy number changes from exome sequencing data. Bioinformatics 30: 2678-2680.
11. Olshen AB, Venkatraman ES, Lucito R, Wigler M (2004) Circular binary segmentation for the analysis of array-based DNA copy number data. Biostatistics 5: 557-572.
12. Gusnanto A, Wood HM, Pawitan Y, Rabbitts P, Berri S (2012) Correcting for cancer genome size and tumour cell content enables better estimation of copy number alterations from next-generation sequence data. Bioinformatics 28: 40-47.
13. Boeva V, Popova T, Bleakley K, Chiche P, Cappo J, et al. (2012) Control-FREEC: a tool for assessing copy number and allelic content using next-generation sequencing data. Bioinformatics 28: 423-425.
