# Supplementary figures and images for: SAAS-CNV: A Joint Segmentation Approach on Aggregated and Allele Specific Signals for the Identification of Somatic Copy Number Alterations with Next-Generation Sequencing Data
Source: PLoS Comput Biol. 2015 Nov 19;11(11):e1004618. doi: 10.1371/journal.pcbi.1004618 (PMC4652904; doi:10.1371/journal.pcbi.1004618)

Average RD at het. sites

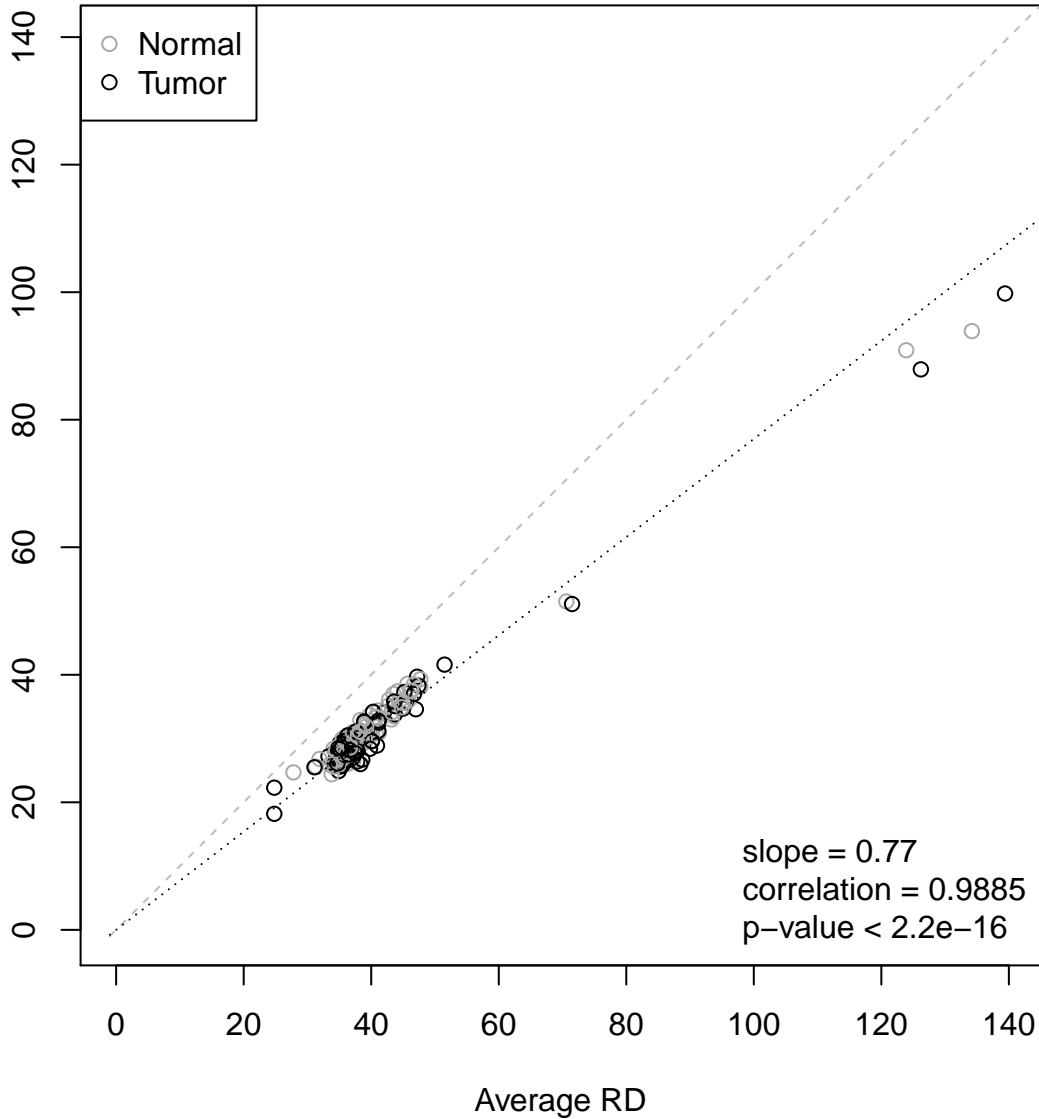

Supplement: S1 Fig — The x-axis is the average RD across the genome, and the y-axis is the average RD at heterozygous sites. Tumor (dark dots) and paired normal (gray dots) were sequenced at comparable depth of coverage. Gray dashed line indicates y = x. Dark dotted line is fitted on all data points. Most samples were sequenced at median of 37.6x, ranging from 24.8x to 139.4x. A large proportion of aligned reads were used for variant calling at heterozygous sites, with median proportion being 78.5% and ranging from 67.9% to 90.0%. (PDF) [file pcbi.1004618.s002.pdf]

(A) SNP array data

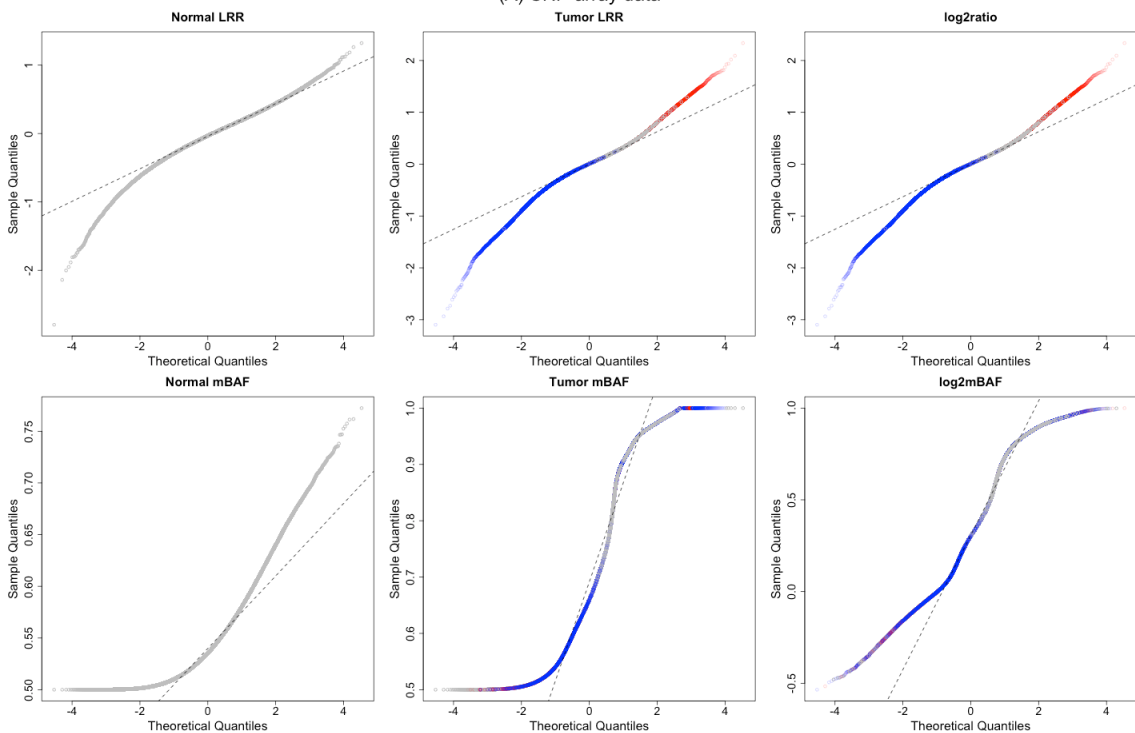

(B) WGS data

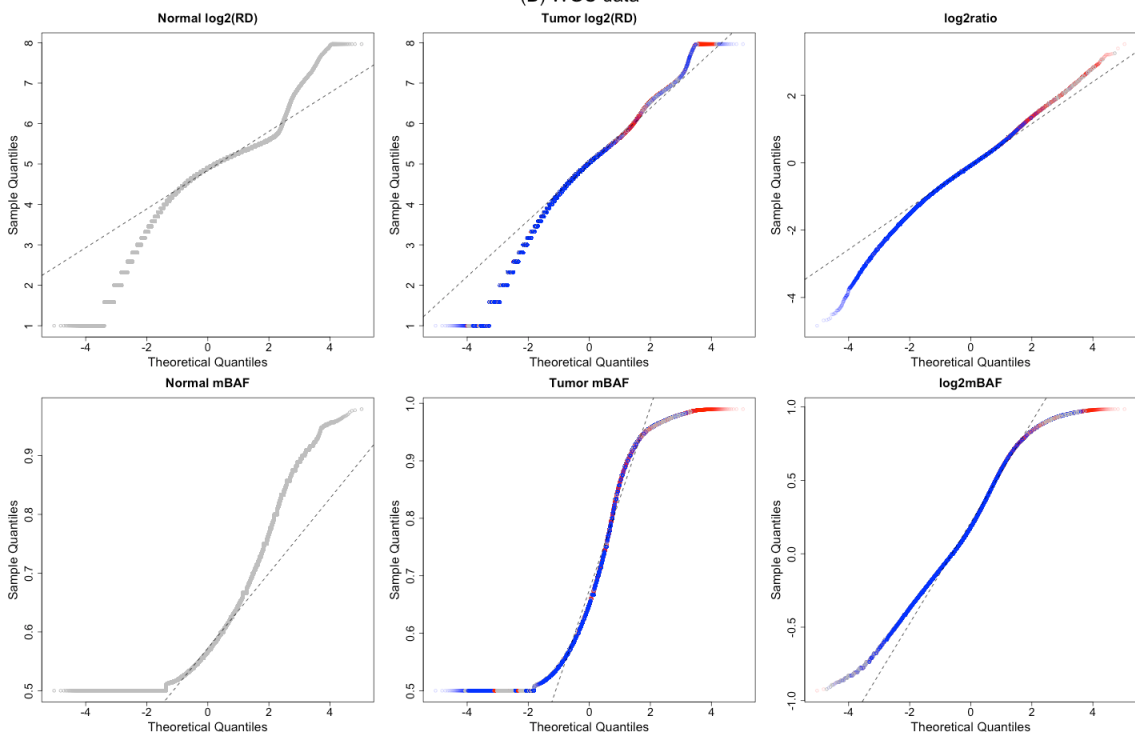

Supplement: S2 Fig — (A) SNP array data. (B) WGS data. Red dots indicate heterozygous sites predicted to be involved in gain, blue dots loss, and gray dots neither gain nor loss. Dashed line connects the first and third quartiles. For SNP array data, tumor LRR is treated as log2ratio. (PDF) [file pcbi.1004618.s003.pdf]

Frequency

(A)

## Size of SCNA

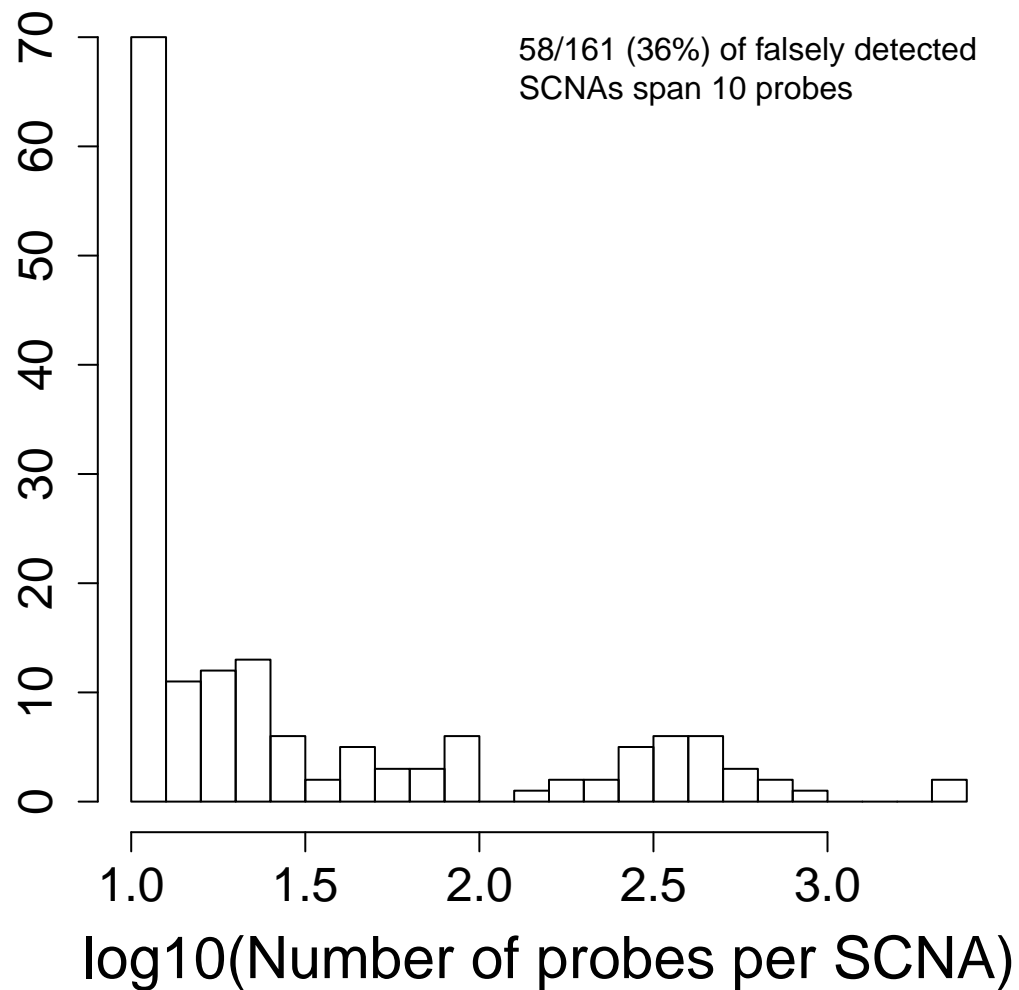

(B)

## Read depth

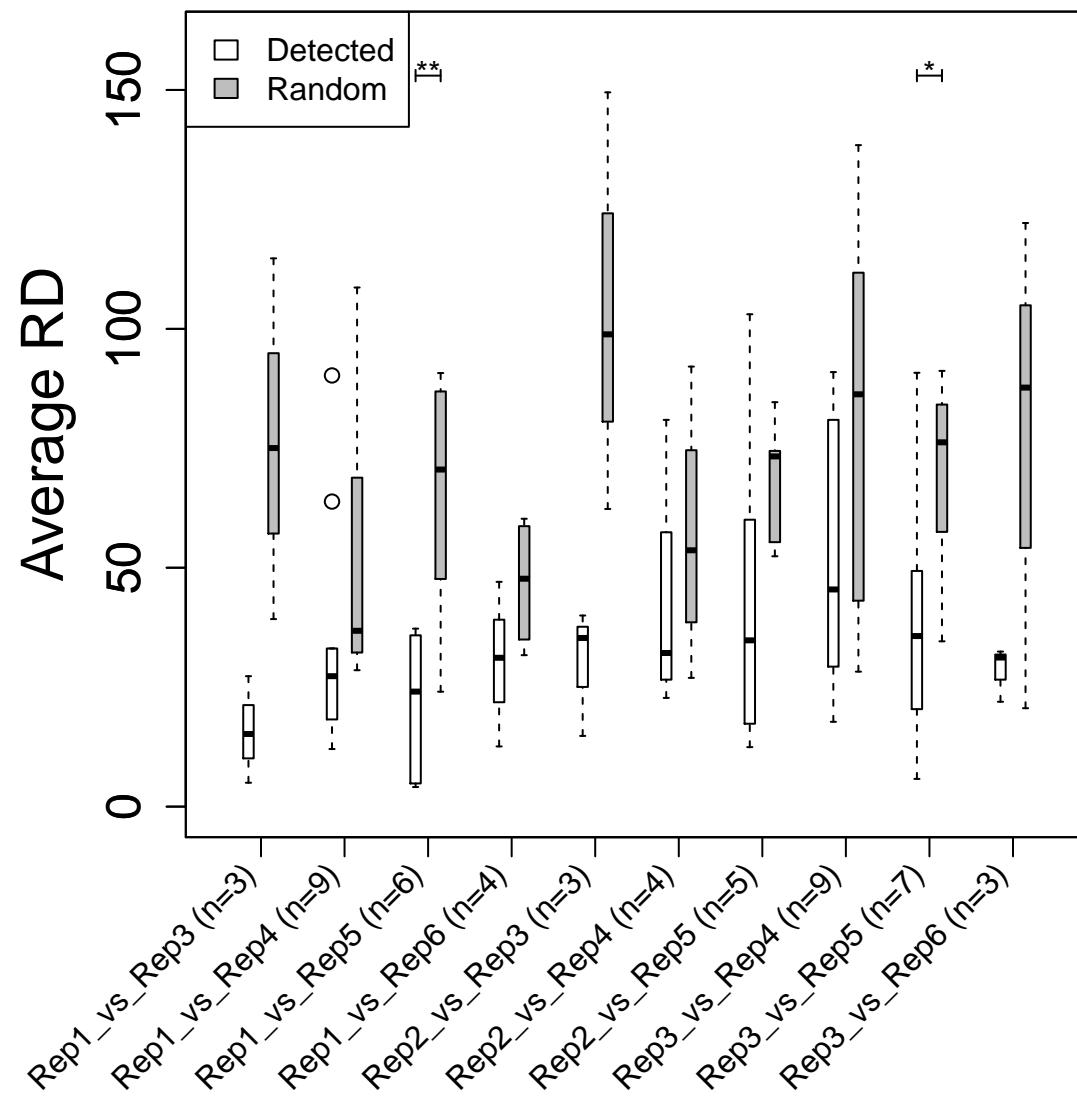

Supplement: S3 Fig — (A) The distribution of SCNA size measured as the number of loci (on log10 scale). (B) The average RD within each falsely detected SCNA region (white), spanning 10 loci, was calculated for each synthesized normal-tumor pair. As a comparison, the same number of regions (noted in the parentheses), spanning 10 loci, was randomly drawn for each pair (gray), and for each region, the average RD was also calculated. The pairs with <3 10-locus SCNAs are not included in the boxplot. The star above the short horizontal bar indicates the significance level of two-sample t-test: * p-value<0.05; ** p-value<0.01; *** p-value<0.001. (PDF) [file pcbi.1004618.s004.pdf]

**(A) False positive: CNV**

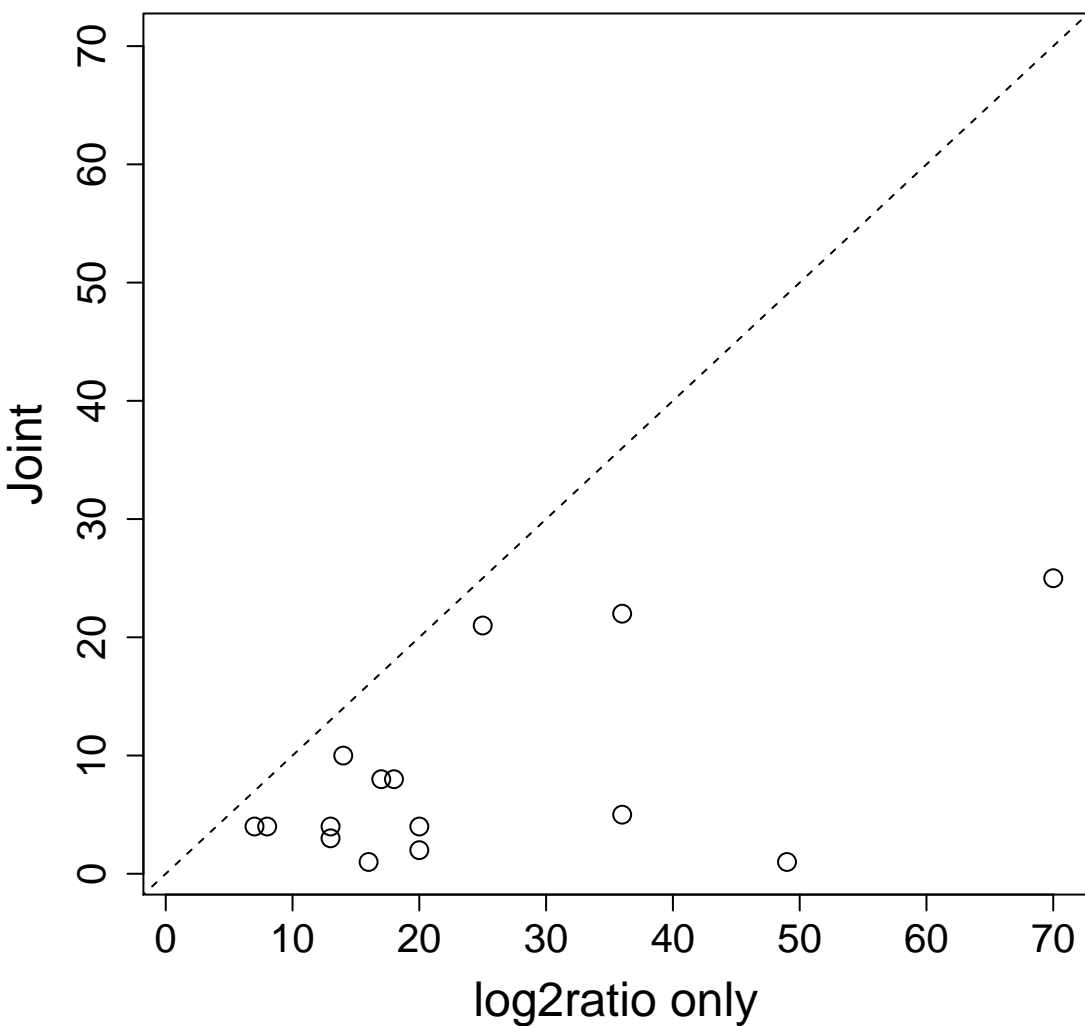

**(B) Size of false CNVs**

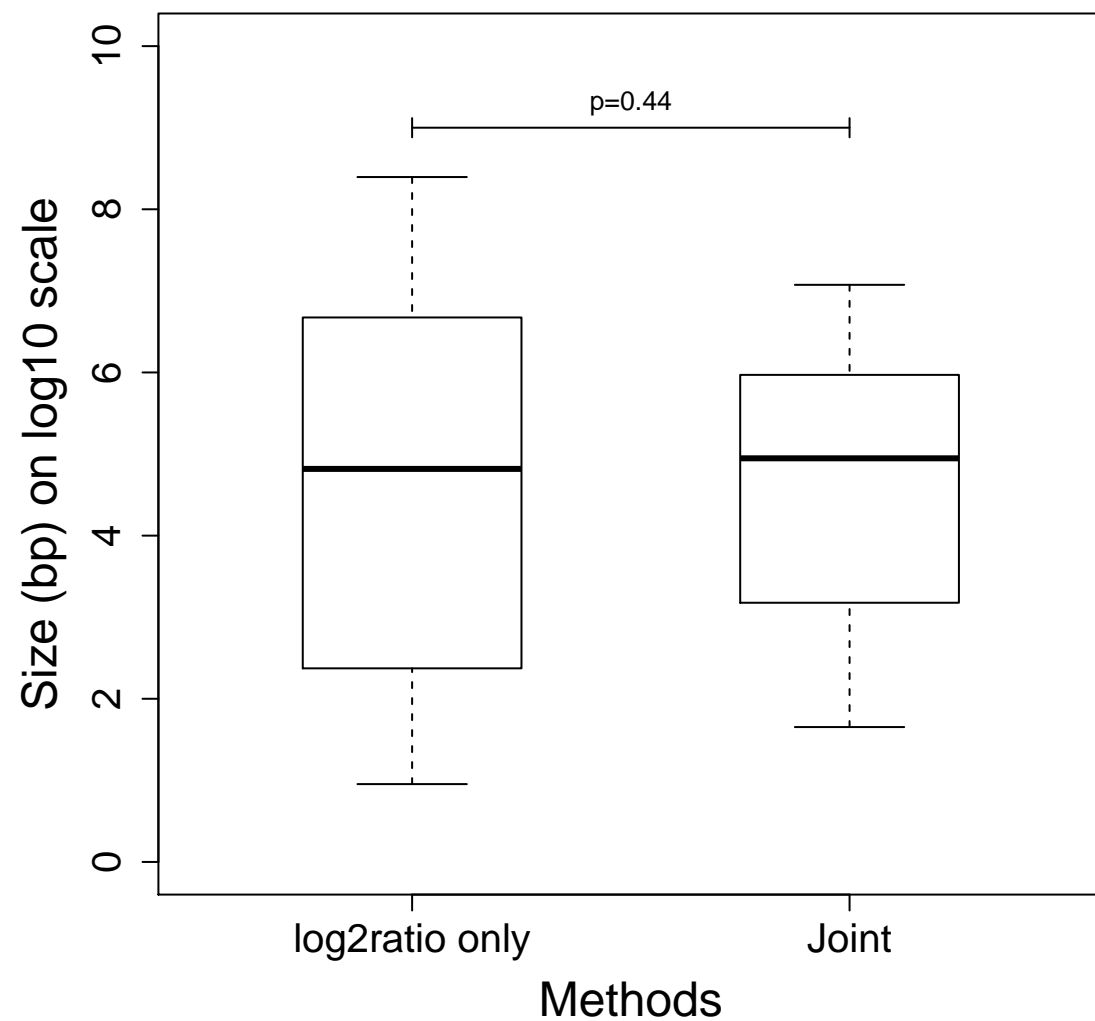

**(C) Robustness: log2ratio only**

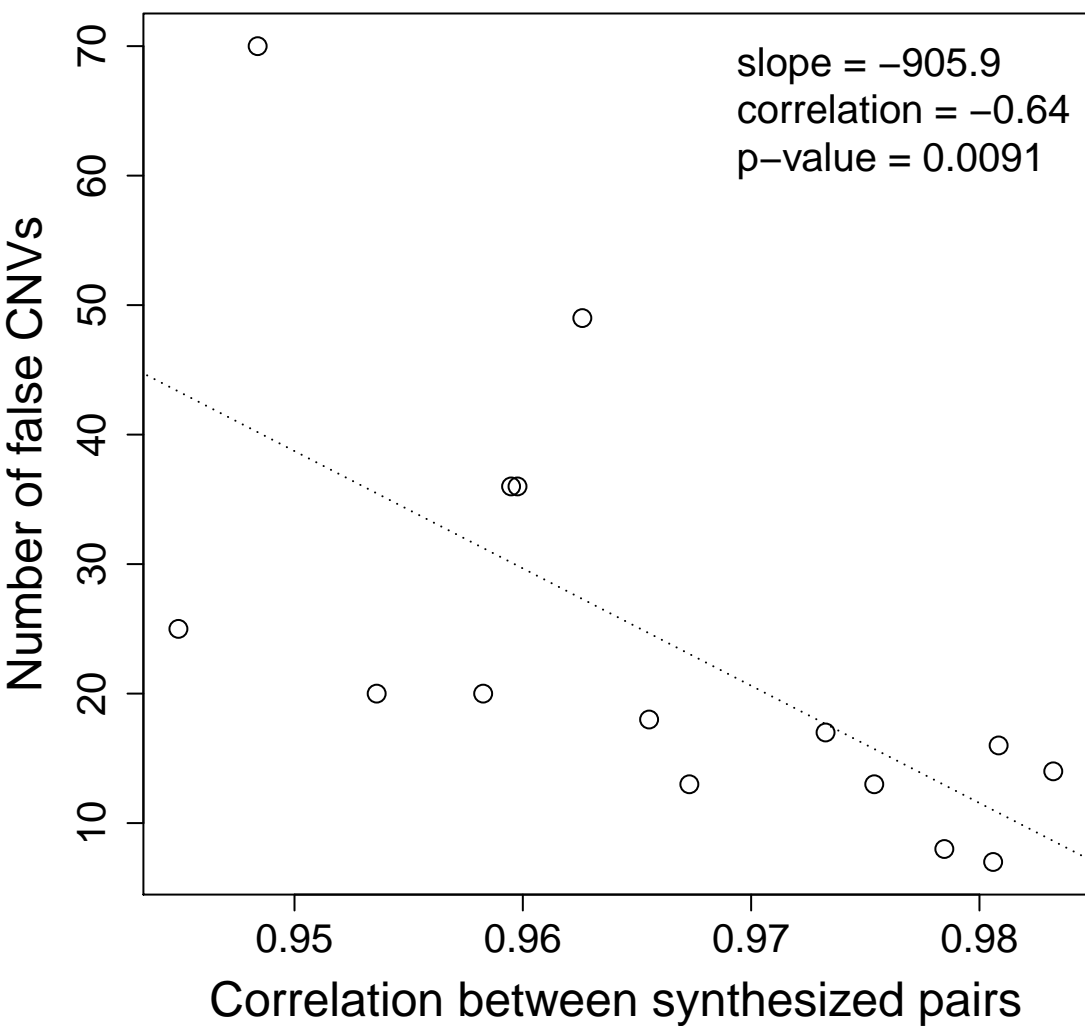

**(D) Robustness: Joint**

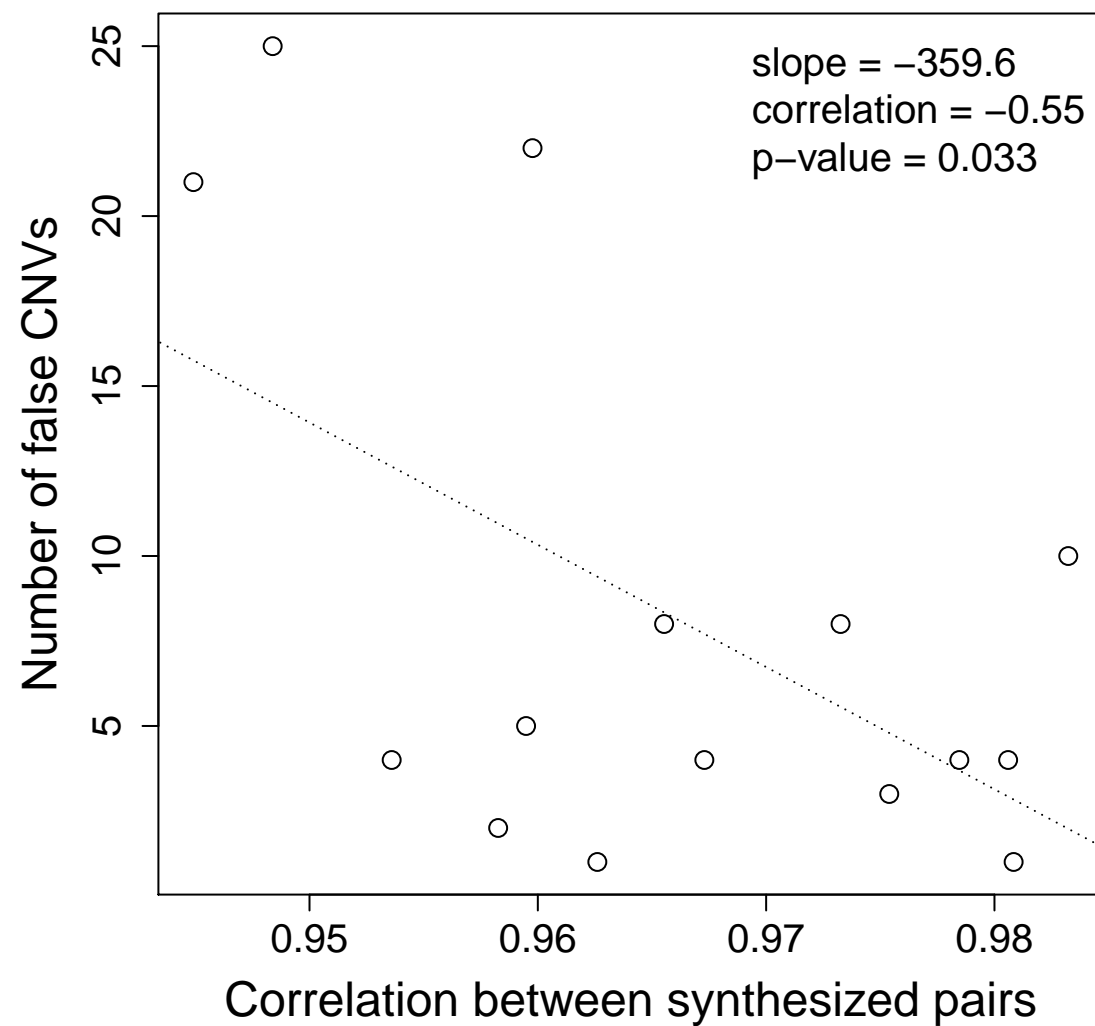

Supplement: S4 Fig — (A) The number of falsely called CNVs. (B) The size of falsely called CNVs. The number of false CNV calls is plotted against the correlation for each pair in the analysis with log2ratio only (C) and the joint method (D). In (A), (C) and (D), each dot represents a synthesized pair; in (A), the dashed line has the slope of 1; in (C) and (D), the dotted line indicates the fitted linear regression line. (PDF) [file pcbi.1004618.s005.pdf]

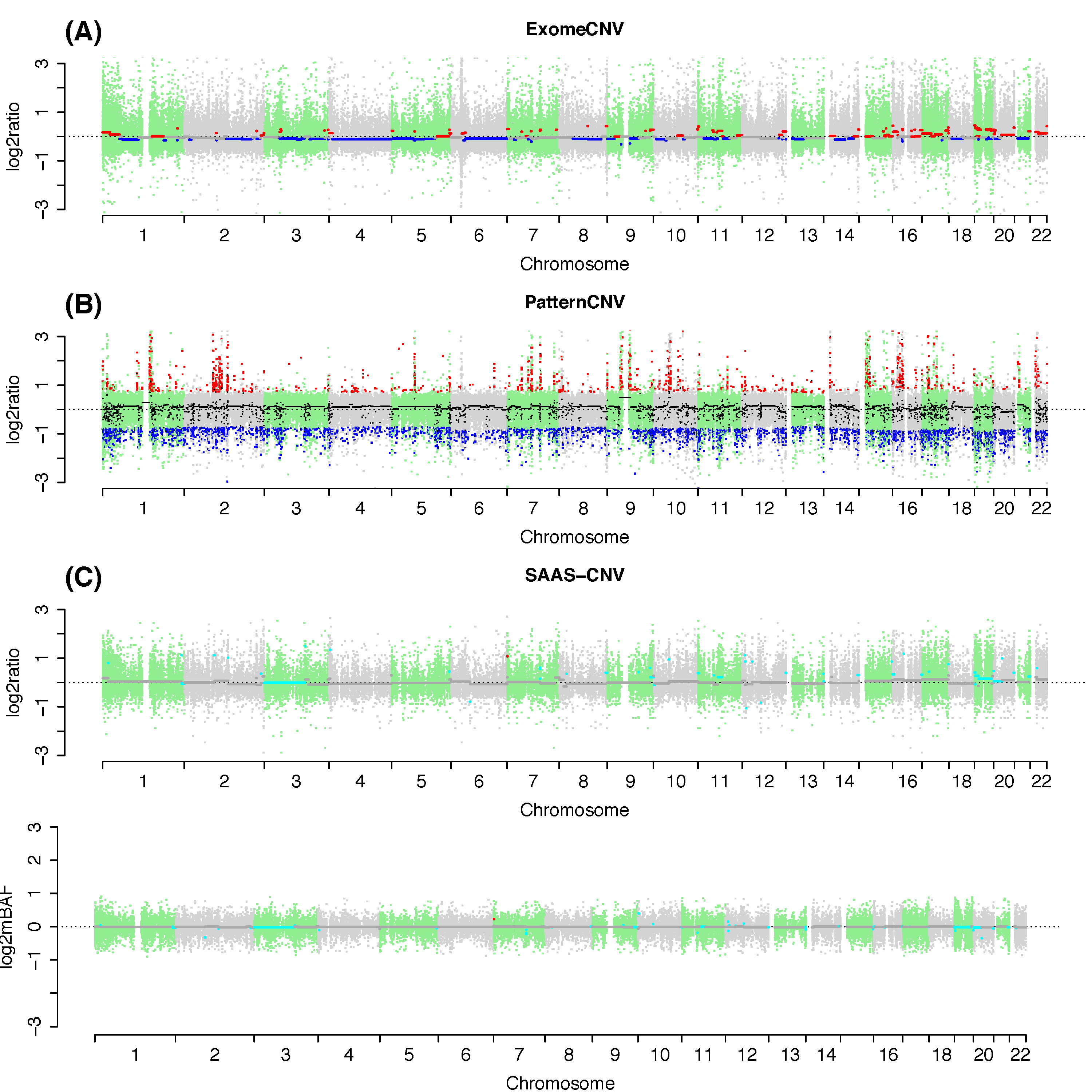

Supplement: S5 Fig — (A) ExomeCNV, (B) PatternCNV and (C) SAAS-CNV. The signals for ExomeCNV and SAAS-CNV were generated by contrasting replicate 4 versus replicate 5 (S3 Table), whereas the signals for PatternCNV were generated by contrasting replicate 4 versus all the other 5 replicates. The log2ratio signal in (A), (B) and on the top panel of (C) and the log2mBAF signal on the bottom panel of (C) are plotted against chromosomal position. The dots, each representing a locus, are colored alternately to distinguish chromosomes. The dotted horizontal line is y = 0. The segments in (A) and (C) are colored based on inferred copy number status: loss–blue, normal–gray, gain–red, LOH–darkgreen, undecided–cyan. In (B), blue and red dots represent loss and gain inferred at exon level by PatternCNV. The black segments were obtained by CBS [26] for visualization purpose only. (TIF) [file pcbi.1004618.s006.tif]

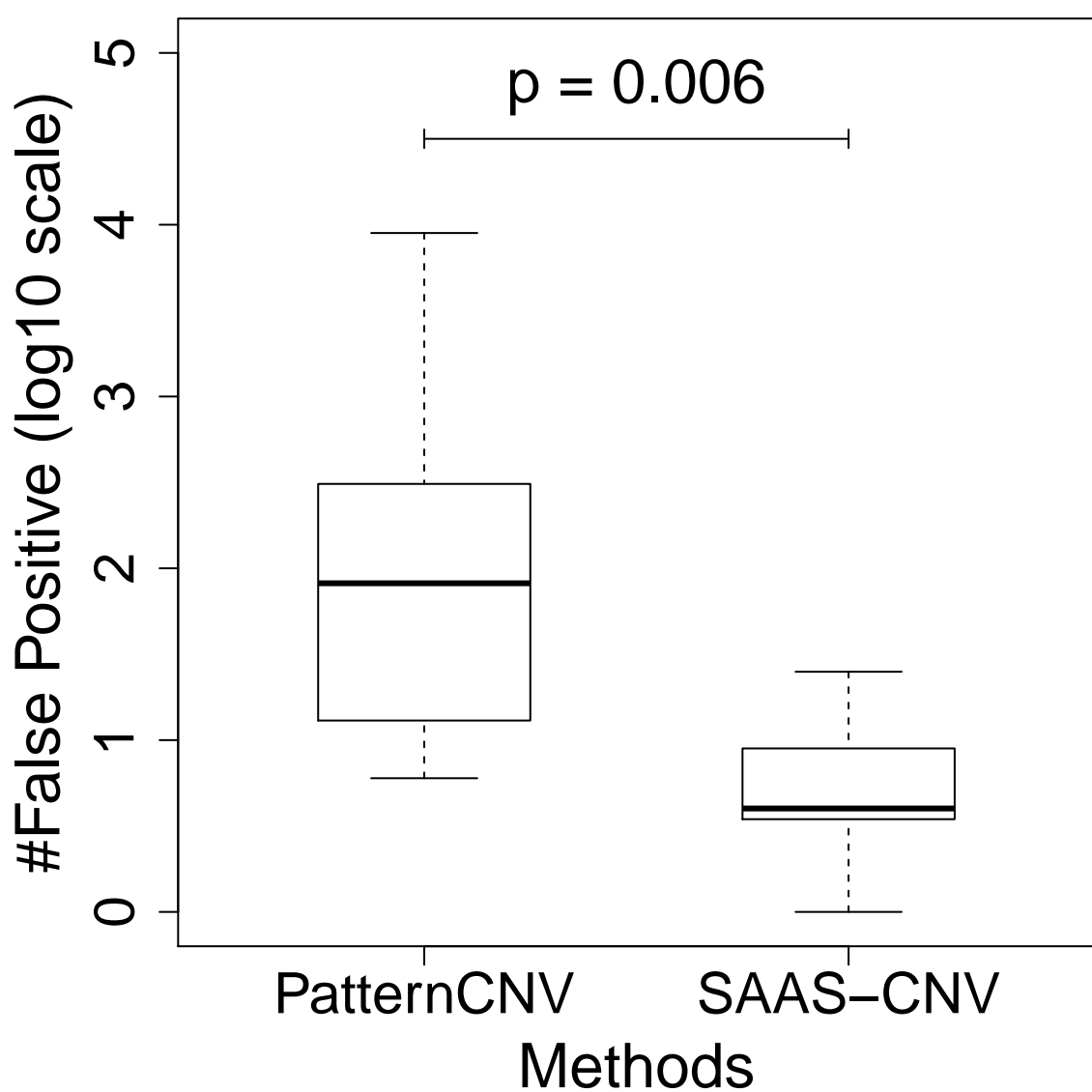

Supplement: S6 Fig — The number of CNVs (false positives) called from PatternCNV at exon level (S1F Text) and from SAAS-CNV at segment level were compared. P-values are based on two-sample Wilcoxon signed-rank test. (PDF) [file pcbi.1004618.s007.pdf]

Frequency

(A)

## Size of SCNA

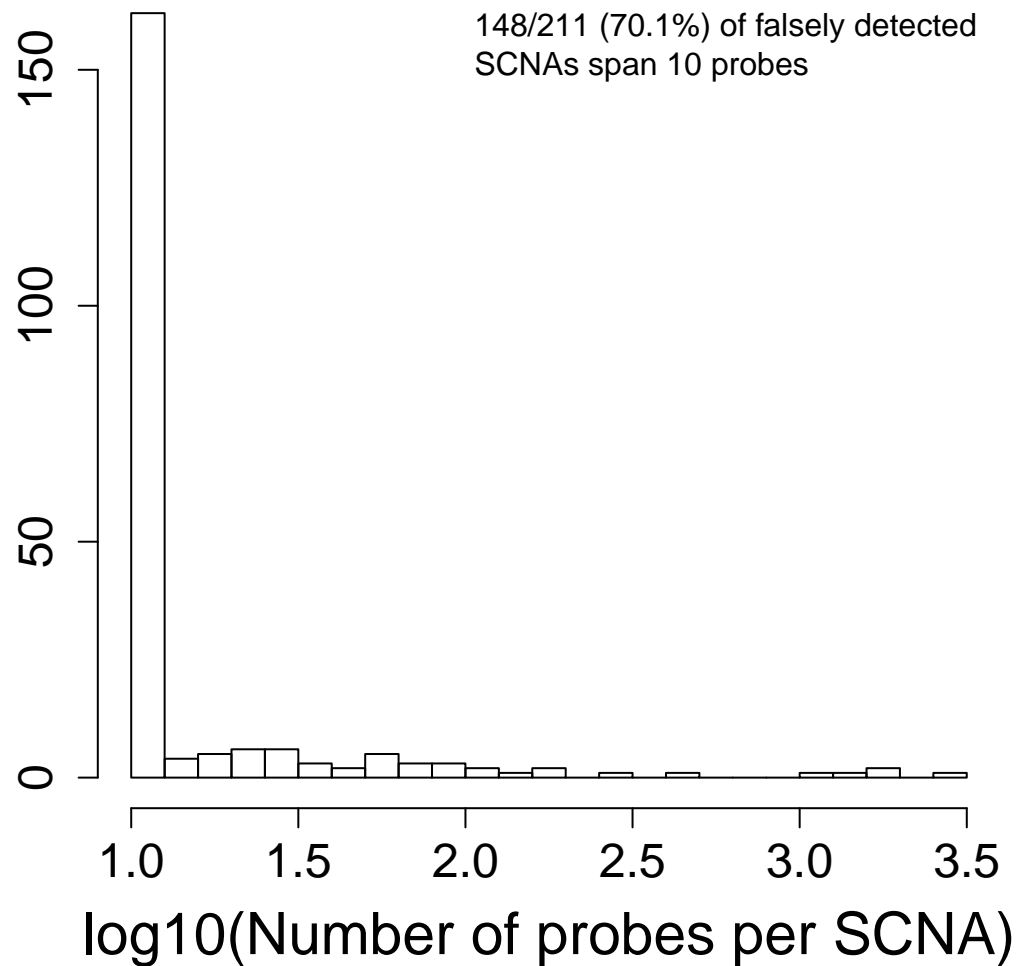

(B)

## Read depth

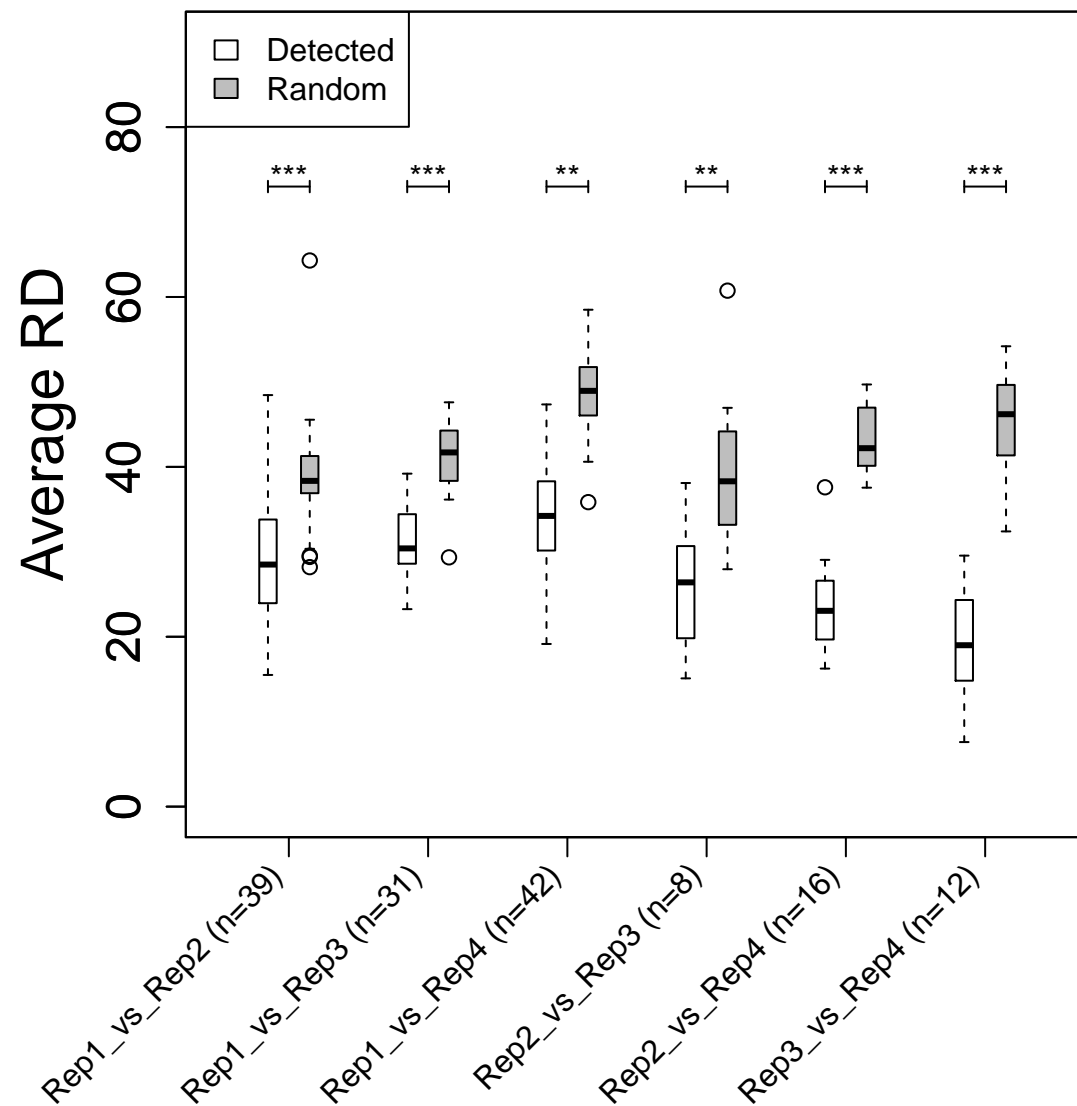

Supplement: S7 Fig — (A) The distribution of SCNA size measured as the number of loci (on log10 scale). (B) The average RD within each falsely detected SCNA region (white), spanning 10 loci, was calculated for each synthesized normal-tumor pair. As a comparison, the same number of regions (noted in the parentheses), spanning 10 loci, was randomly drawn for each pair (gray), and for each region, the average RD was also calculated. The star above the short horizontal bar indicates the significance level of two-sample t-test: * p-value<0.05; ** p-value<0.01; *** p-value<0.001. (PDF) [file pcbi.1004618.s008.pdf]

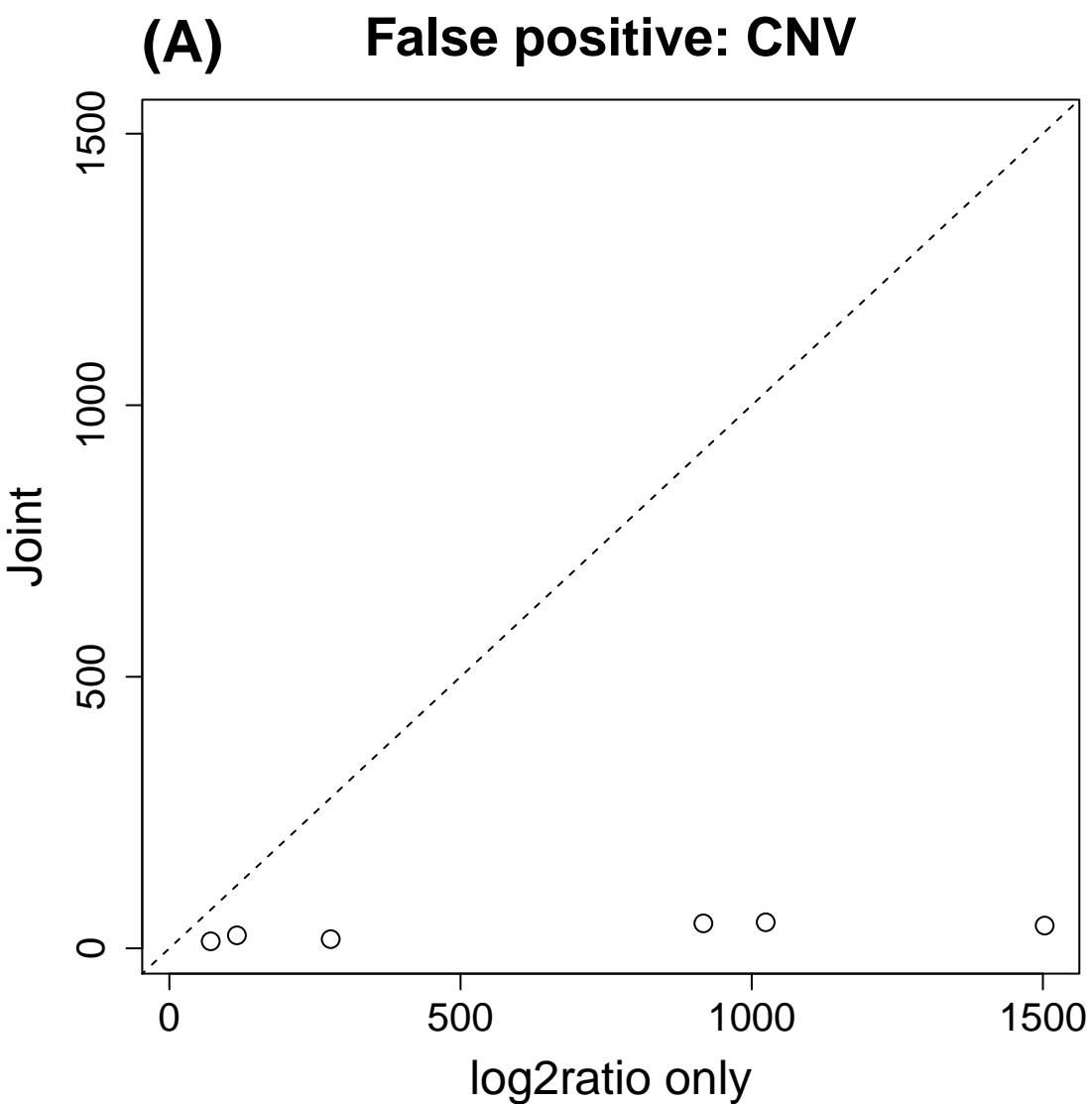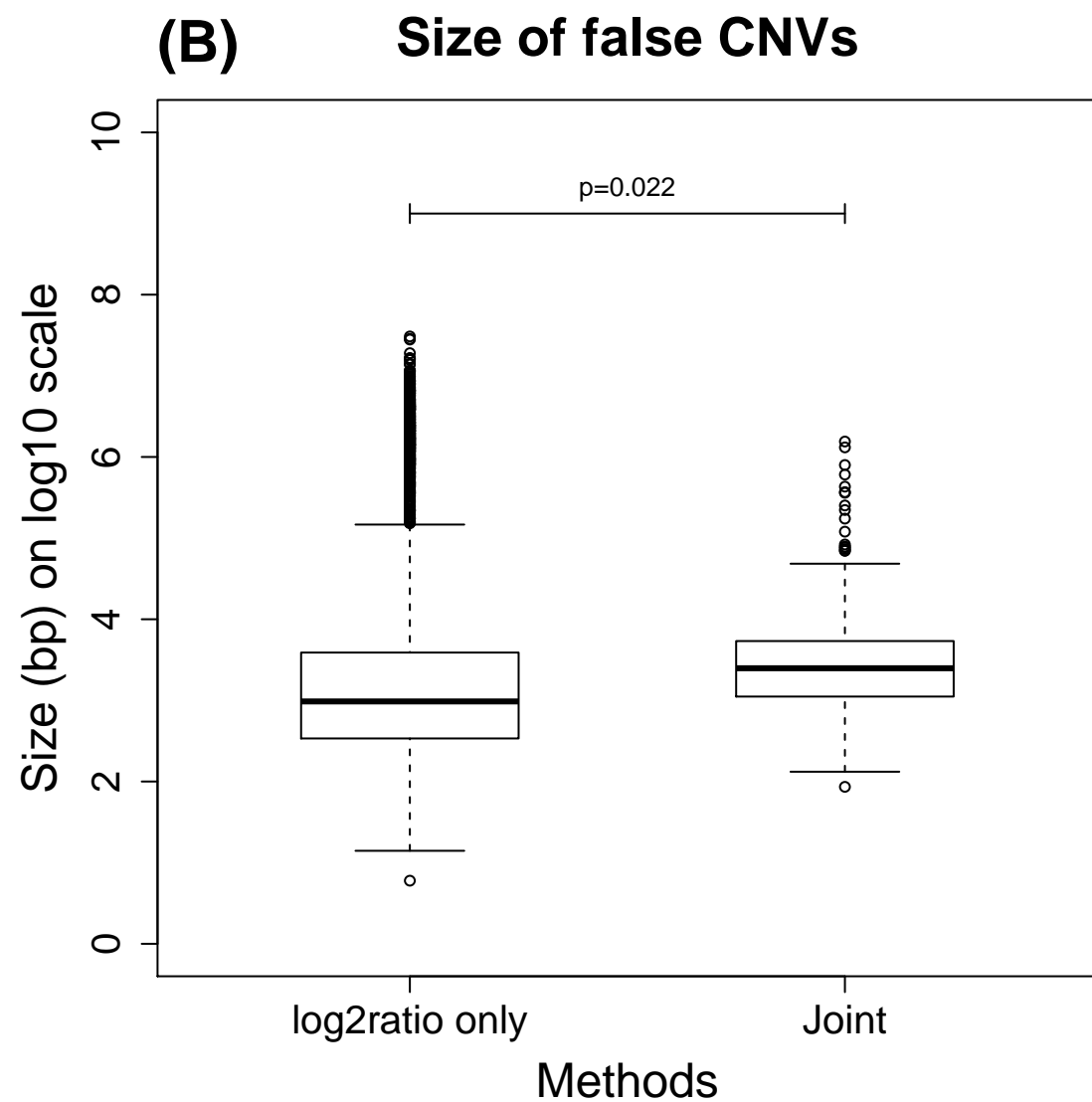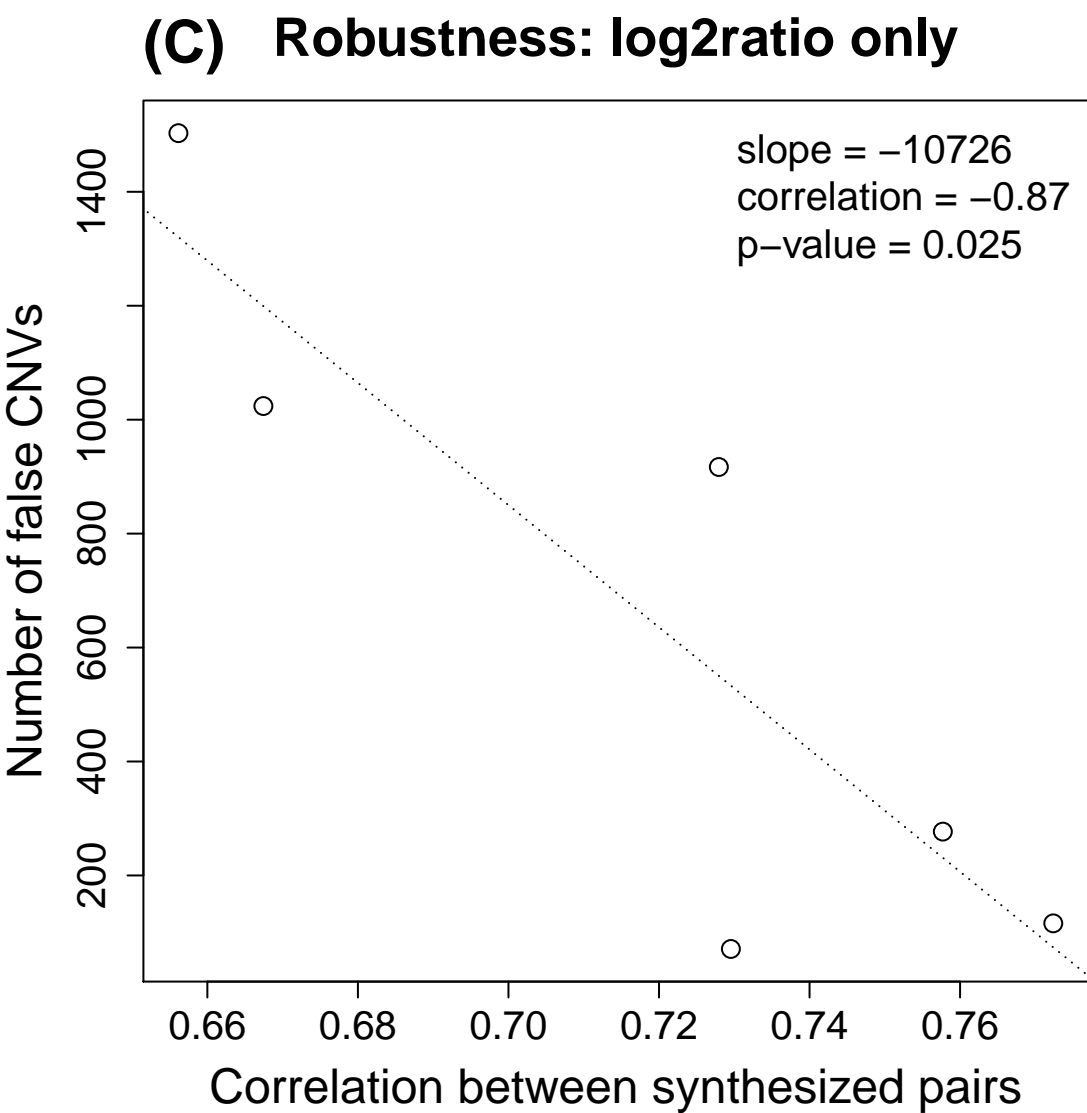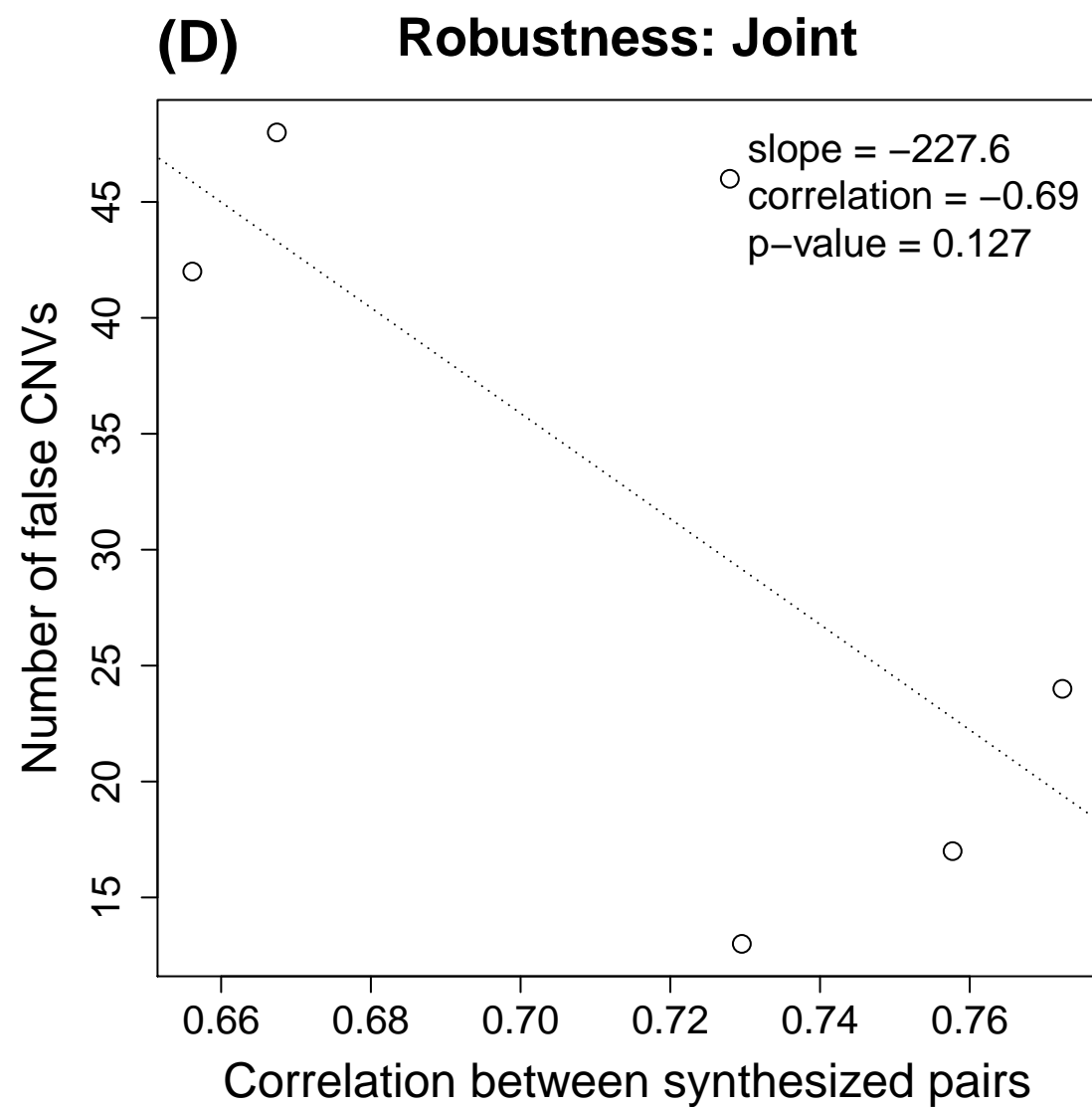

Supplement: S8 Fig — (A) The number of falsely called CNVs. (B) The size of falsely called CNVs. The number of false CNV calls is plotted against the correlation for each pair in the analysis with log2ratio only (C) and the joint method (D). In (A), (C) and (D), each dot represents a synthesized pair; in (A), the dashed line has the slope of 1; in (C) and (D), the dotted line indicates the fitted linear regression line. (PDF) [file pcbi.1004618.s009.pdf]

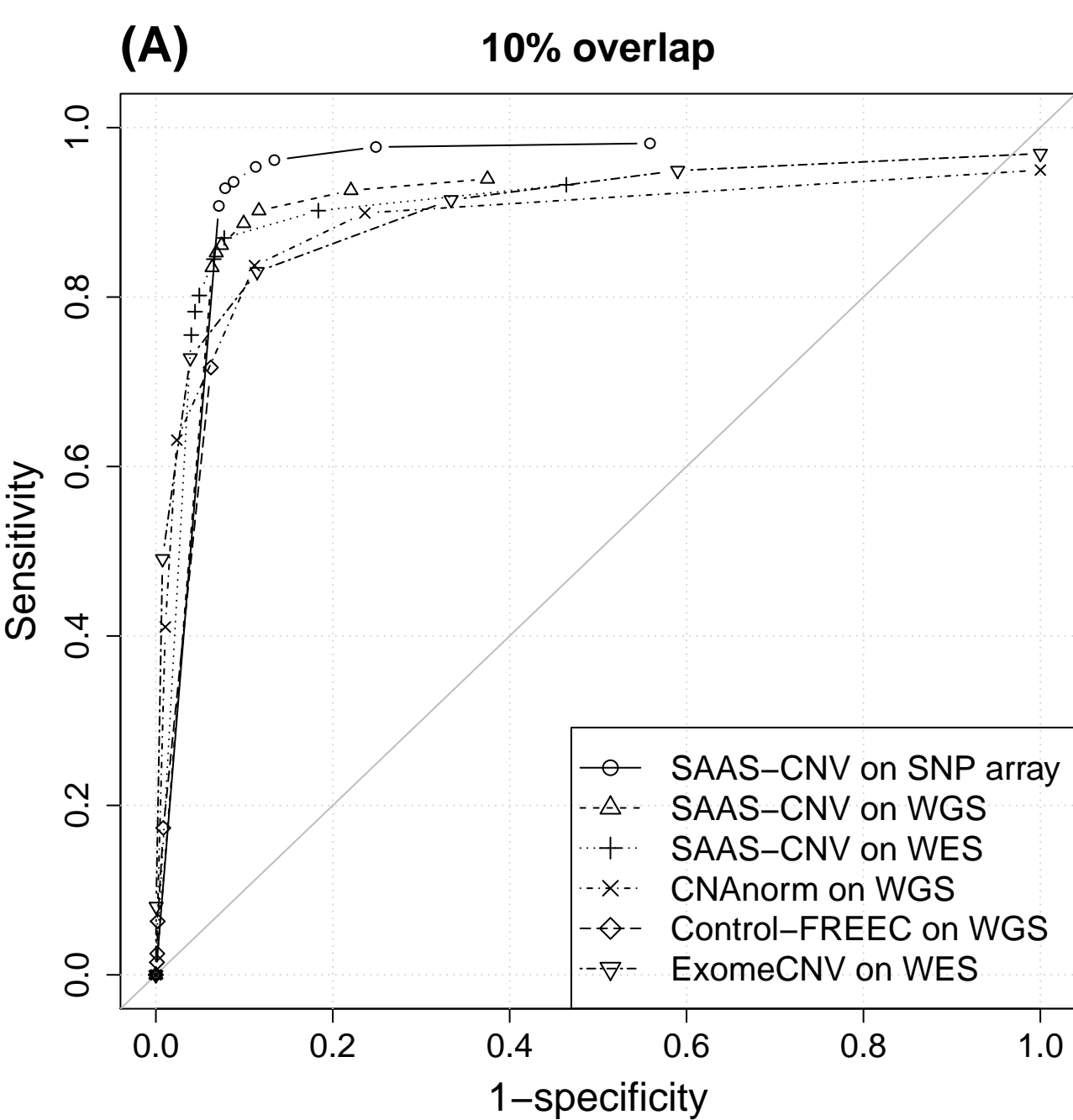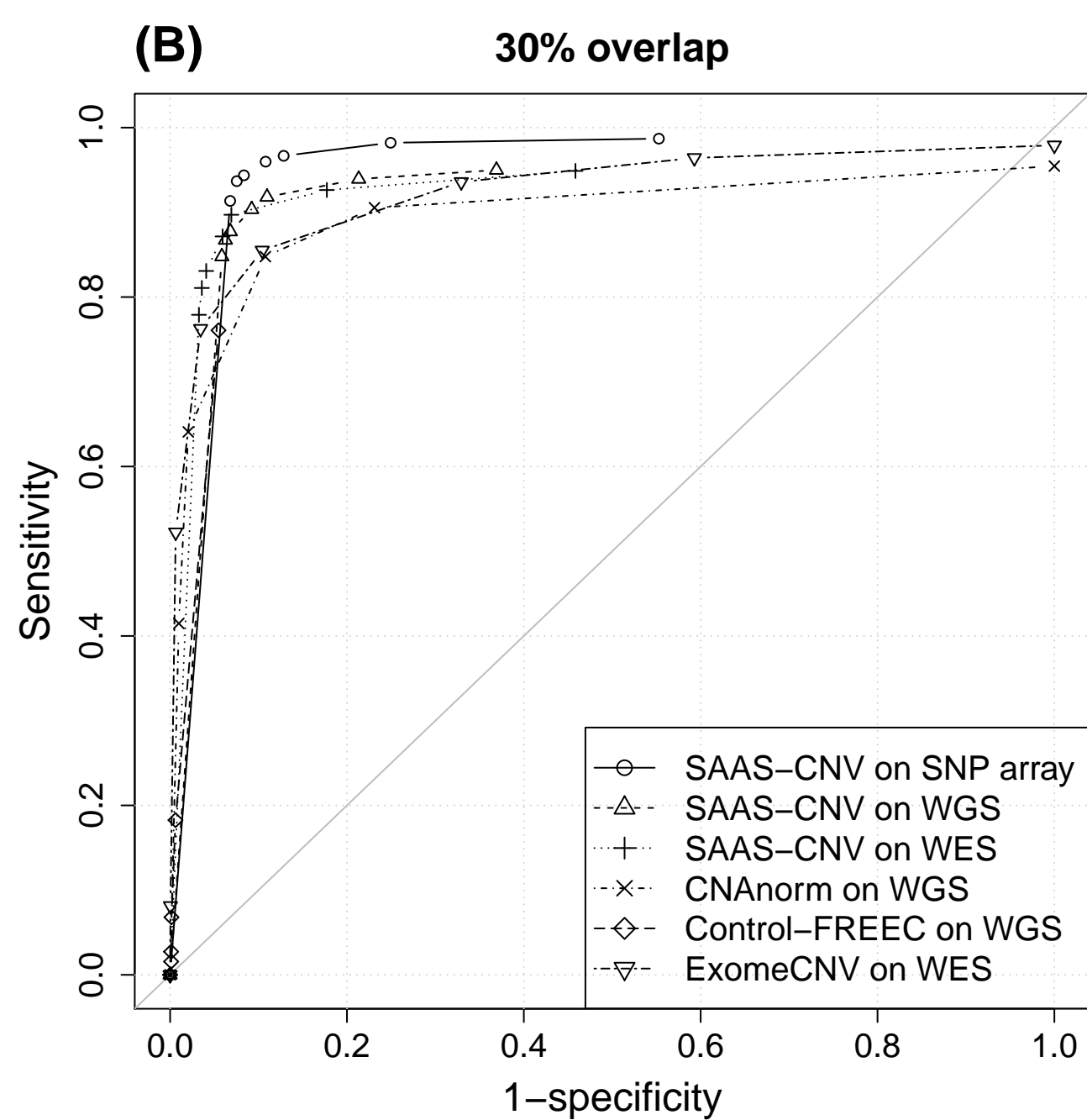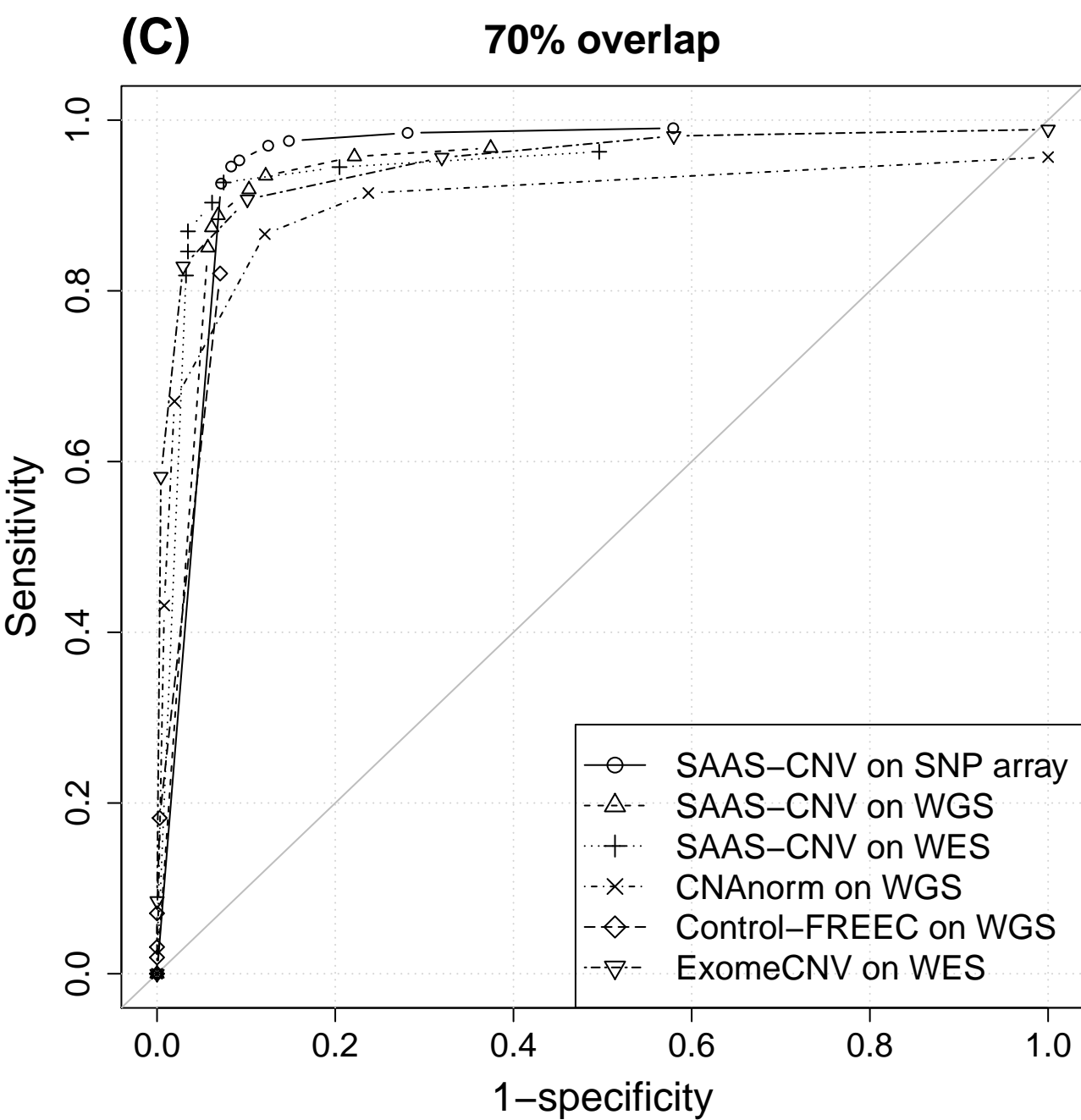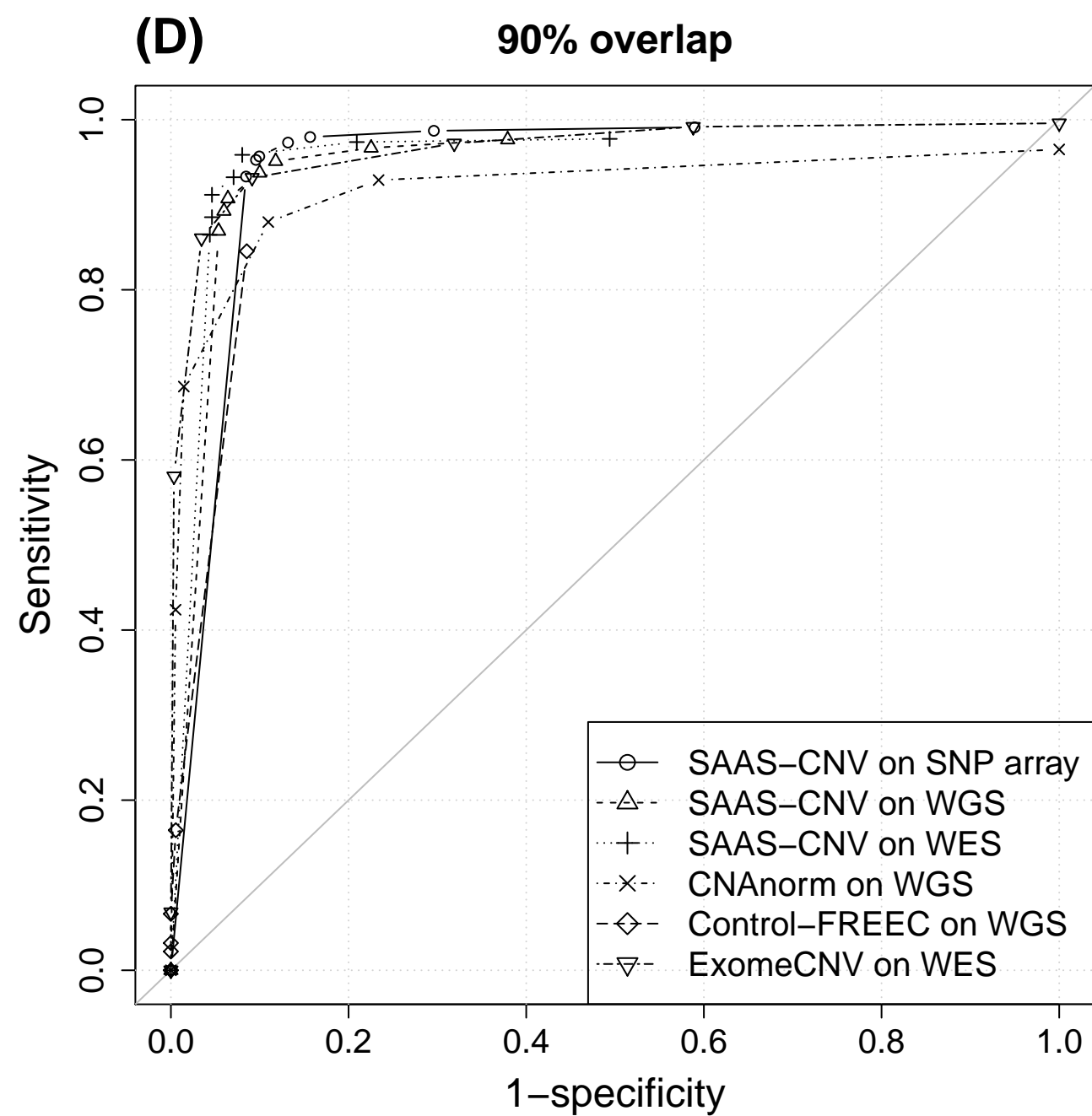

Supplement: S9 Fig — The results from GAP analysis on SNP array data are treated as benchmark. Overlap rate threshold is (A) 10%, (B) 30%, (C) 70% and (D) 90%. (PDF) [file pcbi.1004618.s010.pdf]

**(A) Normal BAF**

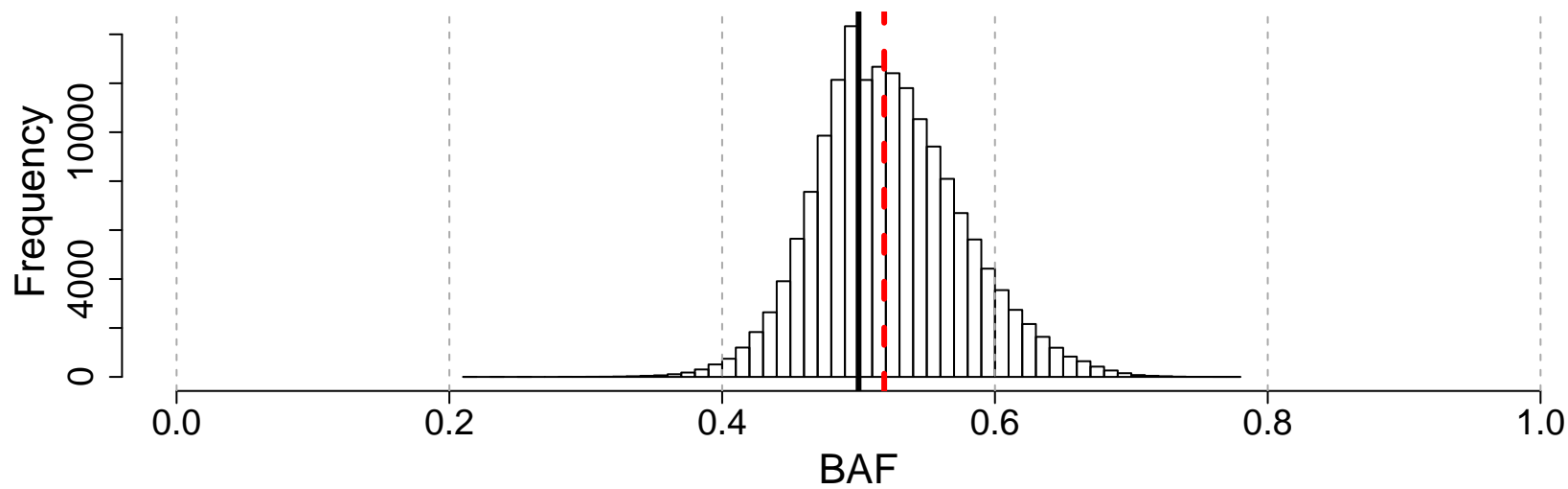

**(B) Tumor BAF**

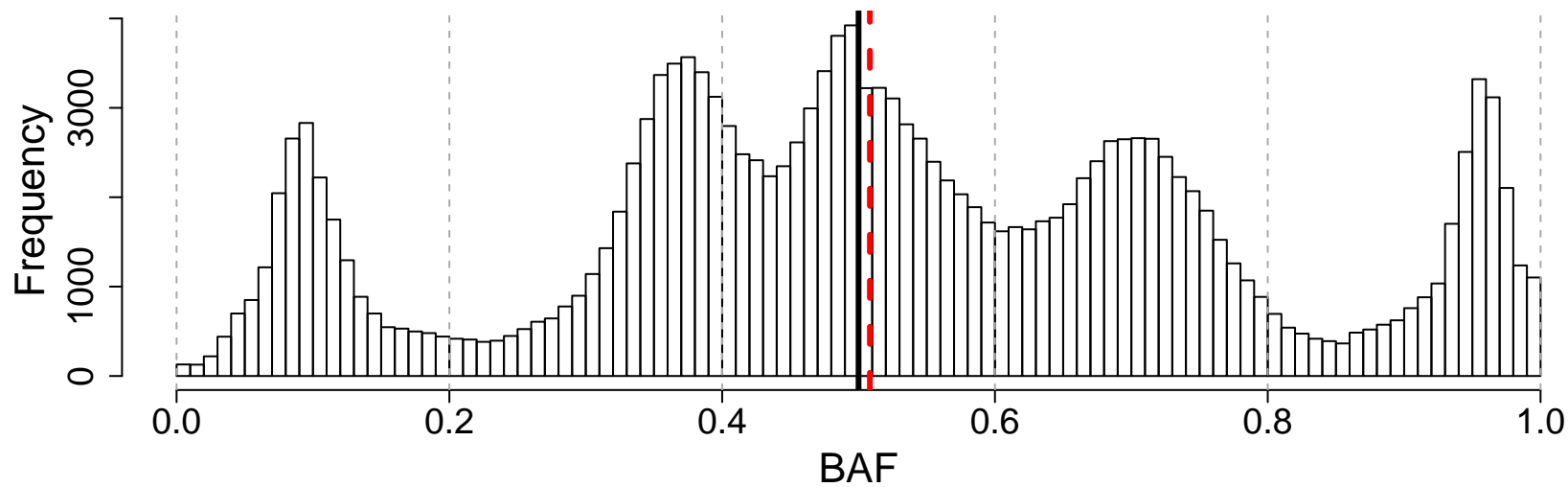

**(C) log2mBAF**

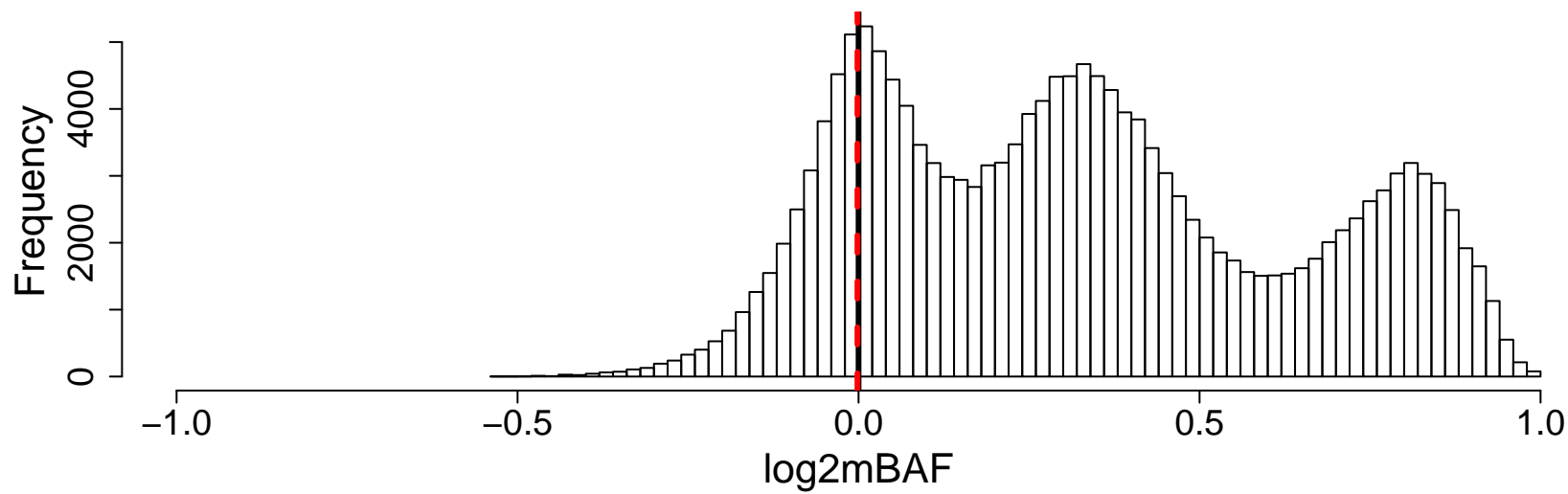

Supplement: S10 Fig — Data is taken from the HKU HCC patient PT116. (A) Normal BAF. (B) Tumor BAF. (C) log2mBAF. Black solid line indicates 0.5 in (A) and (B), and 0 in (C). Red dashed line indicates median values in (A) and (B), and the baseline estimated from the analysis pipeline in (C). Gray dotted lines in (A) and (B) are used as reference to mark the relative location of each peak with respect to the black central line, about which peaks on the left and right are expected to be symmetric. Both tumor and normal BAF signals bias toward B allele. (PDF) [file pcbi.1004618.s011.pdf]

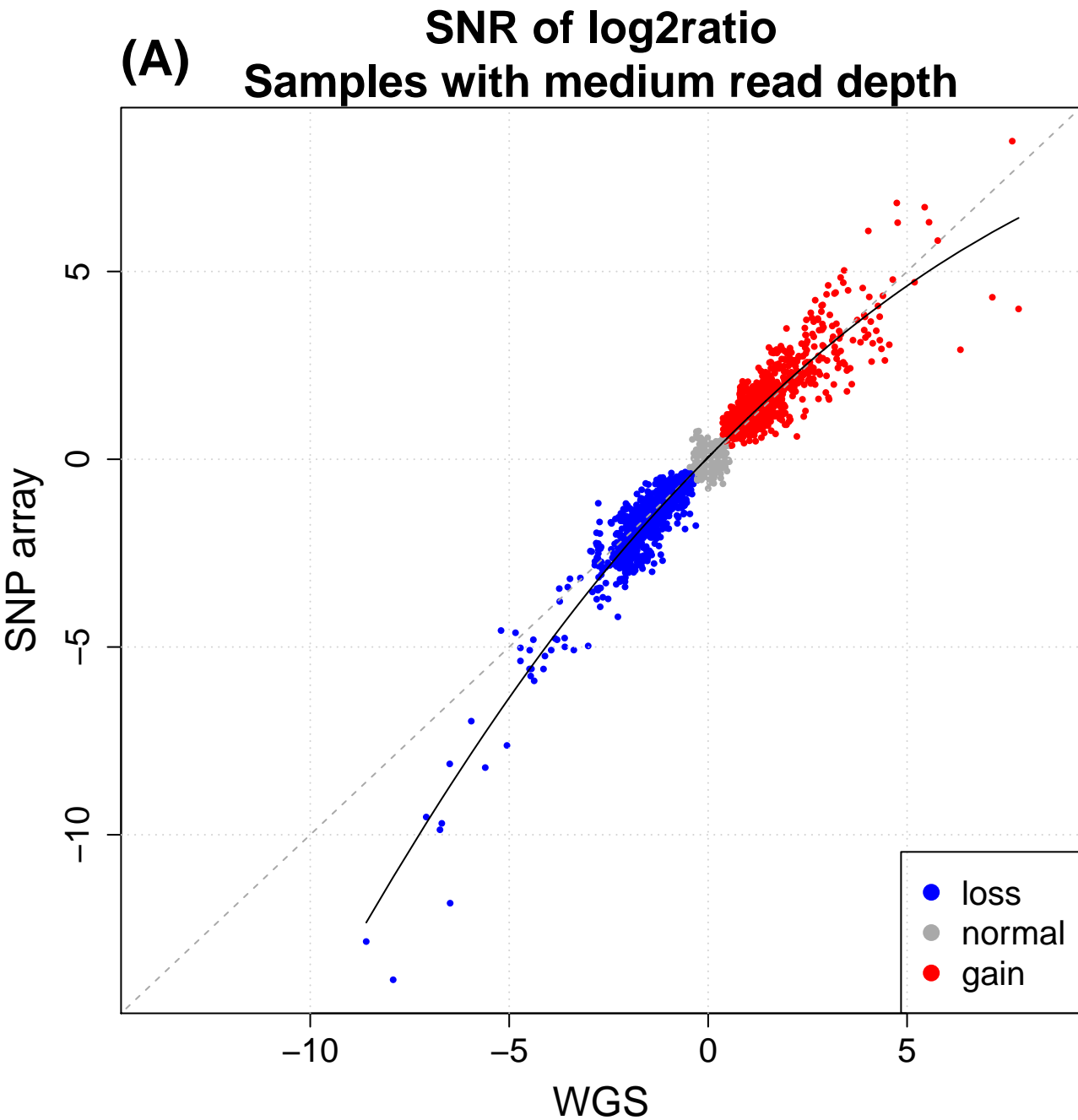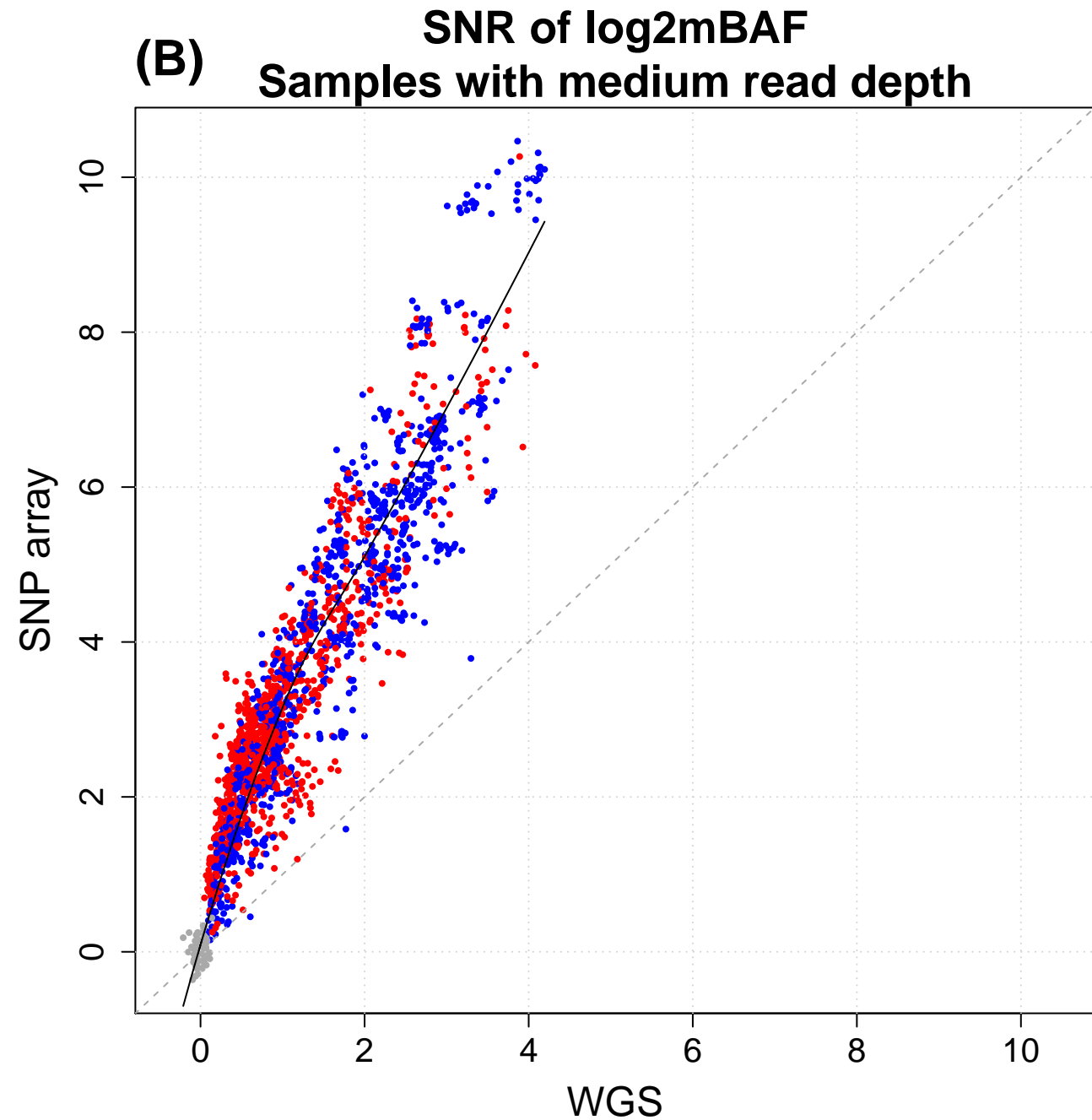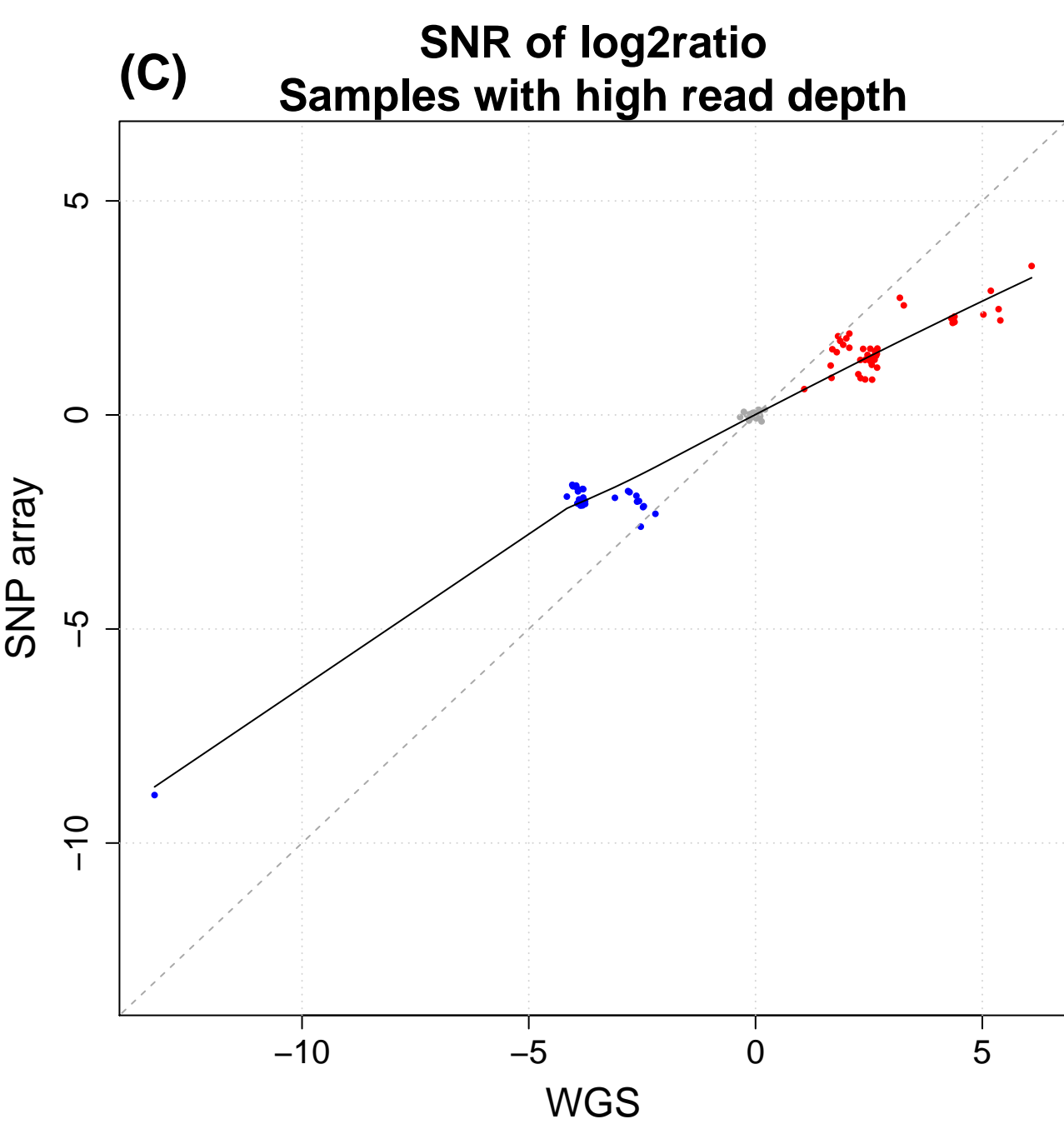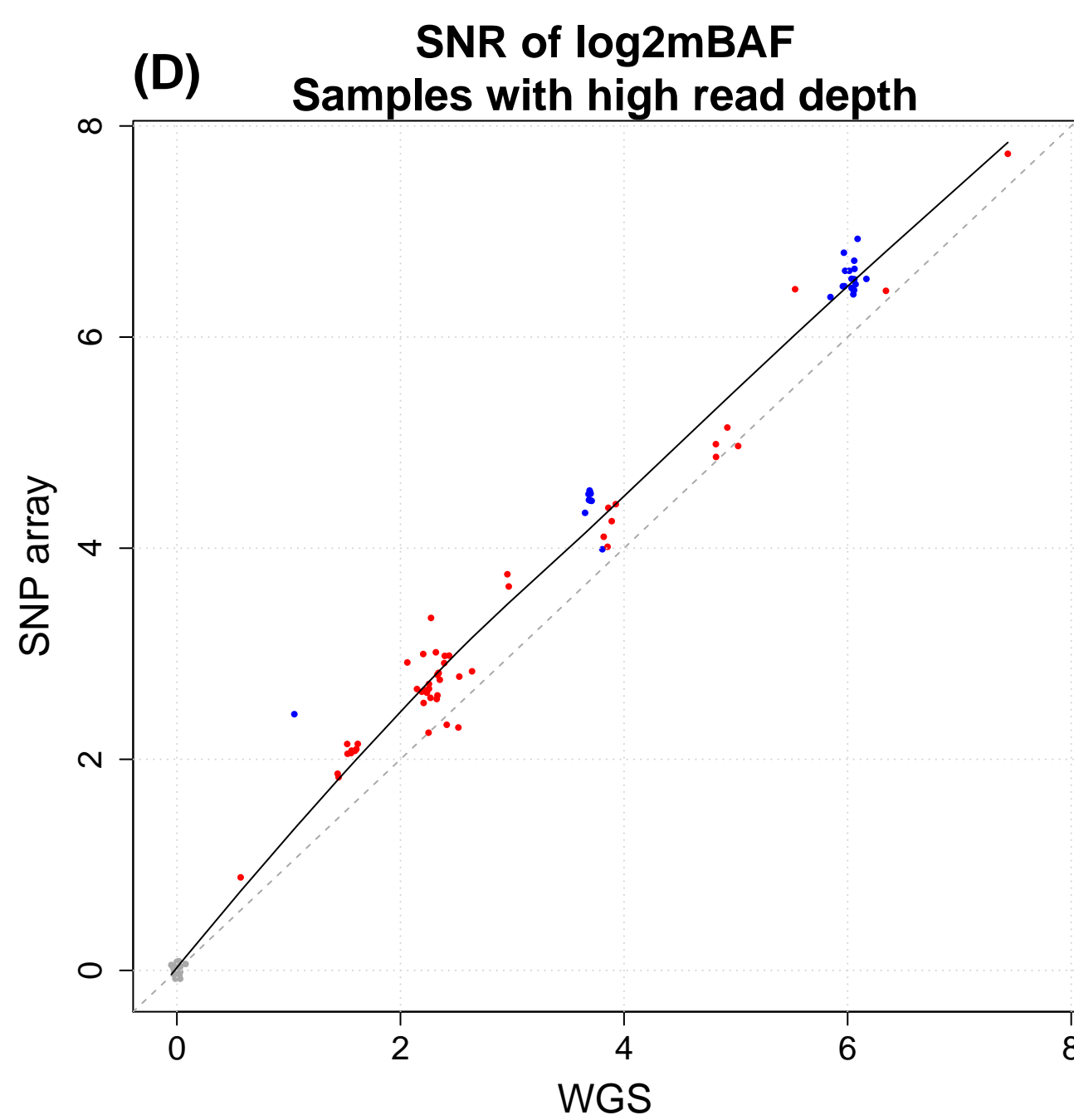

Supplement: S11 Fig — Each dot indicates a genomic segment with concordant copy number status, coded by different colors, and >50% overlap between the results from the two platforms using SAAS-CNV. SNR of log2ratio and log2mBAF is shown in samples with medium RD (n = 86) in (A) and (B), and high RD (n = 2) in (C) and (D), respectively. (PDF) [file pcbi.1004618.s012.pdf]

**(A) Signal of log2ratio**

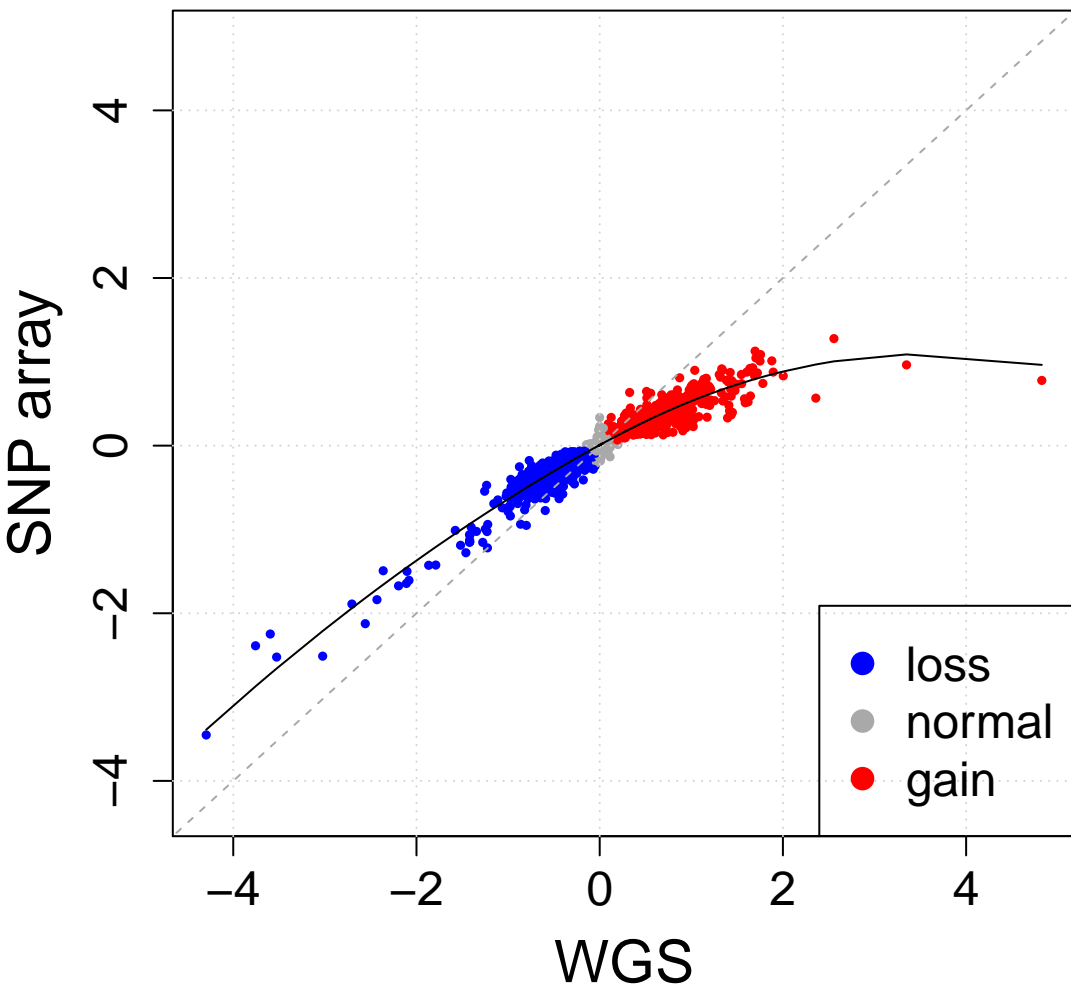

**(B) SNR of log2ratio**

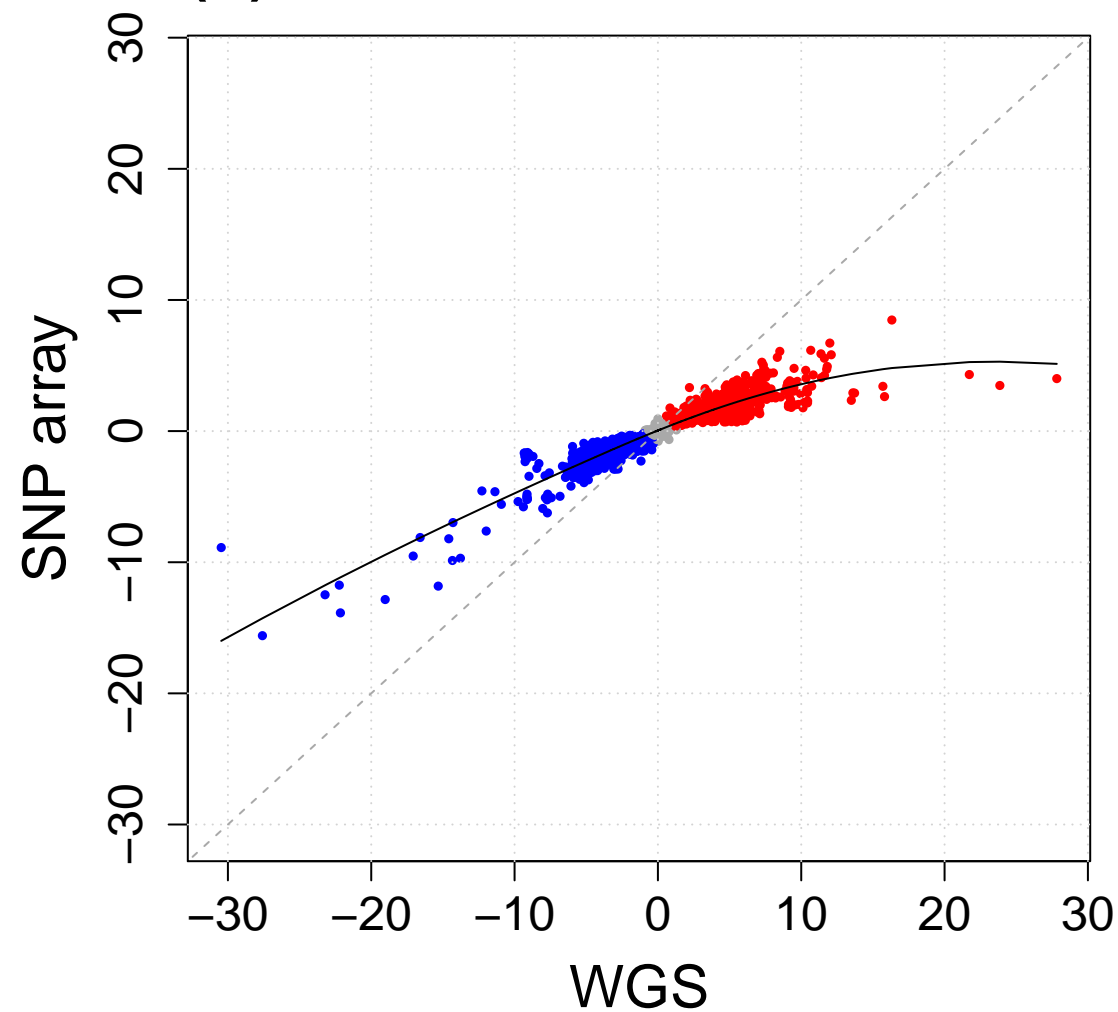

Supplement: S12 Fig — Each dot indicates a genomic segment with concordant copy number status, coded by different colors, and >50% overlap from the results of the two data using CNAnorm. (A) Signal of log2ratio. (B) SNR of log2ratio. (PDF) [file pcbi.1004618.s013.pdf]

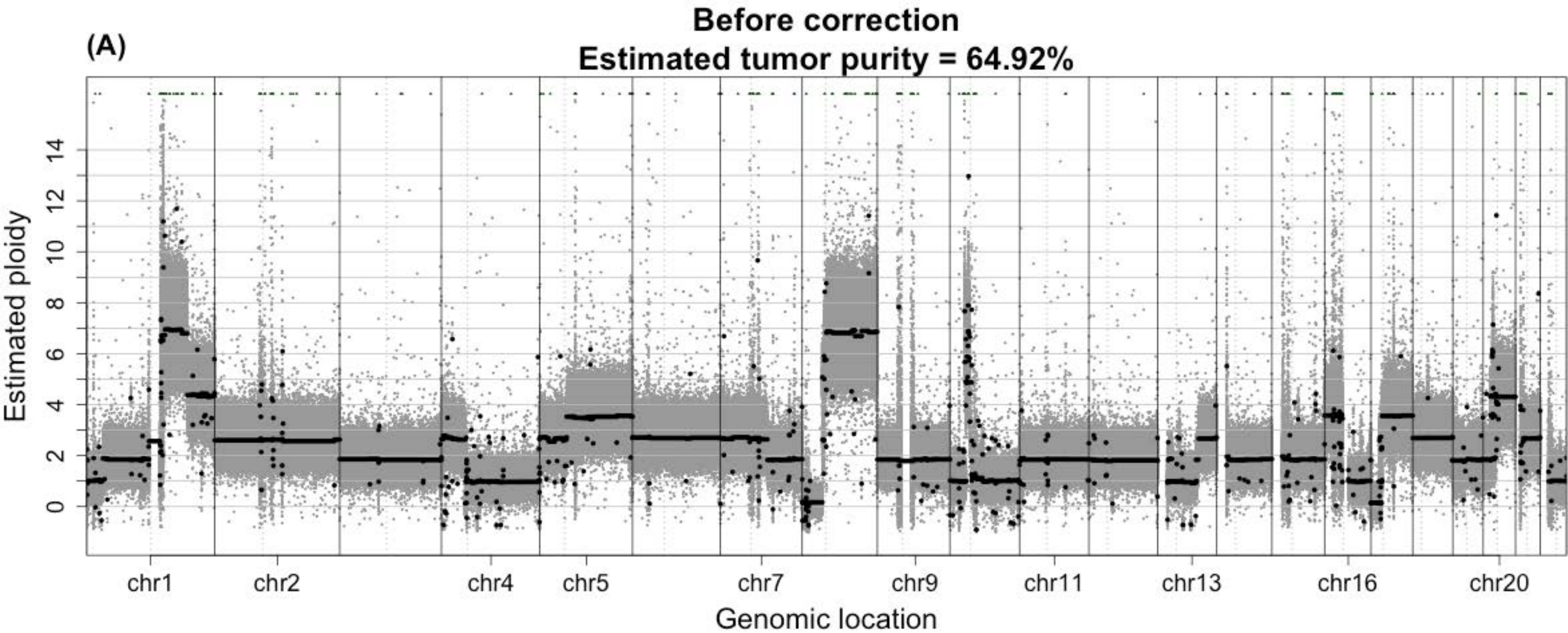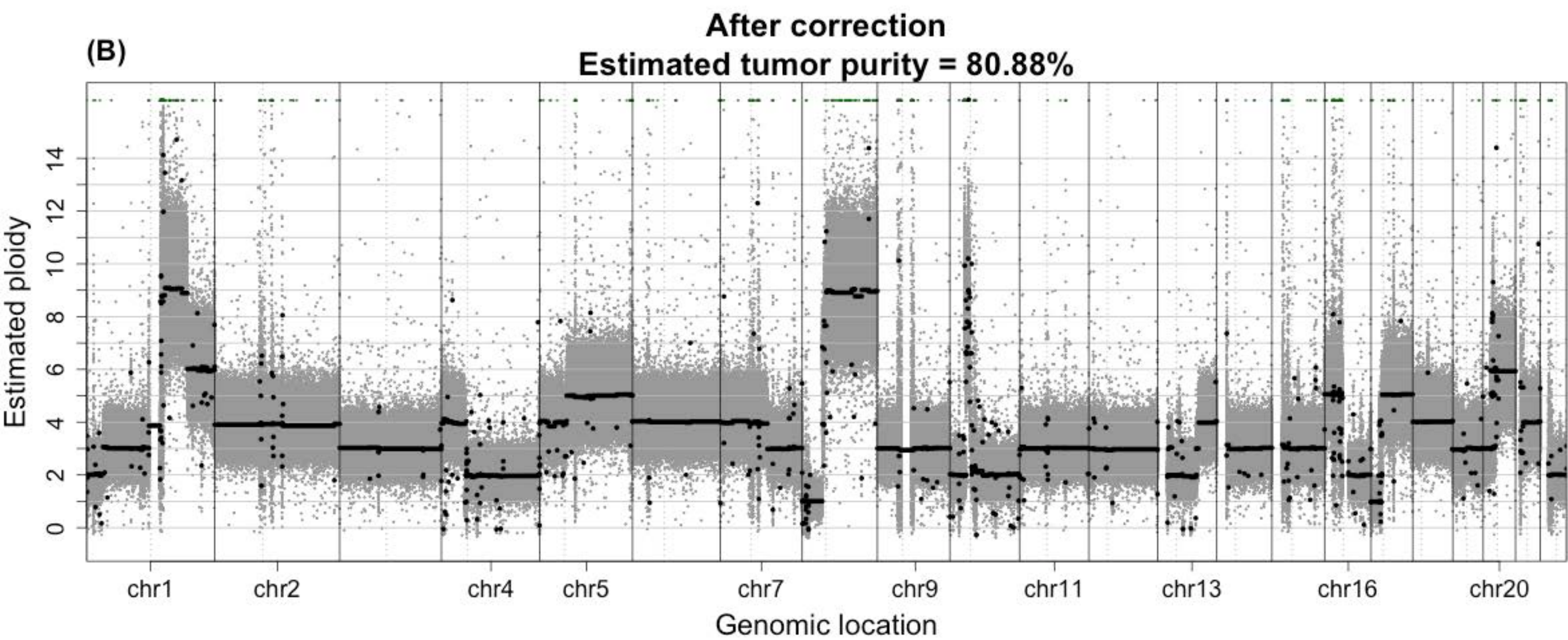

Supplement: S13 Fig — Also see Fig 10 in the main text. The plot was generated by modifying the function plotGenome from R package CNAnorm. (A) Results based on tumor-normal ratio signal alone before manual correction. (B) Results after correction with BAF information accounted for. In each panel, grey dots represent normalized and tumor-purity-corrected tumor-normal ratio per 1kb window; solid thick black lines are the segmented DNAcopy output; green triangulars are points outside the graph; the vertical solid lines separate the chromosomes and the vertical dotted lines indicate the locations of centromeres. (PDF) [file pcbi.1004618.s014.pdf]
