# Supplementary material for: SAAS-CNV: A Joint Segmentation Approach on Aggregated and Allele Specific Signals for the Identification of Somatic Copy Number Alterations with Next-Generation Sequencing Data
Source: PLoS Comput Biol. 2015 Nov 19;11(11):e1004618. doi: 10.1371/journal.pcbi.1004618 (PMC4652904; doi:10.1371/journal.pcbi.1004618)
Supplement: S1 Table — (PDF) [file pcbi.1004618.s015.pdf]

Table S1: Summary metrics for NA18507 WES data

Replicate	# reads	# mapped reads	Average read depth	# heterozygous sites	# supporting reads at heterozygous sites	Average read depth at heterozygous sites
Replicate 1	100303002	99847581	75.5	68639	3955654	57.6
Replicate 2	162639060	161682621	104.6	69003	5516472	79.9
Replicate 3	128585240	127994012	92.5	69568	4792889	68.9
Replicate 4	117686384	109090448	110.1	69247	5286936	76.3
Replicate 5	131111278	130525023	93.8	69718	4847402	69.5
Replicate 6	112387228	111685492	81.5	69554	4244126	61.0
