# Supplementary material for: SAAS-CNV: A Joint Segmentation Approach on Aggregated and Allele Specific Signals for the Identification of Somatic Copy Number Alterations with Next-Generation Sequencing Data
Source: PLoS Comput Biol. 2015 Nov 19;11(11):e1004618. doi: 10.1371/journal.pcbi.1004618 (PMC4652904; doi:10.1371/journal.pcbi.1004618)
Supplement: S2 Table — (PDF) [file pcbi.1004618.s016.pdf]

Table S2: Summary metrics for NA18507 WGS data

Replicate	# reads	# mapped reads	Average read depth	# heterozygous sites	# supporting reads at heterozygous sites	Average read depth at heterozygous sites
Replicate 1 (ERP001231)	1357751670	1306325114	41.6	3176740	133358254[a]	42.0
Replicate 2	1092279026	1059870809	33.8	3259202	105932103	32.5
Replicate 3	1372665360	1327686048	42.3	3260554	128413678	39.4
Replicate 4	2004328248	1940657611	61.9	3262474	184249450	56.5

[a]: The distance between two adjacent heterozygous sites may be within the range of read length, so some of the mapped reads are counted more than once.
