# Supplementary material for: SAAS-CNV: A Joint Segmentation Approach on Aggregated and Allele Specific Signals for the Identification of Somatic Copy Number Alterations with Next-Generation Sequencing Data
Source: PLoS Comput Biol. 2015 Nov 19;11(11):e1004618. doi: 10.1371/journal.pcbi.1004618 (PMC4652904; doi:10.1371/journal.pcbi.1004618)
Supplement: S3 Table — (PDF) [file pcbi.1004618.s017.pdf]

Table S3: Correlation of read depths across loci between pairs of replicates for NA18507 WES data

Index	Synthesized pair	SAAS-CNV						ExomeCNV							
		# het. sites	Normal average read depth[a]	Normal read depth std. dev.	Tumor average read depth[a]	Tumor read depth std. dev.	Correlation[c]	# exons	Normal average read depth[b]	Normal read depth std. dev.	Tumor average read depth[b]	Tumor read depth std. dev.	Correlation[d]	Truncated exons (%)[e]	Truncated correlation[f]
1	Rep1_vs_Rep2	68639	57.6	50.7	80.4	60.5	0.9583	366958	73.7	71.1	101.9	80.8	0.9725	3.96	0.9669
2	Rep1_vs_Rep3	68628	57.6	50.7	69.7	57.2	0.9754	366797	73.7	71.1	90.3	84.7	0.9910	3.74	0.9802
3	Rep1_vs_Rep4	68543	57.7	50.7	76.8	66.3	0.9449	366702	73.7	71.1	107.2	113.0	0.9777	7.17	0.9575
4	Rep1_vs_Rep5	68631	57.6	50.7	70.5	57.9	0.9733	366896	73.7	71.1	91.6	85.9	0.9899	3.91	0.9781
5	Rep1_vs_Rep6	68591	57.7	50.7	61.7	53.0	0.9785	366744	73.7	71.1	79.6	76.6	0.9906	2.78	0.9807
6	Rep2_vs_Rep3	69003	79.9	60.6	69.2	57.3	0.9673	367068	101.8	80.8	90.3	84.7	0.9728	4.41	0.9676
7	Rep2_vs_Rep4	68907	80.0	60.6	76.3	66.4	0.9484	367042	101.9	80.8	107.1	113.0	0.9457	7.38	0.9429
8	Rep2_vs_Rep5	69038	79.9	60.6	70.0	58.0	0.9655	367145	101.8	80.8	91.5	85.9	0.9710	4.54	0.9649
9	Rep2_vs_Rep6	68974	80.0	60.6	61.3	53.1	0.9595	367060	101.9	80.8	79.5	76.6	0.9698	4.06	0.9644
10	Rep3_vs_Rep4	69568	68.9	57.1	75.8	66.3	0.9598	366852	90.3	84.7	107.1	113.0	0.9797	7.22	0.9607
11	Rep3_vs_Rep5	69697	68.8	57.1	69.6	57.8	0.9832	366965	90.3	84.7	91.6	85.9	0.9932	4.10	0.9843
12	Rep3_vs_Rep6	69621	68.8	57.1	60.9	52.9	0.9806	366878	90.3	84.7	79.6	76.6	0.9928	3.75	0.9837
13	Rep4_vs_Rep5	69247	76.3	66.4	70.1	57.9	0.9626	366959	107.1	113.0	91.6	85.9	0.9811	7.21	0.9637
14	Rep4_vs_Rep6	69189	76.4	66.4	61.4	53.0	0.9536	366839	107.1	113.0	79.6	76.6	0.9809	7.17	0.9618
15	Rep5_vs_Rep6	69718	69.5	57.9	60.8	53.1	0.9808	366975	91.6	85.9	79.6	76.6	0.9929	3.91	0.9837

[a]: Average read depth over heterozygous sites.
[b]: Average read depth over exons.
[c]: Pearson correlation between the pair of read depth across heterozygous sites.
[d]: Pearson correlation between the pair of read depth across the exome.
[e]: The percentage of the exons with read depth > 250 in either tumor or normal, which are excluded from the calculation of truncated correlation.
[f]: Pearson correlation between the pair of read depth across the exome excluding those with excessive read depth. This is the correlation shown in Figure 4 of the main text.
